# A five-cuproptosis-related LncRNA Signature: predicting prognosis, assessing immune function & drug sensitivity in lung squamous cell carcinoma

**DOI:** 10.7150/jca.82370

**Published:** 2023-05-21

**Authors:** Hongtao Zhao, Lei Wu, Qinyuan Liao, Peiluo Huang, Ruonan Sun, Xiuzhen Yang, Juan Du

**Affiliations:** 1 Department of Immunology, College of Basic Medicine, Guilin Medical University, Guilin 541199, Guangxi, China.; 2 College of continuing education, Guilin Medical University, Guilin 541004, China.; 3 College of pharmacy, Guilin Medical University, Guilin 541199, China.; 4 Department of clinical laboratory, Zibo Central Hospital, Zibo 255036, China.

**Keywords:** Cuproptosis, LncRNA, LUSC, Prognostic model, Drug sensitivity

## Abstract

Lung squamous cell carcinoma has so far lacked effective targets for diagnosis and treatment. In cancer research, long noncoding RNAs (LncRNAs) emerge as novel therapeutic targets and biomarkers. Cuprophosis is a new death type involving multiple biological processes in tumor cells. Here, we aimed to explore whether Cuprophosis-related lncRNAs could be used to predict prognosis, assess immune function, and test drug sensitivity in LUSC patients. The Cancer Genome Map (TCGA) was used to obtain genome and clinical data, and Cuprophosis-relevant genes were found in the literature. A cuproptosis-related lncRNA risk model was built using co-expression analysis, univariate/multivariate Cox regression, and LASSO analysis. The survival analysis was used to assess the model's prognostic value. The univariate and multivariate Cox regression analyses were performed to determine whether risk score, age, gender, or clinical stages could be used as independent prognostic factors. Gene Set Enrichment Analysis and mutation analysis were performed on differentially expressed mRNA between high-risk and low-risk groups. The (TIDE) algorithm was used to conduct immunological functional analysis and drug sensitivity testing. Five cuproptosis-related LncRNAs were identified, and the selected LncRNAs constructed a prognosis model. According to the Kaplan-Meier survival analysis, the overall survival time for patients in the high-risk group was shorter than for those in the low-risk group. For LUSC patients, the risk score serves as an independent prognostic indicator. The GO and KEGG enrichment analysis revealed that the differentially expressed mRNAs between the high- and low-risk groups were enriched in several immune-related processes. The enrichment score of differentially expressed mRNAs in the high-risk group is higher than that of the low-risk group in multiple immune function pathways, including the IFN-γ and MHC I pathways. The Tumor Immune Dysfunction and Exclusion (TIDE) test revealed that the high-risk group was more likely to experience immune escape. The drug sensitivity analysis showed that patients with low-risk ratings were likely to respond to GW441756 and Salubrinal. In contrast, patients with higher risk scores were more responsive to dasatinib and Z-LLNIe CHO. The 5-Cuprophosis-related lncRNA signature can be used to predict prognosis, assess immune function, and test drug sensitivity in LUSC patients.

## Introduction

Lung cancer is the most prevalent malignancy and one of the most prominent reasons for high cancer-related mortality worldwide 1. The most common subtypes of non-small cell lung cancers (NSCLCs) are lung squamous cell carcinoma (LUSC) and lung adenocarcinoma (LUAD). Meanwhile, LUSC represents approximately 30% of NSCLC cases 2, 3. The primary treatment for patients with early-stage NSCLC is surgical tumor removal. However, for patients with advanced stages of LUSC, targeted therapy is frequently required 4. Even though a handful of the LUSC's oncogenic drivers have been identified, the disease lacks more targeted treatment therapies 5. Therefore, it is crucial to identify novel treatment targets for LUSC patients.

Copper (Cu) is one of the essential metal elements for most living organisms. It involves many biological processes, including mitochondrial respiratory, Fe uptake, anti-oxidant, and detoxification 6. Furthermore, intracellular or extracellular Cu ion dyshomeostasis leads to oxidative stress and cytotoxicity 7. Excess Cu concentrations in the tricarboxylic acid (TCA) cycle have recently been discovered to cause cell death, which differs from all previous modalities of cell death. This phenomenon is known as "cuproptosis," according to Tsvetkov et al. 8, 9. Copper level changes in patients' serum and tumor tissues have been discovered 10. Cu ion encourages tumor growth and metastasis by stimulating angiogenesis 11, 12. Therefore, studies on copper-associated genes are revealing new pathways of tumorigenesis and progression.

Long noncoding RNAs (LncRNAs) are defined as RNA molecules with longer than 200 nucleotides in length and do not possess protein-coding properties 13. LncRNA is a component of the transcriptome that can regulate the expression of specific genes based on cell type, developmental stage, and cell function 14. The role of LncRNAs in gene expression regulation occurs at both the transcriptional and posttranscriptional levels 15. For a long time, tens of thousands of LncRNA were discovered to be abnormally expressed or mutated in various cancer types 16. The abnormal expression or mutation of LncRNA influences tumor occurrence, metastasis, and stage 17. As previously stated, these phenomena occurred primarily because LncRNAs can regulate microRNA (miRNA) and messenger RNA (mRNA) involved in cancer progression 18. As a result, LncRNAs are emerging as novel therapeutic targets and biomarkers in cancer research.

Our study discovered differentially expressed cuproptosis-related LncRNAs in LUSC tissues using the Cancer Genome Atlas (TCGA) database. Furthermore, we developed a prognostic prediction model for predicting overall survival (OS) in LUSC patients using cuproptosis-related LncRNAs. The cuproptosis-related LncRNAs pattern could be used as a prognostic indicator for LUSC patients and could provide vital information for cancer therapy.

## Materials and methods

### Screen cuproptosis-related LncRNAs

LUSC gene expression data, clinical data, and mutation data were obtained from The Cancer Genome Atlas (TCGA) data portal (https://tcga-data.nci.nih.gov/tcga/). Our analysis contains 551 samples, including 502 LUSC samples and 49 LUSC adjacent normal tissues samples. The 19 cuproptosis-related genes were identified through a literature review (Supplementary Table 1). The Pearson correlation coefficient test picked up a total of 291 cuproptosis-related LncRNAs associated with 19 genes (the 'limma' R package). We created a Sankey map for cuproptosis-related LncRNA-mRNA co-expression network using the ggalluvial package in R version 4.1.3.

### Model building

To determine a cuproptosis-related LncRNA signature, we first used univariate Cox analysis to screen LncRNAs related to overall survival for LUSC. Subsequently, cuproptosis-related LncRNA with P < 0.05 (n = 23) in the univariate Cox analyses were selected for the least absolute shrinkage and selection operator (LASSO) regression analysis and multivariate Cox regression analyses. The Risk Scores were generated using a multivariate Cox regression coefficient. Risk Score = coef(LncRNA AC002467.1) × (LncRNA AC002467.1)expr + coef(LncRNA MIR3945HG) × (LncRNA MIR3945HG)expr + coef(LncRNA AC010328.1) × (LncRNA AC010328.1)expr + coef(LncRNA AC008972.2) × (LncRNA AC008972.2)expr + coef(LncRNA AC253536.6) × (LncRNA AC253536.6)expr. The correlation between 5 cuproptosis-related LncRNAs and 19 cuproptosis-related mRNAs was analyzed by the Pearson correlation coefficient.

### Model evaluation

Patients lacking enough survival information (n = 6) or with a survival time of 0 days (n = 2) were excluded. Data that shared a clinical patient number (n = 1) were also consolidated into a single item. As a result, 493 samples (Supplementary Table 2) altogether were used in the clinical survival study. LUSC patients were classified into high-risk and low-risk groups according to their median risk score. Overall survival (OS) and progression-free survival (PFS) were analyzed by the Kaplan-Meier method. PFS datasets were gained from UCSC Xena (http://xena.ucsc.edu/). We also calculated the area under the ROC curve (AUC) used to assess the accuracy of the Risk Score. Afterward, the LUSC research samples were randomly divided into training (n = 198) and testing (n = 295) sets according to the ratio of 2:3. The AUC analysis was performed to verify the suitability of the Risk Score in two separate sets. The ROC curve showed with the R software 'timeROC' package. LUSC patients were randomized using the 'caret' R package. Differences in training and testing sets were compared using the chi‐square test.

### Propensity Score Matching (PSM) analysis

For PSM analysis, we used R's 'wakefield,' 'MatchIt,' 'knitr,' 'captioner,' 'tidyverse,' and 'foreign' packages to reduce selection bias between high- and low-risk groups. Patients who met the following criteria were excluded: patients with incomplete age, sex, clinical stage, and survival information. The PSM study covered 484 patients in total. Gender, age, and clinical stage were used as factors in a logistic regression model with 1:1 exact matching to produce propensity scores.

### Independent prognostic analysis

The univariate Cox and multivariate Cox analyses were performed based on risk score, gender, age, and clinical stage. Independent prognostic analysis was conducted using the 'survival' R package. Principal component analysis (PCA) was utilized to show the distribution of the low-risk and high-risk groups using the 'limma' and 'scatterplot3d' R packages. Survival analyses of different clinical stage LUSC patients were explored using the 'survival' and 'survminer' R package.

### Nomogram construction

A nomogram for the prediction of LUSC patients was constructed based on Risk Score, age, gender, and clinical stage. Moreover, calibration curves can be used to evaluate the coherence of intended and actual results. Display the images above using the 'survival,' 'regplot,' and 'rms' R packages. The specific information of the variables for the nomogram constructions is listed in Supplementary Table 3.

### Gene Set Enrichment Analysis (GSEA)

Screening criteria for the differential expression mRNAs (DEmRNAs) were |log2FC| ≥ 1 and p-value < 0.05. Analysis was done using the 'limma' R package. Gene ontology (GO) and Kyoto encyclopedia of genes and genomes (KEGG) enrichment analysis of DEmRNAs was performed by 'clusterProfiler,' 'org.Hs.eg.db', 'ggpubr' and 'RColorBrewer' R package. GO terms and KEGG pathways with p < 0.05 were considered significantly enriched. Our study chose 10 GO terms, and 18 KEGG pathways with the smallest P-value were used to display.

### Tumor mutation burden (TMB) analysis, Immune-related functional analysis, and pharmaceutical screening

'Maftools' R package was used to visualize and analyze tumor mutation burden. To immunization score 493 patients with LUSC who had complete clinical information, the 'limma,' 'GSVA,' and 'GSEABase' software packages, as well as the "ssgsea" method, were used. The R software package 'pheatmap' was used to visualize the result, and P<0.05 was considered statistically significant (*, P < 0.05; **, P < 0.01; ***, P < 0.001). NSCLC dysfunction and exclusion scores were gained from TIDE (http://tide.dfci.harvard.edu/). Analysis of TIDE scores in high and low-risk groups using 'limma' and 'ggpubr' R packages. For analysis of therapeutic agents and drug sensitivity, we use the 'pRRophetic,' 'ggpubr,' and 'ggplot2' R packages with p-value <10^-12^.

### Cell culture and quantitative real-time PCR (qRT-PCR)

Lung squamous cell carcinoma cell lines SK-MES-1 (# FH0040) and NCI-H226 (# FH0041) cells were purchased from Fuheng Biotechnology Co., Ltd. (FuHeng Cell Center, Shanghai, China). Under rigorous sterile conditions at 37°C and 5% CO_2_, NCI-H226 was grown in Gibco 1640 medium (USA) containing 10% fetal bovine serum (BBI, Shanghai, China) and 1% penicillin/streptomycin combination (Solaibao, Beijing, China), and SK-MES-1 was grown in Gibco DMEM medium (USA) containing 10% fetal bovine serum and 1% penicillin/streptomycin combination. Following a 24-hour treatment with 30 nM elesclomol (MedChemexpress Co., LTD. # HY-12040) and Cucl_2_ (Sigma-Aldrich. # 10125-13-0) (1:1), the cells were detected using qRT-PCR. TRIzol (Invitrogen, USA) was used to extract total RNA from the pretreated cells. With the use of the PrimeScript® RT Reagent Kit (TaKaRa, Japan), RNA was reverse-transcribed into cDNA. Lastly, using β-actin as a reference, qRT-PCR was utilized to detect the expression of lncRNAs linked to cuproptosis-related using TB Green Premix Ex Taq II (TaKaRa, Japan). Table 1 lists the primers for lncRNAs relevant to cuproptosis. Each sample was examined three times to determine the levels of lncRNA expression. The 2^-(△CT sample-△CT control)^ was employed to investigate the differential expression of lncRNAs.

### Statistical Analysis

Kaplan-Meier survival analysis was performed with the log-rank test. Differential mRNA expression analysis was performed using the Wilcoxon rank sum test. Furthermore, the analysis of therapeutic agents and drug sensitivity was also evaluated by the Wilcoxon rank sum test. On the other hand, the correlation between drug and Risk Score was analyzed using Spearman correlation. The GraphPad Prism 8 software was used to analyze the qRT-PCR data. The p-values were determined using a t-test, and the findings were presented as Mean ± SEM. The results were considered statistically significant when p < 0.05.

## Results

### Construction of a cuproptosis-related LncRNA Risk Model for LUSC

**Figure 1:**
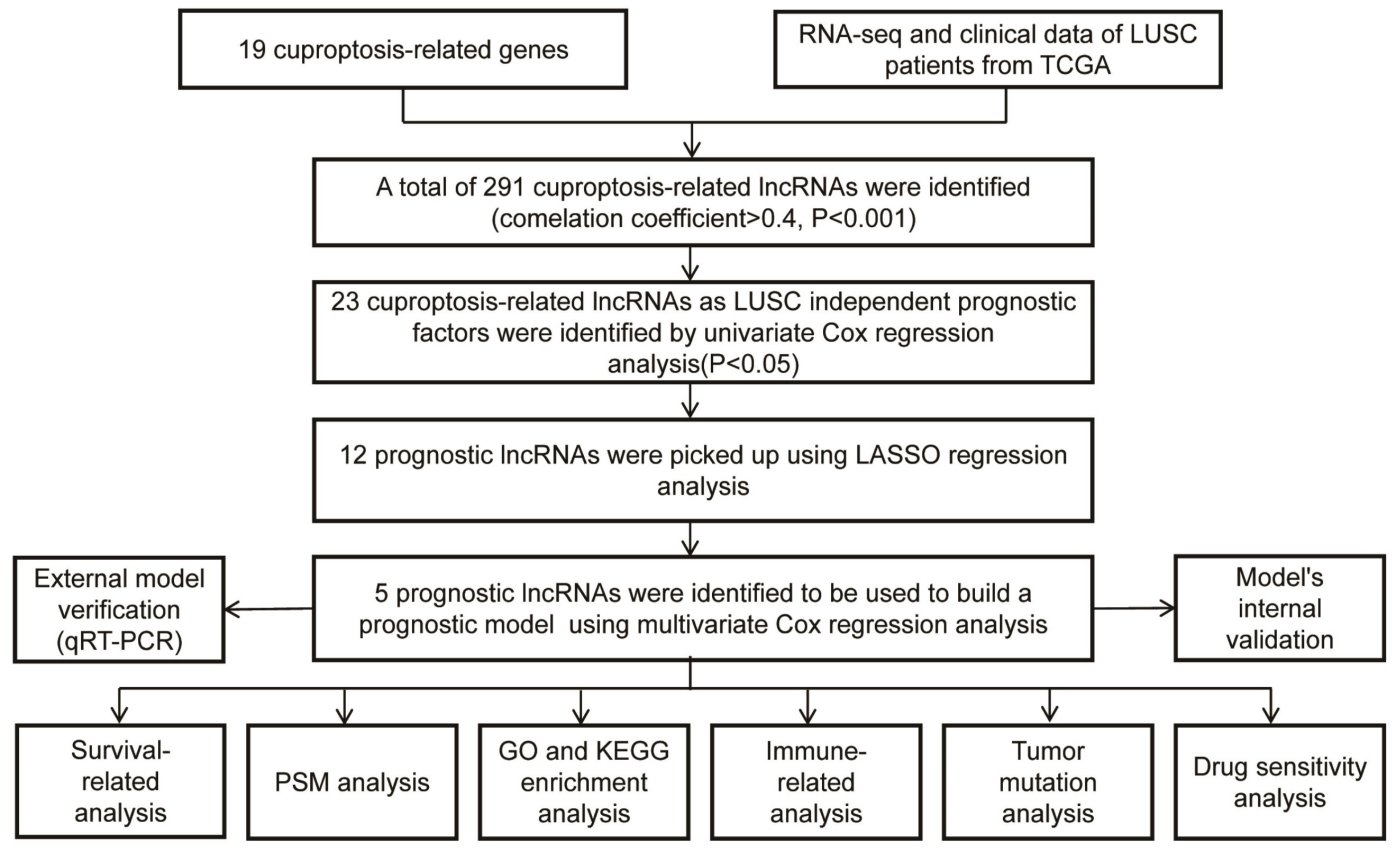
The diagram of the research design.

**Figure 2:**
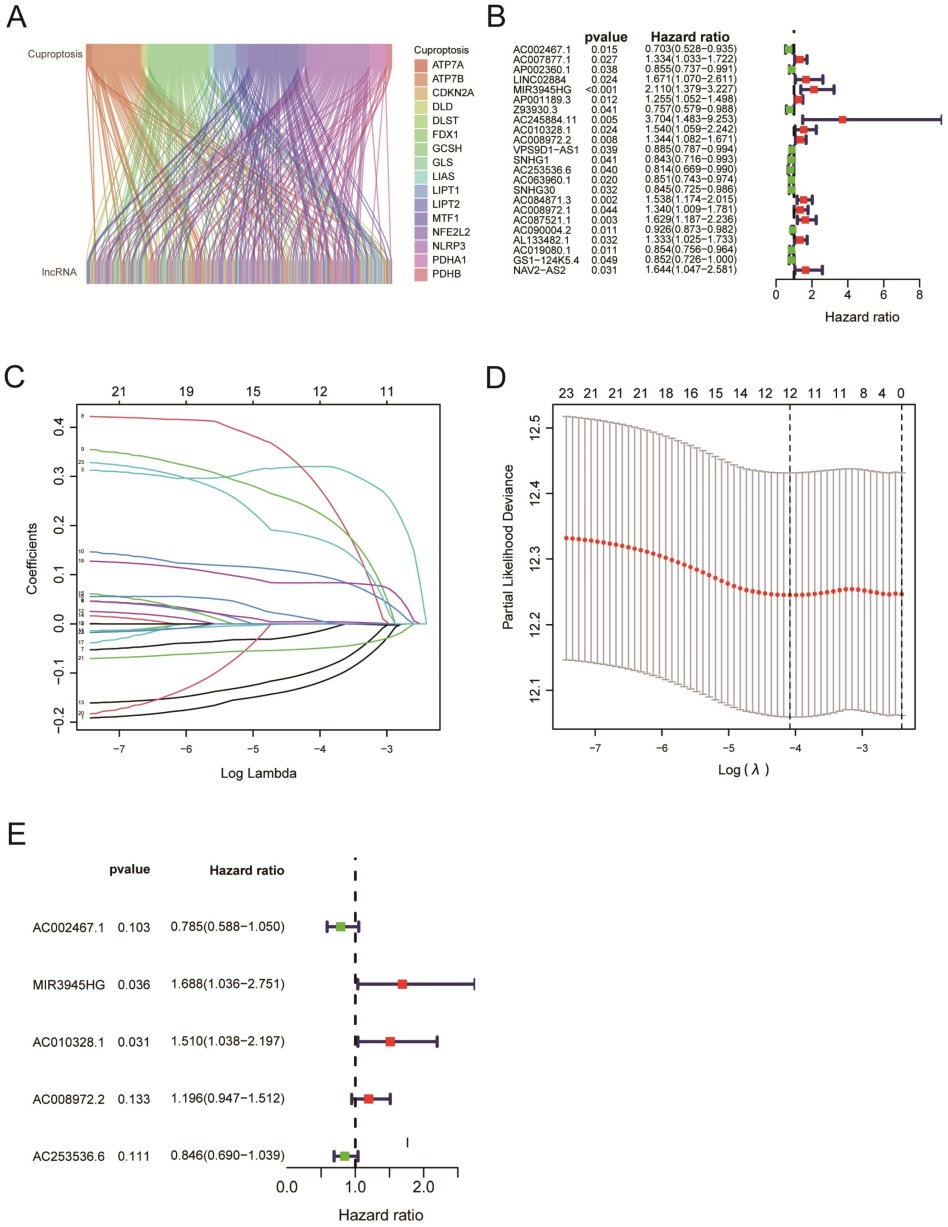
Construction of a cuproptosis-related LncRNA Risk Model for LUSC. A. Co-expression of 16 mRNAs and 291 lncRNAs relevant to cuproptosis was visualized using a Sankey diagram. B. The forest plot showed 19 cuproptosis-related lncRNAs chosen by univariate Cox regression analysis. Red nodes represented high-risk lncRNAs. Green nodes expressed low-risk lncRNAs. C. The LASSO regression analysis showed that each independent variable's trajectory varies. D. Cuproptosis-related lncRNAs were selected using LASSO regression. E. Multivariate Cox analysis was used to find five cuproptosis-related lncRNAs and create the risk model.

The workflow of our studies is illustrated in Figure 1. RNA-seq data for 502 LUSC and 49 normal samples were obtained from the TCGA database. First, 291 cuproptosis-related LncRNAs were identified based on 19 known cuproptosis-related mRNAs (Supplementary Table 1). A total of 291 cuproptosis-related LncRNAs associated with 19 cuproptosis-related genes were picked up by the Pearson correlation coefficient test using the 'limma' R package. The criteria for LncRNA selection is based on P < 0.001 and correlation coefficient > 0.4 ​(Figure 2A). Of the 291 cuproptosis-related LncRNAs, 23 cuproptosis-related LncRNAs (P < 0.05) as LUSC independent prognostic factors were identified by univariate Cox regression analysis​ (Figure 2B, P < 0.05). Subsequently, to precisely establish the prognostic model, LASSO analysis was performed to pick up 12 LncRNAs from 23 LncRNAs for the subsequent analyses (Figure 2C, D). Finally, multivariate Cox proportional hazards regression analysis identified five cuproptosis-related LncRNAs used to build a prognostic model (Figure 2E). Risk Score = (-0.24159 × EXP AC002467.1) + (0.523456 × EXP MIR3945HG) + (0.41216 × EXP AC010328.1) + (0.17936 × EXP AC008972.2) + (-0.16671 × EXP AC253536.6).

### Survival analysis using 5-cuproptosis-related lncRNA risk model

**Figure 3:**
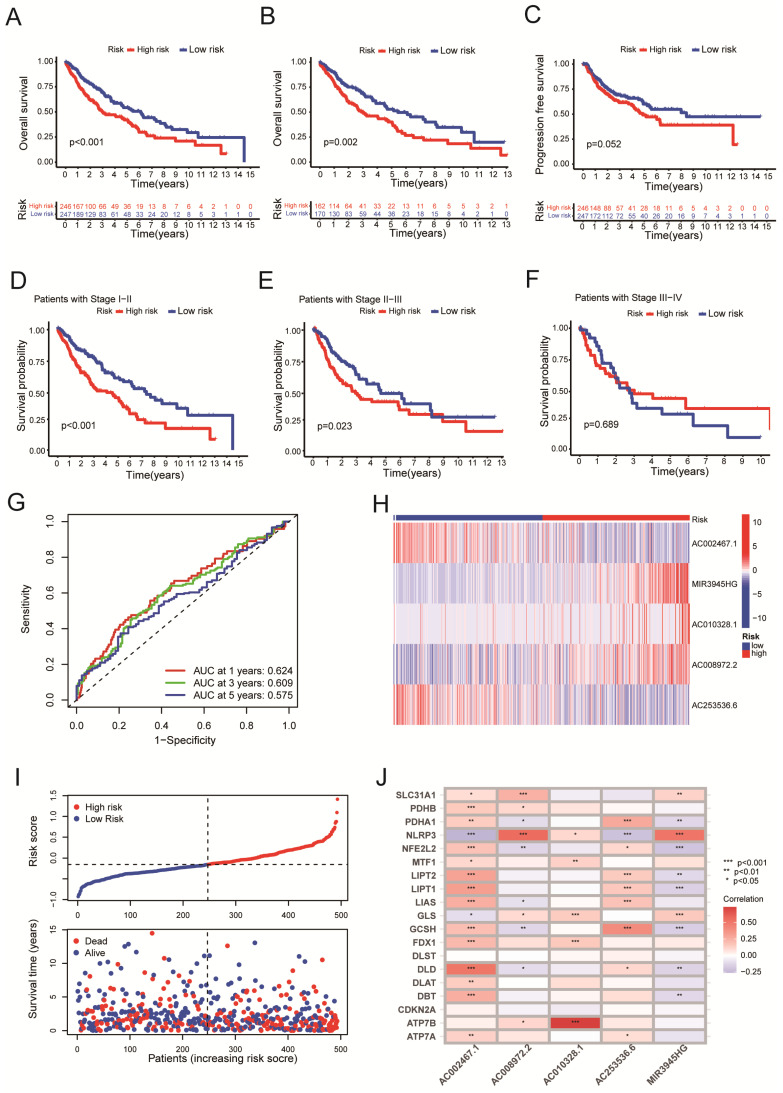
Survival analysis using 5-cuproptosis-related lncRNA risk model. A. The overall survival (OS) curves for LUSC patients (n = 493). B. The overall survival analysis after PSM (n = 332). C. The progression-free survival (PFS) curves for LUSC patients (n = 473). D-F. For clinical stage I and II patients (D), stage II and III patients (E), and stage III and IV patients (F), the overall survival of the high-Risk group and the low-Risk group was shown. G. Overall survival ROC curves at 1, 3, and 5 years. H. The Heat map displayed the expressions of 5 cuproptosis-related lncRNAs. I. The distribution of Risk Score, risk groups, and survival status for 493 LUSC patients was conducted. J. The heat map illustrated the correlations between the 5-cuproptosis-related lncRNAs and 19-cuproptosis-related genes. Statistics were considered significant when the p-value was less than 0.05.

Four hundred ninety-three patients with LUSC were grouped into low-risk or high-risk groups based on the median Risk Score. The Kaplan-Meier survival analysis revealed that the overall survival time for patients in the high-risk Score group was poorer than those in the low-Risk Score group (Figure 3A, P < 0.001). To eliminate selection bias between high-risk and low-risk groups, we recruited 484 lung squamous cell carcinoma patients with complete follow-up information for PSM analysis between high-risk and low-risk groups. After PSM analysis using 'MatchIt' R software package, 170 patients in the low-risk group and 162 patients in the high-risk group were selected. The selection bias between high-risk and low-risk groups before and after PSM analysis was shown in Table 2.

Reanalyzing the survival assessment of high and low risk groups after eliminating bias by PSM revealed that the overall survival rate of the high-risk group was considerably lower than that of the low-risk group (Figure 3B, P = 0.002). The results above indicated the risk score has an impact on a patient's prognosis for lung squamous cell carcinoma. Furthermore, the PFS analysis was performed based on the TCGA and UCSC Xena databases. Although patients in the high-Risk Score group presented with worse PFS compared with those in the low-Risk Score group, there was no statistical difference between the two groups (Figure 3C, P = 0.052). For clinical stage I-II patients, overall survival of the high-Risk Score group was lower than that of the low-Risk Score group (Figure 3D, P < 0.001). Similarly, for clinical stage, II-III LUSC patients, the patients in the low-Risk Score group had a significantly higher survival probability compared with those in the high-Risk Score group (Figure 3E, P = 0.023). Due to the small number of LUSC patients in clinical stages III-IV, no statistical difference was observed between the high-Risk Score group and the low-Risk Score group (Figure 3F, P = 0.689). The AUC values for 1‐year, 3‐year, and 5‐year reached 0.624, 0.609, and 0.575, respectively, which indicated that the cuproptosis-related LncRNAs' prognostic signature had a good predictive ability (Figure 3G). The Heat map displayed the expressions of 5 cuproptosis-related LncRNAs (Figure 3H). The distribution of Risk Scores, risk groups, and survival status for 493 LUSC patients are shown in Figure 3I. Figure 3J shows we analyzed the correlations between the 5-cuproptosis-related LncRNAs and 19-cuproptosis-related genes.

### Validation of the risk model

**Figure 4:**
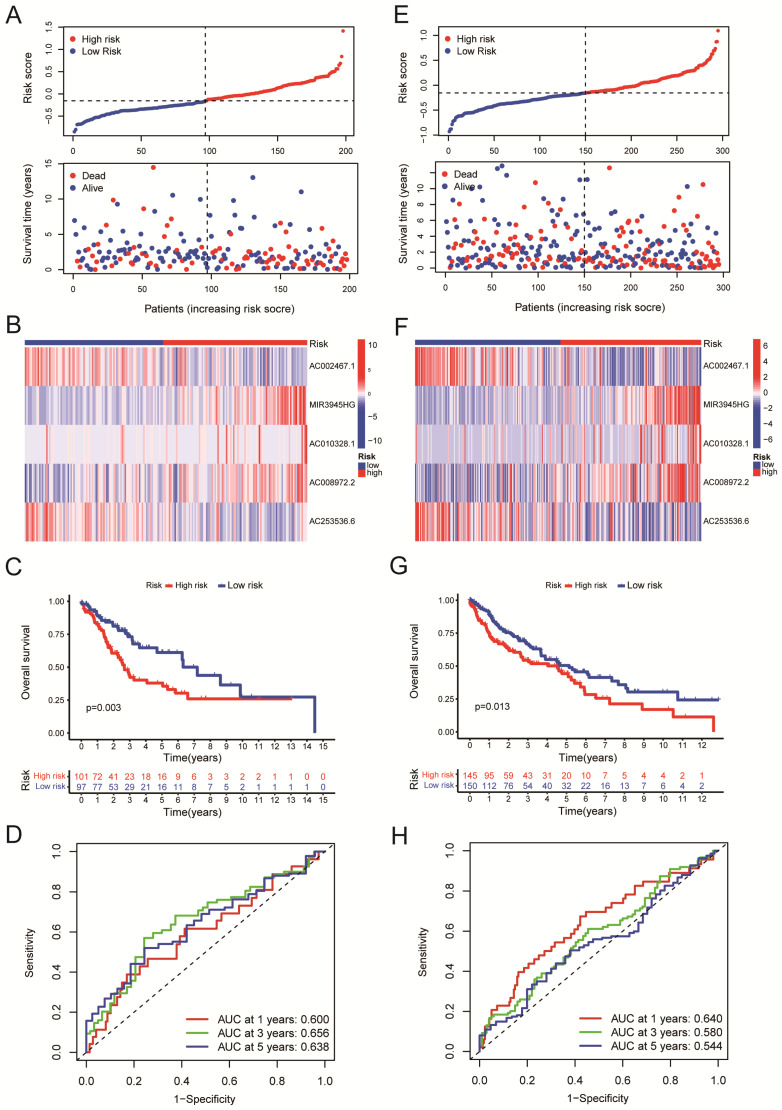
Validation of the risk model. A. The upper panel showed the distributions of high-risk and low-risk LUSC patients in the training set. The lower panel displayed the relationship between the patient's survival time, survival status, and Risk Score in the training set. B. The heat map displayed the 5-cuproptosis-related lncRNA expressions in the training set. C.The overall survival of the high-Risk and low-Risk groups was shown for the training set. D. The AUC value for the training set predicting OS at 1, 3, and 5 years were 0.600, 0.656, and 0.638, respectively. E. The upper panel showed the distributions of high-risk and low-risk LUSC patients in the testing set. The lower panel displayed the relationship between the patient's survival time, survival status, and Risk Score in the testing set. F. The heat map displayed the 5-cuproptosis-related lncRNA expressions in the training set. G. The overall survival of the high-Risk group and the low-Risk group for the testing set was demonstrated. H. The value of AUC for the testing set predicting OS at 1, 3, and 5 years were, respectively, 0.640, 0.580, and 0.544.

**Figure 5:**
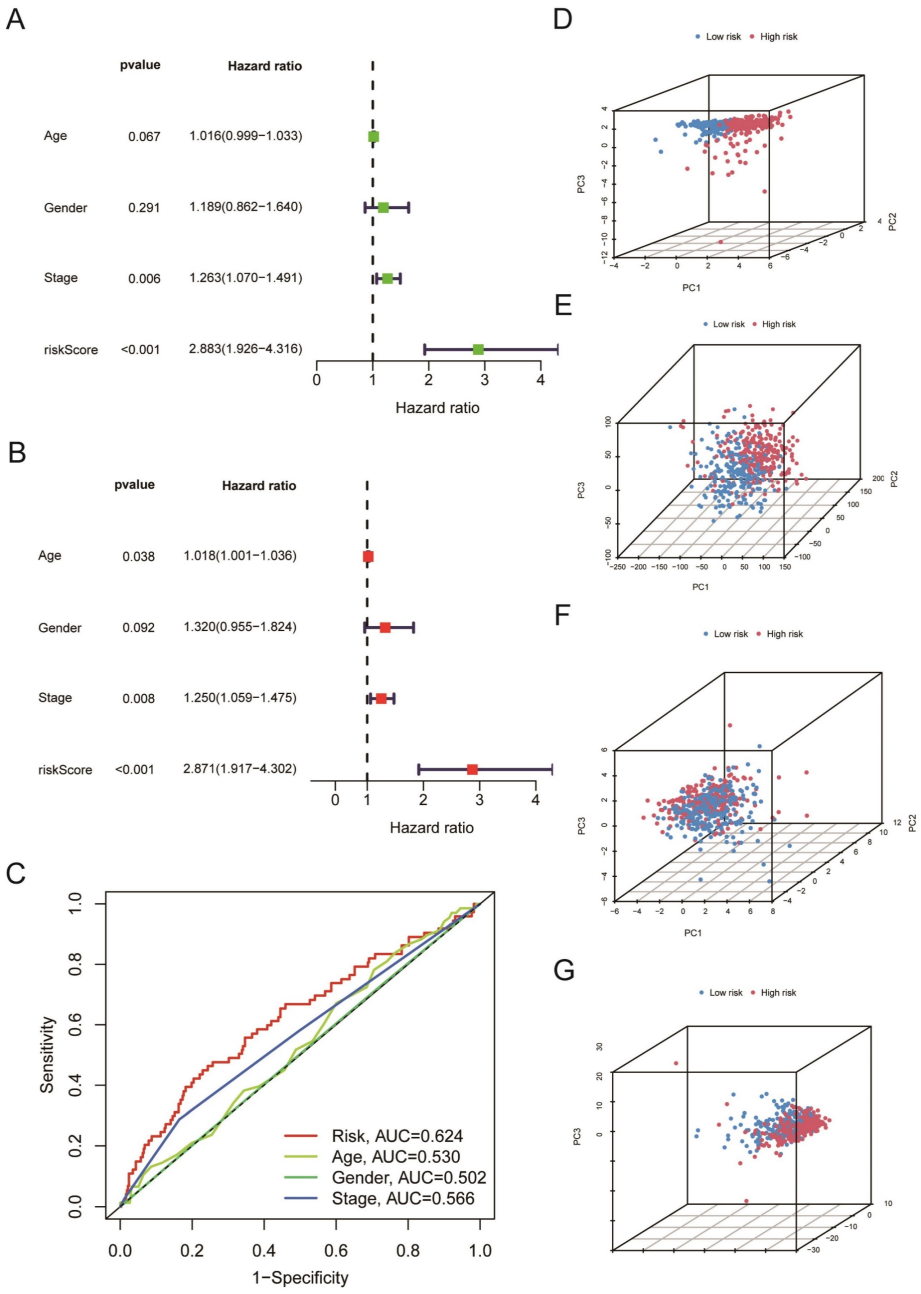
The risk score serves as an independent prognostic indicator for LUSC patients. A-B. (A) Univariate and (B) Multivariate Cox regression analysis confirmed that the Risk Score and clinical stages of patients with LUSC were independent prognostic variables. C. the AUC for Risk Score, clinical stage, age, and gender is 0.624, 0.566, 0.530, and 0.502, respectively. D-G. 5-cuproptosis-related LncRNAs (D), all genes (E), 19 cuproptosis-related mRNAs (F), and 291 cuproptosis-related LncRNAs (G) were subjected to the principal component analysis. Statistics were considered significant when the p-value was less than 0.05.

We randomly divided the LUSC patients into two groups: the training group (n = 198) and the testing group (n = 295), with a 2:3 split ratio. The Chi-square test showed no difference between the two randomized groups (Table 3). The training and testing sets were divided into high-risk and low-risk groups based on the median risk score. The upper panel of figure 4A shows the distributions of LUSC patients in the training set. The lower panel of Figure 4A displayed the relationship between the patient's survival time, survival status, and Risk Score in the training set. The 5-cuproptosis-related LncRNA expressions of the training set are shown in Figure 4B. For the training set, LUSC patients in the high-risk group had significantly shorter overall survival than those in the low-risk group (Figure 4C, P = 0.003). The AUC value for the training set predicting OS at 1, 3, and 5 years were 0.600, 0.656, and 0.638, respectively (Figure 4D). Figures 5E-F showed the distributions of LUSC patients with Risk Score, survival time, survival status (Figure 4E), and 5-cuproptosis-related LncRNA expressions (Figure 4F) in the testing set. Moreover, Kaplan-Meier survival analysis showed that the training set's outcomes were similar to those of the testing set (Figure 4G, P = 0.013). The value of AUC for the testing set predicting OS at 1, 3, and 5 years were, respectively, 0.640, 0.580, and 0.544 (Figure 4H).

### The risk score serves as an independent prognostic indicator for LUSC patients

We further used univariate Cox regression analysis (Figure 5A, P < 0.05) and multivariate Cox regression analysis (Figure 5B, P < 0.05) to study whether risk score, age, gender, and clinical stages can be used as prognostic factors. We discovered that the Risk Score and clinical stages of patients with LUSC were independent prognostic variables (Figure 5A, B, P < 0.05). After that, ROC curve analysis found that the AUC for Risk Score, clinical stage, age, and gender is 0.624, 0.566, 0.530, 0.502, respectively (Figure 5C); that is, our proposed risk model possessed the best ability in prediction the survival time for LUSC patients. According to the risk score, we performed PCA to observe further the distribution of patients in the high-risk and low-risk groups. As shown in Figure 5D, when the 5-cuproptosis-related LncRNAs were applied to the principal component analysis (Figure 5D), the patients were divided into high-risk and low-risk groups. However, when all genes (Figure 5E, n = 59427), 19 cuproptosis-related mRNAs (Figure 5F), and 291 cuproptosis-related LncRNAs (Figure 5G) were subjected to the principal component analysis, respectively, cases from the high-risk and low-risk groups were combined, making it impossible to classify them.

### Construction and validation of a Nomogram

**Figure 6:**
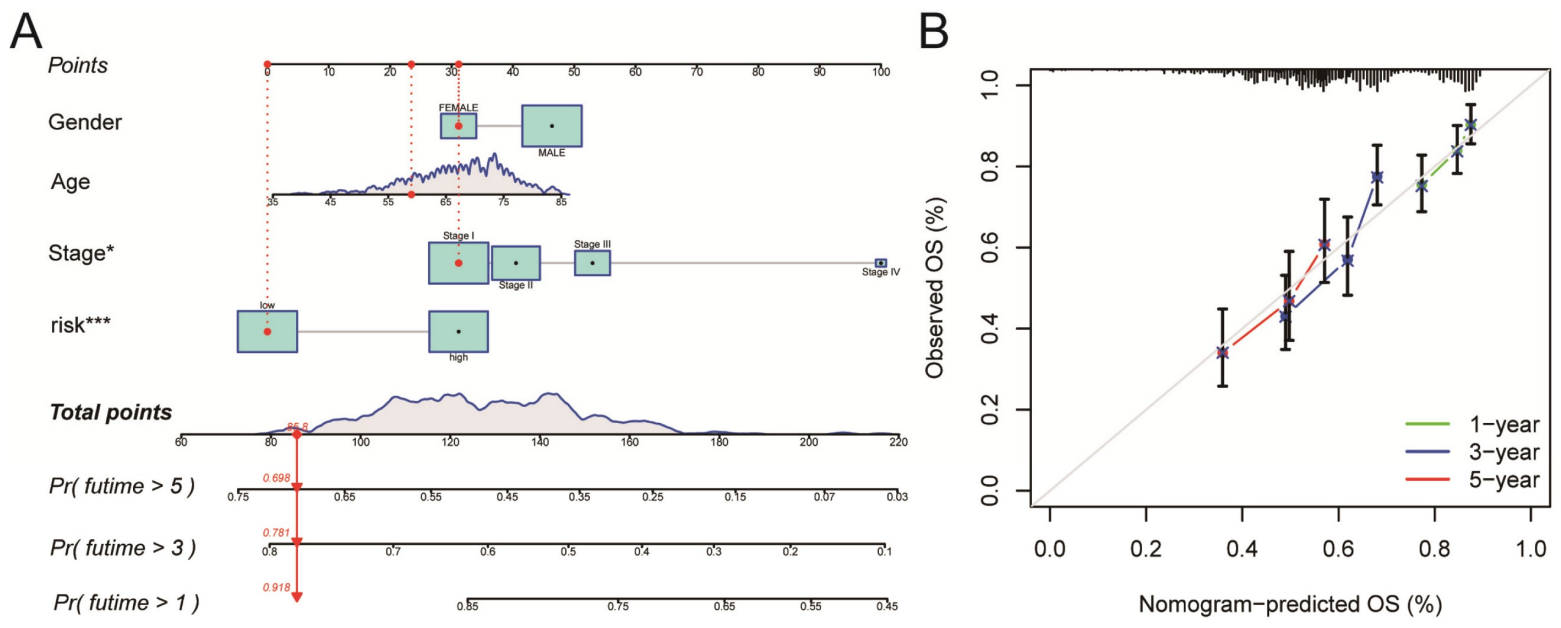
Construction and validation of a Nomogram. A. A nomogram for LUSC patients was constructed based on age, gender, clinical stage, and Risk Score. Gender: gender of patient; Age: age of patient; Stage: clinical stage of the patient; risk: risk score based on the risk model. B. Calibration curves showed that the nomogram-predicted OS for 1-year, 3-year, and 5-year was generally consistent with the corresponding observed OS for LUSC patients.

A nomogram for LUSC patients was constructed based on age, gender, clinical stage, and Risk Score (Figure 6A). Calibration curves showed that the nomogram-predicted OS for 1-year, 3-year, and 5-year was generally consistent with the corresponding observed OS for LUSC patients (Figure 6B).

### Gene Set Enrichment Analysis and mutation analysis of LUSC patients

**Figure 7:**
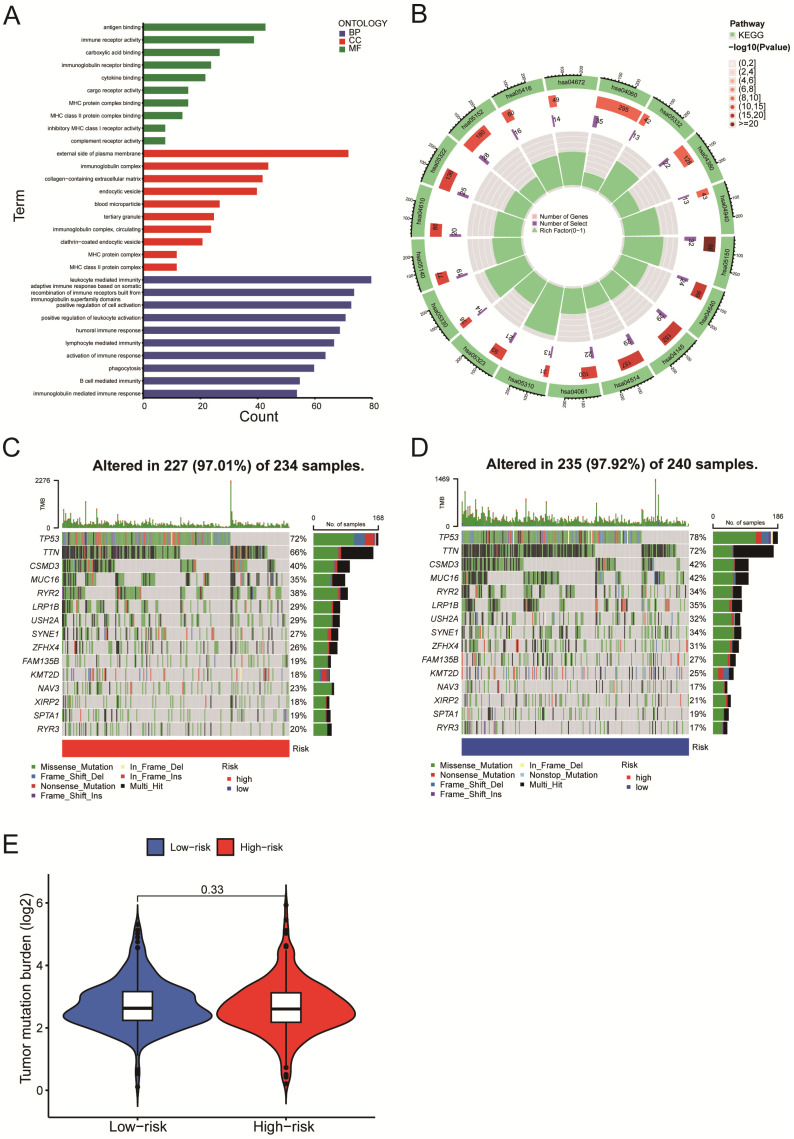
Gene Set Enrichment Analysis and mutation analysis of LUSC patients. A-B. The differentially expressed mRNAs between the high- and low-risk groups of LUSC patients were examined using the GO enrichment analysis (A) and the KEGG enrichment analysis (B). C. A waterfall plot was used to display the top 15 mutant genes of LUSC in the high-risk (234 samples) categories. D. A waterfall plot was used to display the top 15 mutant LUSC genes in the low-risk (240 samples) categories.

We next evaluated the differentially expressed mRNAs between the high-and low-risk groups of LUSC patients. According to the GO enrichment analysis, the differentially expressed mRNAs were enriched in several immune-related processes, including antigen binding, immunoglobulin complex formation, and humoral immune response (Figure 7A). The KEGG enrichment analysis indicated that the differentially expressed mRNAs were enriched in asthma (hsa05310), phagosome (hsa04145), cell adhesion molecules (hsa04514), complement & coagulation cascades (hsa04610), etc. (Figure 7B). We identified the top 15 genes with the highest mutation frequency and visualized them using the 'Maftools' R package. The top 15 genes had mutations in 97% (227/234) of the high-risk category samples (Figure 7C) and 97.92% (235/240) of the low-risk category samples (Figure 7D). According to the analysis of tumor mutational burden (TMB), there was no statistically significant difference in total mRNA mutation between high-risk and low-risk patients (Figure 7E). As a result, variations in survival rates between high-risk and low-risk groups might not be caused by mutations in these genes.

### Immune-related functional analysis and drug sensitivity analysis

**Figure 8:**
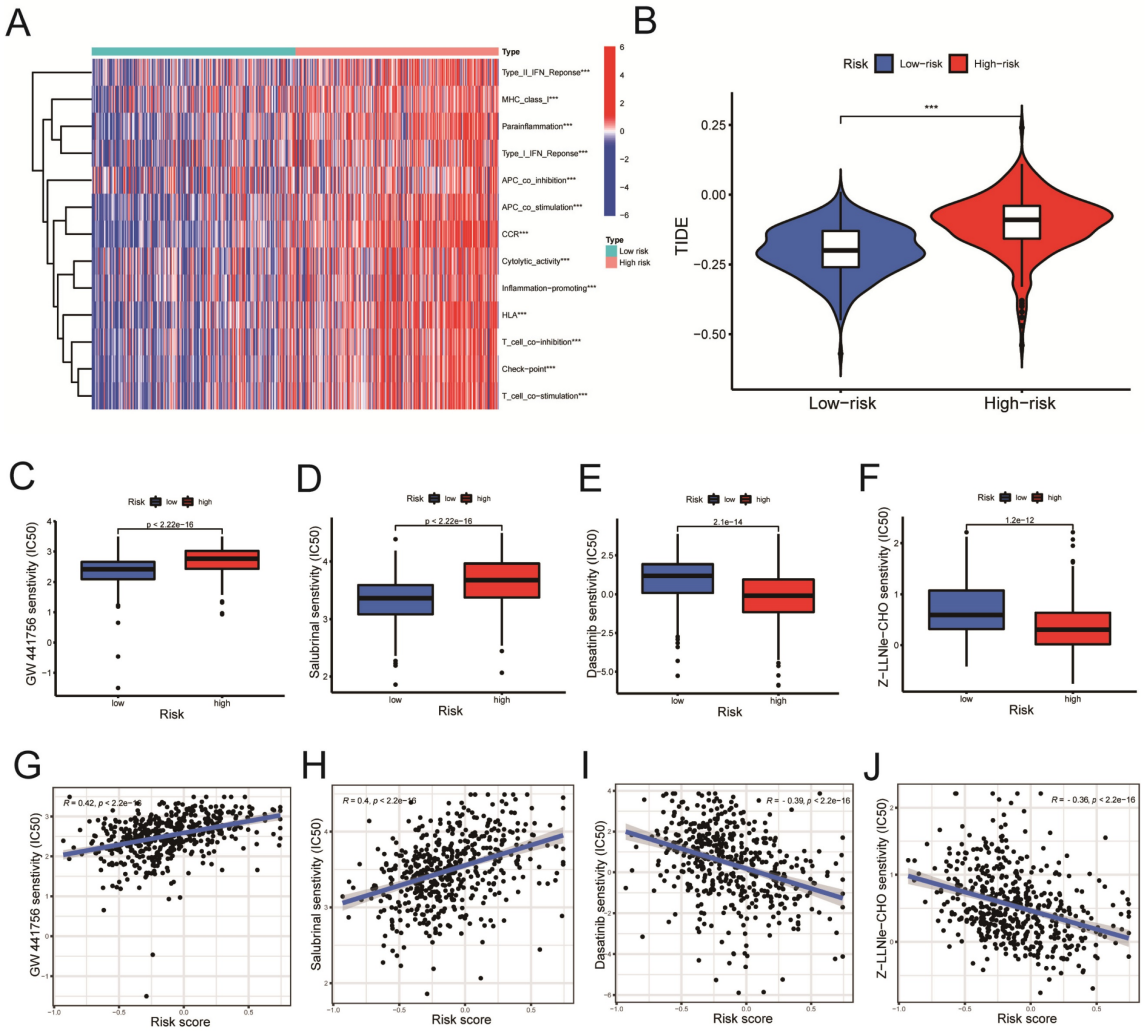
Immune-related functional analysis and drug sensitivity analysis. A. Immune-related functional analyses were displayed on heat maps. B. Tumor Immune Dysfunction and Exclusion (TIDE) analyses in high-risk and low-risk groups. C-F. Drug sensitivity analyses in (C-D) low- and (E-F) high-risk groups. G-J. Correlation analyses between drug IC50 value and risk score. Statistics were considered significant when the * p-value was less than 0.05, ** p-value less than 0.01, and *** p-value less than 0.001.

According to the ssGSEA algorithm, the enrichment score of the high-risk group is higher than that of the low-risk group in several immune function pathways, including the IFN-γ, the MHC I pathway, and others (Figure 8A, P < 0.001). The risk of tumor immune escape was calculated using the Tumor Immune Dysfunction and Exclusion (TIDE) algorithm. The result revealed that the high-risk group had a higher TIDE score than the low-risk group, indicating a higher probability of immune escape. (Figure 8B, P < 0.001). We further explored potentially effective therapeutic drugs for LUSC patients using the 'pRRophetic' R package (Table 4).

The GW441756 (Figure 8C) and Salubrinal (Figure 8D) had a lower inhibitory concentration-50 (IC50) value in the low-Risk Score group (P < 0.001) (Figure 8C, D). On the other hand, Dasatinib (Figure 8E) and Z-LLNIe-CHO (Figure 8F) drugs had a lower IC50 value in the high-Risk Score group (P < 0.001) (Figure 8E, F). As shown in figure 8G-H, the risk scores were positively correlated with the IC50 values of the two pharmaceuticals, GW441756 and Salubrinal (Figure 8G, R = 0.42; Figure 8H, R = 0.4. P < 0.001), indicating that patients with low-risk ratings were likely to respond to these medications more effectively. Meanwhile, Figure 8I-J revealed the IC50 of Dasatinib (R =-0.39) and Z-LLNIe-CHO (R = -0.36) were negatively connected with Risk Scores (Figure 8I, J, P < 0.001), suggesting that LUSC patients with higher risk scores are more responsive to dasatinib and Z-LLNIe CHO (Figure 8I, R = -0.39; Figure 8J, R = -0.36).

### LUSC cuproptosis cell model qRT-PCR detection

**Figure 9:**
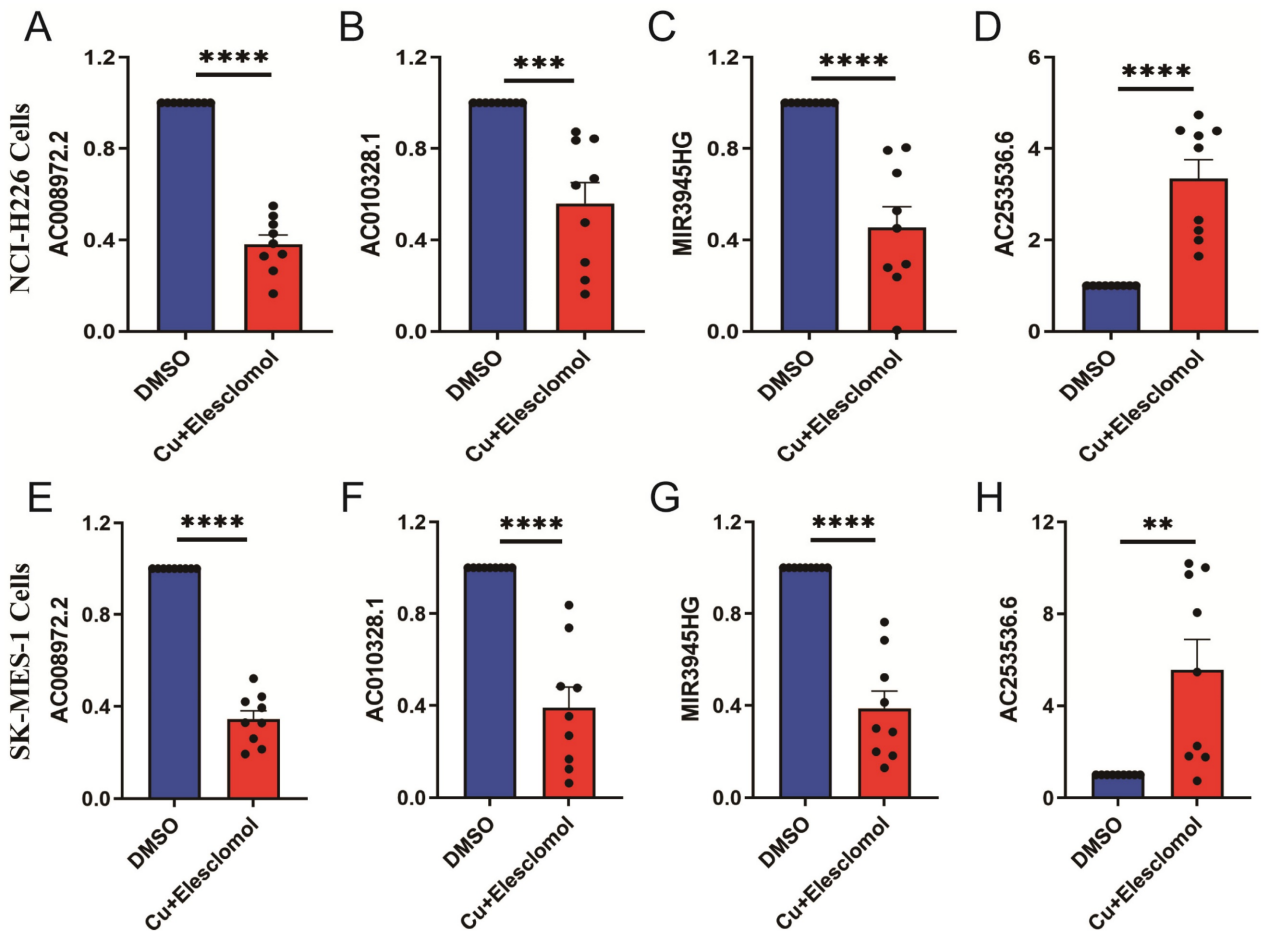
The expression levels of 5-cuproptosis-related lncRNAs in NCI-H226 and SK-MES-1 cells. After treated with 30 nM elesclomol and Cucl_2_ (1:1) mixture, the expression levels of AC008972.2 (A), AC010328.1 (B), MIR3945HG (C), and AC253536.6 (D) were detected by quantitative Real-time PCR in NCI-H226 cells. Similarly, the expression levels of AC008972.2 (E), AC010328.1 (F), MIR3945HG (G), and AC253536.6 (H) were detected by quantitative Real-time PCR in SK-MES-1 cells. Statistics were considered significant when the * p-value was less than 0.05, ** p-value less than 0.01, *** p-value less than 0.001 and **** p-value less than 0.0001.

The cells treated with 30 nM elesclomol and Cucl_2_ (1:1) mixture were collected to detected the expressions of LncRNAs using qRT-PCR. AC253536.6 expression was considerably higher in NCI-H226 cells, while AC008972.2, AC010328.1, and MIR3945HG expression was significantly lower (Figure 9A-D). Similarly, the SK-MES-1 cell type had the same results (Figure 9E-H). AC002467.1 expression was not detected in either of the two cell types, which could be attributed to the cells' low expression levels.

## Discussion

Lung cancer has the second-highest incidence rate yet has the most remarkable fatality rate of all malignancies 19. Lung cancer patients typically acquire medication resistance and are vulnerable to tumor metastasis at an early stage, which makes the clinical outcome of lung cancer treatment less than ideal 20. Lung squamous cell carcinoma, which makes up around 40% of all lung malignancies, and lung adenocarcinoma are examples of the subtype of lung cancer known as non-small cell lung cancer 21, 22. Since lung squamous cell carcinoma lacks effective treatment 23, the predicted survival rate of patients with lung squamous cell carcinoma does not reach 5% 24. Therefore, exploring new therapeutic targets and molecular mechanisms of lung squamous cell carcinoma is still challenging.

Copper-induced cell death, also called cuproptosis, is a novel form of death that is gaining the interest of more and more scientists. According to studies, copper ions can currently target the tricarboxylic acid cycle pathway after entering the cell, leading to the aggregation of lipoylated proteins (DBT, DLAT, GCSH, and DLST), instability of the Fe-S cluster proteins (FDX1, LIAS, ACO-2, SDHB, POLD1, and DPYD), and oligomerization of DLAT 8. It has been discovered that malignant tumors have much higher copper content 11. According to animal studies, chelating medicines that include copper ions can slow the growth of various cancers 25, 26. So far, the molecular mechanism of copper death causing cancer has rarely been reported. Based on bioinformatics, many researchers have predicted the genetic markers of copper death in tumors 20, 27. Yang et al. confirmed the increased expression of copper death-related genes (LIPT1, DLD, PDHA1, DLAT, FDX1, CDKN2A) in osteosarcoma 28. Zhang et al. additionally foresaw the crucial function of the copper death critical gene FDX1 in hepatocellular cancer 29.

Long non-coding RNA (LncRNA) is a long-chain non-coding RNA with a length of more than 200 nucleotides, which is an essential part of the non-coding genome. They Influence cell function through different patterns 30. Researchers found that LncRNAs play an indispensable role in various cancers, such as breast cancer, non-small cell lung cancer, etc. 31, 32. Chen et al. discovered that the miR-877-3p/FGF2 axis controls cervical cancer migration and invasion through the LncRNA HOXD-AS1^33^. Lin et al. showed that LncRNA FGD5-AS1 could bind miR-520a-3p to target KIAA1522 and affect the proliferation of lung adenocarcinoma cells 34. Wang et al. developed a LUAD predictive signature in the most recent study that was published by thoroughly examining the TCGA and GEO databases 35.

Our study thoroughly analyzed the TCGA database's cuproptosis-related mRNA expression profiling of LUSC patients. Pearson correlation analysis revealed 291 LncRNA that had significant relationships with 19 cuproptosis-related genes (Figure 2A, P < 0.001). Of the 291 cuproptosis-related LncRNAs, 23 cuproptosis-related LncRNAs (P < 0.05) as LUSC independent prognostic factors were identified by univariate Cox regression analysis (Figure 2B, P < 0.05). Finally, multivariate Cox proportional hazards regression analysis identified five cuproptosis-related LncRNAs (MIR3945HG, AC002467.1, AC008972.2, LncRNA AC010328.1, and AC253536.6) that were used to build a prognostic model (Figure 2E). According to Chen et al., high-expressed MIR3945HG levels were strongly connected with shorter survival of patients with LUSC 36. Wu et al. discovered that the LncRNA AC002467.1, which was found to be abnormally expressed in ovarian cancer, had a negative relationship with the survival of ovarian cancer patients 37. AC008972.2 has been linked to m7G RNA methylation in colon cancer, according to Liu et al. 38. However, the role of LncRNA AC010328.1 and AC253536.6 in diseases has rarely been reported.

Four hundred ninety-three patients with LUSC were grouped into low-risk or high-risk groups based on the median Risk Score acquired from the risk model we built. The Kaplan-Meier survival analysis revealed that the survival time for patients in the high-risk Score group was poorer than those in the low-Risk Score group (Figure 3A, P < 0.001). Next, all LUSC patients were divided into three groups according to clinical stages (clinical stage I-II, II-III o low- and high-Risk Score subgroups. For clinical stage I-II patients, the overall survival of III-IV and each group was subgrouped in the high-Risk Score subgroup was lower than that of the low-Risk Score subgroup both for clinical stage I-II patients and II-III patients (Figure 3D, P < 0.001; 3E, P = 0.023). However, because only a few patients were enrolled in clinical stages III-IV, there was no statistically significant difference in overall survival between the high and low-Risk Score subgroups. (Figure 3F, P = 0.689). Additionally, an examination of five LncRNA profiles with mRNA correlations (Figure 3J) reveals LncRNA AC002467.1 was highly negatively correlated with NLRP3 (P < 0.001) and positively correlated with cuprotosis-related mRNA DLD (P < 0.001). In contrast, LncRNA AC008972.2 positively correlated with NLRP3 (P < 0.001). NLRP3 is an inflammatory body activated during mitochondrial dysfunction, resulting in alcoholic and nonalcoholic hepatitis 39. The copper transporter ATP7B and the LncRNA AC010328.1 positively correlated (P < 0.001). ATP7B interacted with the autophagy marker LC3 to induce autophagy, and ATP7B on the autophagic membrane promoted Cu ion transfer into autophagosome 40. MIR3945HG was found to be negatively correlated with several-copper death-related genes (NFE2L2, LIPT1, GCSH) (P < 0.001). LIPT1 has been found to promote hepatocellular carcinoma proliferation, invasion, and migration 41. Since LncRNAs regulate gene expression through various complex pathways, the mechanism of the above five LncRNAs affecting 19-copper-death-related genes needs to be investigated further.

All of the LUSC samples were randomly divided into training (40%) and testing (60%) sets to verify the credibility of our risk model. The training and testing sets were divided into high-risk and low-risk groups based on the median risk score. For the training set, LUSC patients in the high-risk group had significantly shorter overall survival compared with those in the low-risk group (P = 0.003) (Figure 4C). Moreover, the training set's outcomes were similar to those of the testing set (P = 0.013) (Figure 4G). Following that, univariate and multivariate COX regression analysis revealed that clinical stage and risk score are independent prognostic indicators for LUSC patients (Figure 5A, B, P < 0.05). More importantly, risk scores outperform the clinical stage in terms of predictive power (Figure 5C). Following that, we created a nomogram to predict the prognosis of LUSC patients (Figure 6A, B). Each patient's survival rate of 1/3/5 year can be predicted based on age, gender, clinical stage, and Risk Score (See Figure 6A). Our nanogram will be a valuable tool for clinicians in predicting the prognosis of patients with LUSC.

The enrichment analysis of differentially expressed genes in high and low-risk groups reveals that the majority of differentially expressed genes are enriched in immune-related GO terms like leukocyte-mediated immune response, humoral immune response, B cell-mediated immune response, and other immune biological processes (Figure 7A). Recently, Zhang et al. discovered that circRNA (circHMGB2) overexpression reshaped the tumor microenvironment and regulated anti-PD-1 resistance in patients with lung squamous cell carcinoma, thereby promoting the progression of LUSC 1. According to KEGG enrichment analysis, these mRNAs were enriched in the phagosome (hsa04145) and cell adhesion molecule (hsa04514) pathways (Figure 7B). Su et al. discovered that AIM2, a novel inflammasome, was recruited into phagosomes and then up-regulated PD-L1 to cause immunosuppression 42. Adhesion molecule expression inhibited T cell infiltration into tumors, posing a challenge to cancer immunotherapy 43. We discovered no discernible difference in the tumor mutational burden (TMB) between the high-risk and low-risk groups (Figure 7E), implying that mutations in the differentially expressed mRNAs had little impact on the prognosis of LUAD patients.

Notably, we used the "pRRophetic" R package to investigate potentially effective therapeutic drugs for LUSC patients (Table 4). The drug sensitivity analysis showed that patients with low-risk ratings were likely to respond to GW441756 and Salubrinal. In contrast, patients with higher risk scores are more responsive to Dasatinib and Z-LLNIe CHO. Dasatinib is currently used to treat chronic myeloid leukemia (CML), but it has been linked to pulmonary pleural effusion (PE) and pulmonary hypertension (PAH) 44. Interestingly, researchers discovered that Dasatinib inhibited the activity of the MCM7 protein, making it a promising cancer therapy 45. The proteasome inhibitor MG-132 has a similar structure to secretase inhibitor I (GSI-I; Z-LLNIe CHO) and can inhibit the proteasome, which is a crucial cancer treatment candidate 46.

In conclusion, we developed a predictive risk model based on five copper death-related LncRNAs for LUSC patients. To forecast the prognosis of LUSC patients, we constructed a nomogram. Additionally, the drug sensitivity analysis revealed several candidates responsive to LUSC patients at high or low risk.

## Supplementary Material

Supplementary tables.

## Figures and Tables

**Table 1:** Primer set for qRT-PCR utilized in copper dead cell models.

lncRNA	Forward primer (5'-3')	Reverse primer (5'-3')
AC008972.2	AGTGGAAAGAATAAGGAGCAGCC	CCTGCATATTCTGACCCTAAAACT
AC010328.1	GGCATTTTGCAGGCACACCT	GCTCAATGCTGGACACCCA
MIR3945HG	CTTTCCTCTTCCGACATTTGCTG	CGTCTTGTGACTGAGAGGTGG
AC253536.6	TAGGAGGACGGACACCTCGG	CCTTAACCCACAATGGCGCT
AC002467.1	CTCCAGAAAATGGCCTGGGAA	TGCTTTGGGAATCACCTTGAAAC
β-actin	AGTTGC GTTACACCCTTTC	CCTTCACCGTTCCAGTTT

**Table 2:** The selection bias between high-risk and low-risk groups before and after PSM analysis.

	Before PSM					After PSM			
Variable	low-risk	high-risk	*X* ^2^	*P*^e^ value		low-risk	high-risk	*X* ^2^	*P*^e^ value
**Age(years)**									
Mean^a^ (SD^b^)	66.8(8.44)	67.6(8.56)	1.124	0.262		68.3(6.92)	68.5(6.94)	0.303	0.762
Median (Min^c^, Max^d^)	69.0(41.0, 85.0)	69.0(41.0, 85.0)				69.5(45.0, 84.0)	69.0(45.0, 84.0)		
**Gender**									
Male	193(78.8%)	164(68.6%)	5.934	0.0149		137(80.6%)	125(77.2%)	0.398	0.528
Female	52(21.2%)	75(31.4%)				33(19.4%)	37(22.8%)		
**Stage**									
Mean^a^ (SD)	1.68(0.75)	1.74(0.85)	0.753	0.452		1.59(0.718)	1.54(0.732)	-0.643	0.521
Median (Min^c^, Max^d^)	2.00(1.00, 4.00)	1.00(1.00, 4.00)				1.00(1.00, 3.00)	1.00(1.00, 3.00)		

a. average value. b. standard deviation. c. minimum value. d. maximum value. e. the *P* value of the chi-square test.

**Table 3:** Patients with lung squamous cell carcinoma (n=493) in TCGA database randomly divided into train group and test group.

	Numbers of patients			
	Total (493)	Test (295)	Train (198)	*P* value^b^
**Age(years)**				
≤65	189	116	73	0.701
>65	299	177	122	
Unknown^a^	5	2	3	
**Gender**				
Female	128	77	51	1
Male	365	218	147	
**Stage**				
I	241	139	102	0.717
II	158	100	58	
III	83	51	32	
IV	7	4	3	
Unknown^a^	4	1	3	
**T stage**				
1	114	70	44	0.791
2	286	166	120	
3	70	44	26	
4	23	15	8	
**N stage**				
0	316	184	132	0.552
1	127	81	46	
2	40	25	15	
3	5	2	3	
Unknown^a^	5	3	2	
**M stage**				
0	405	244	161	1
1	7	4	3	
Unknown^a^	81	47	34	

a. data on such clinical features is missing from the TCGA database. b. the P value of the chi-square test.

**Table 4:** Potential drug sensitivity and correlation analysis.

	Differential analysis		Correlation analysis	
Drug	*P* value^a^		R	cor *P* value^b^
Dasatinib	2.1e-14		-0.39	<2.2e-16
Z-LLNle-CHO	1.2e-12		-0.36	<2.2e-16
DMOG	1.3e-12		-0.36	<2.2e-16
Sunitinib	1.9e-12		-0.37	<2.2e-16
THZ-2-49	3.2e-12		-0.38	<2.2e-16
GW 441756	<2.22e-16		0.42	<2.2e-16
Salubrinal	<2.22e-16		0.40	<2.2e-16
Vinorelbine	3.1e-16		0.41	<2.2e-16
YK 4-279	5.1e-16		0.39	<2.2e-16
LAQ824	1.1e-15		0.36	<2.2e-16
Cytarabine	1.3e-15		0.40	<2.2e-16
rTRAIL	4.2e-14		0.38	<2.2e-16
FH535	5.9e-14		0.35	<1.6e-15
Pyrimethamine	1.7e-13		0.37	<2.2e-16
Vorinostat	1.1e-12		0.33	<7.1e-14
PD-0332991	1.3e-12		0.34	<2.1e-14
OSU-03012	1.3e-12		0.33	<3.4e-14

a. the P value of the wilcox test. b. the P value of the spearman analysis.
