# Supplementary material for: A five-cuproptosis-related LncRNA Signature: predicting prognosis, assessing immune function & drug sensitivity in lung squamous cell carcinoma
Source: J Cancer. 2023 May 21;14(9):1499–514. doi: 10.7150/jca.82370 (PMC10266248; doi:10.7150/jca.82370)
Supplement: Supplementary file 1 — Supplementary tables. [file jcav14p1499s1.pdf]

**supplementary table 1**

| cuproptosis-related gene |
|---|
| NFE2L2 |
| NLRP3 |
| ATP7B |
| ATP7A |
| SLC31A1 |
| FDX1 |
| LIAS |
| LIPT1 |
| LIPT2 |
| DLD |
| DLAT |
| PDHA1 |
| PDHB |
| MTF1 |
| GLS |
| CDKN2A |
| DBT |
| GCSH |
| DLST |

**supplementary table 2**

| id | age | diagnosis | M | N | T | gender | Status | primary diagnosis.diagnoses | stage |
|---|---|---|---|---|---|---|---|---|---|
| TCGA-18-3406-01A | 67 | Lung Squamous Cell Carcinoma | M0 | N0 | T1 | male | Dead | Squamous cell carcinoma, NOS | stage ia |
| TCGA-18-3407-01A | 72 | Lung Squamous Cell Carcinoma | M0 | N0 | T2 | male | Dead | Squamous cell carcinoma, NOS | stage ib |
| TCGA-18-3408-01A | 77 | Lung Squamous Cell Carcinoma | M0 | N0 | T2 | female | Dead | Squamous cell carcinoma, NOS | stage ib |
| TCGA-18-3409-01A | 74 | Lung Squamous Cell Carcinoma | M0 | N0 | T1 | male | Alive | Basaloid squamous cell carcinoma | stage ia |
| TCGA-18-3410-01A | 81 | Lung Squamous Cell Carcinoma | M0 | N0 | T3 | male | Dead | Squamous cell carcinoma, NOS | stage iib |
| TCGA-18-3411-01A | 63 | Lung Squamous Cell Carcinoma | M0 | N2 | T2 | female | Alive | Squamous cell carcinoma, NOS | stage iiia |
| TCGA-18-3412-01A | 52 | Lung Squamous Cell Carcinoma | M0 | N0 | T2 | male | Dead | Squamous cell carcinoma, NOS | stage ib |
| TCGA-18-3414-01A | 73 | Lung Squamous Cell Carcinoma | M1 | N1 | T4 | male | Dead | Squamous cell carcinoma, NOS | stage iv |
| TCGA-18-3415-01A | 77 | Lung Squamous Cell Carcinoma | M0 | N0 | T2 | male | Dead | Squamous cell carcinoma, NOS | stage ib |
| TCGA-18-3416-01A | 83 | Lung Squamous Cell Carcinoma | M0 | N1 | T2 | male | Dead | Squamous cell carcinoma, NOS | stage iib |
| TCGA-18-3417-01A | 65 | Lung Squamous Cell Carcinoma | M1 | N1 | T2 | male | Dead | Squamous cell carcinoma, NOS | stage iv |
| TCGA-18-3419-01A | 73 | Lung Squamous Cell Carcinoma | M0 | N1 | T2 | male | Alive | Squamous cell carcinoma, NOS | stage iib |
| TCGA-18-3421-01A | 65 | Lung Squamous Cell Carcinoma | M0 | N0 | T2 | male | Alive | Squamous cell carcinoma, NOS | stage ib |
| TCGA-18-4083-01A | 63 | Lung Squamous Cell Carcinoma | M0 | N1 | T2 | male | Dead | Squamous cell carcinoma, NOS | stage iib |
| TCGA-18-4086-01A | 64 | Lung Squamous Cell Carcinoma | M0 | N0 | T2 | male | Dead | Squamous cell carcinoma, NOS | stage ib |
| TCGA-18-4721-01A | 74 | Lung Squamous Cell Carcinoma | M0 | N0 | T1 | male | Alive | Squamous cell carcinoma, NOS | stage ia |
| TCGA-18-5592-01A | 57 | Lung Squamous Cell Carcinoma | M0 | N0 | T3 | male | Alive | Squamous cell carcinoma, NOS | stage iib |
| TCGA-18-5595-01A | 50 | Lung Squamous Cell Carcinoma | M0 | N0 | T2 | male | Dead | Squamous cell carcinoma, NOS | stage ib |
| TCGA-21-1070-01A | 60 | Lung Squamous Cell Carcinoma | M0 | N0 | T3 | female | Alive | Squamous cell carcinoma, NOS | stage iiia |
| TCGA-21-1071-01A | 67 | Lung Squamous Cell Carcinoma | M0 | N0 | T2 | male | Dead | Squamous cell carcinoma, NOS | stage ib |
| TCGA-21-1072-01A | 75 | Lung Squamous Cell Carcinoma | M0 | N0 | T2 | male | Alive | Squamous cell carcinoma, NOS | stage ib |
| TCGA-21-1075-01A | 57 | Lung Squamous Cell Carcinoma | M0 | N1 | T2 | male | Alive | Squamous cell carcinoma, NOS | stage iib |

| | | | | | | | | | |
|---|---|---|---|---|---|---|---|---|---|
| TCGA-21-1076-01A | 54 | Lung Squamous Cell Carcinoma | M0 | N0 | T2 | female | Alive | Squamous cell carcinoma, NOS | stage ib |
| TCGA-21-1077-01A | 64 | Lung Squamous Cell Carcinoma | M0 | N1 | T2 | male | Dead | Squamous cell carcinoma, NOS | stage iib |
| TCGA-21-1078-01A | 77 | Lung Squamous Cell Carcinoma | M0 | N0 | T2 | male | Dead | Squamous cell carcinoma, NOS | stage ib |
| TCGA-21-1079-01A | 71 | Lung Squamous Cell Carcinoma | M0 | N0 | T3 | male | Dead | Squamous cell carcinoma, NOS | stage iiia |
| TCGA-21-1080-01A | 66 | Lung Squamous Cell Carcinoma | M0 | N0 | T2 | male | Alive | Squamous cell carcinoma, NOS | stage ib |
| TCGA-21-1081-01A | 69 | Lung Squamous Cell Carcinoma | M0 | N1 | T2 | male | Dead | Squamous cell carcinoma, NOS | stage iib |
| TCGA-21-1082-01A | 61 | Lung Squamous Cell Carcinoma | M0 | N0 | T2 | male | Alive | Squamous cell carcinoma, NOS | stage ib |
| TCGA-21-1083-01A | 75 | Lung Squamous Cell Carcinoma | M0 | N0 | T1 | male | Dead | Squamous cell carcinoma, NOS | stage ia |
| TCGA-21-5782-01A | 68 | Lung Squamous Cell Carcinoma | M0 | N0 | T2 | female | Dead | Squamous cell carcinoma, NOS | stage ib |
| TCGA-21-5783-01A | 76 | Lung Squamous Cell Carcinoma | M0 | N0 | T2 | male | Dead | Squamous cell carcinoma, NOS | stage ib |
| TCGA-21-5784-01A | 80 | Lung Squamous Cell Carcinoma | M0 | N0 | T2 | female | Alive | Squamous cell carcinoma, NOS | stage ib |
| TCGA-21-5786-01A | 64 | Lung Squamous Cell Carcinoma | M0 | N0 | T2 | male | Alive | Squamous cell carcinoma, NOS | stage ib |
| TCGA-21-5787-01A | 65 | Lung Squamous Cell Carcinoma | M0 | N2 | T2 | male | Dead | Squamous cell carcinoma, NOS | stage iiia |
| TCGA-21-A5DI-01A | 77 | Lung Squamous Cell Carcinoma | M0 | N0 | T1b | male | Alive | Squamous cell carcinoma, NOS | stage ia |
| TCGA-22-0940-01A | 71 | Lung Squamous Cell Carcinoma | M0 | N1 | T1 | male | Dead | Squamous cell carcinoma, NOS | stage iia |
| TCGA-22-0944-01A | 61 | Lung Squamous Cell Carcinoma | M0 | N0 | T2 | male | Dead | Squamous cell carcinoma, NOS | stage ib |
| TCGA-22-1000-01A | 76 | Lung Squamous Cell Carcinoma | M0 | N0 | T2 | male | Dead | Squamous cell carcinoma, NOS | stage ib |
| TCGA-22-1002-01A | 69 | Lung Squamous Cell Carcinoma | M0 | N0 | T1 | male | Dead | Squamous cell carcinoma, NOS | stage ia |
| TCGA-22-1005-01A | 63 | Lung Squamous Cell Carcinoma | M0 | N0 | T1 | male | Dead | Squamous cell carcinoma, NOS | stage ia |
| TCGA-22-1011-01A | 73 | Lung Squamous Cell Carcinoma | M0 | N0 | T2 | male | Dead | Squamous cell carcinoma, NOS | stage ib |
| TCGA-22-1012-01A | 80 | Lung Squamous Cell Carcinoma | M0 | N0 | T2 | female | Dead | Squamous cell carcinoma, NOS | stage ib |
| TCGA-22-1016-01A | 65 | Lung Squamous Cell Carcinoma | M0 | N0 | T2 | male | Dead | Squamous cell carcinoma, NOS | stage ib |
| TCGA-22-1017-01A | 62 | Lung Squamous Cell Carcinoma | M0 | N0 | T1 | male | Dead | Squamous cell carcinoma, NOS | stage ia |
| TCGA-22-4591-01A | 80 | Lung Squamous Cell Carcinoma | M0 | N2 | T3 | male | Dead | Squamous cell carcinoma, NOS | stage iiia |
| TCGA-22-4593-01A | 77 | Lung Squamous Cell Carcinoma | M0 | N0 | T2b | male | Dead | Squamous cell carcinoma, NOS | stage iia |

| | | | | | | | | | |
|---|---|---|---|---|---|---|---|---|---|
| TCGA-22-4594-01A | 60 | Lung Squamous Cell Carcinoma | M0 | N2 | T3 | female | Dead | Squamous cell carcinoma, NOS | stage iiia |
| TCGA-22-4595-01A | 57 | Lung Squamous Cell Carcinoma | | N2 | T3 | male | Dead | Squamous cell carcinoma, NOS | stage iiia |
| TCGA-22-4596-01A | 69 | Lung Squamous Cell Carcinoma | M0 | N0 | T1b | female | Dead | Squamous cell carcinoma, NOS | stage ib |
| TCGA-22-4599-01A | 73 | Lung Squamous Cell Carcinoma | M0 | N0 | T2a | female | Dead | Squamous cell carcinoma, NOS | stage ib |
| TCGA-22-4601-01A | 73 | Lung Squamous Cell Carcinoma | M0 | N0 | T4 | female | Dead | Squamous cell carcinoma, NOS | stage iiia |
| TCGA-22-4604-01A | 73 | Lung Squamous Cell Carcinoma | M0 | N1 | T2a | male | Dead | Squamous cell carcinoma, NOS | stage iia |
| TCGA-22-4605-01A | 78 | Lung Squamous Cell Carcinoma | M0 | N0 | T2 | female | Dead | Squamous cell carcinoma, large cell, nonkeratinizing, NOS | stage ib |
| TCGA-22-4607-01A | 75 | Lung Squamous Cell Carcinoma | M0 | N0 | T2a | male | Dead | Squamous cell carcinoma, NOS | stage ib |
| TCGA-22-4609-01A | 81 | Lung Squamous Cell Carcinoma | M0 | N0 | T1 | male | Dead | Squamous cell carcinoma, NOS | stage ia |
| TCGA-22-4613-01A | 73 | Lung Squamous Cell Carcinoma | M0 | N0 | T1b | female | Dead | Squamous cell carcinoma, NOS | stage ia |
| TCGA-22-5471-01A | 75 | Lung Squamous Cell Carcinoma | M0 | N0 | T2 | male | Alive | Squamous cell carcinoma, NOS | stage ib |
| TCGA-22-5472-01A | 67 | Lung Squamous Cell Carcinoma | M0 | N0 | T2a | male | Dead | Squamous cell carcinoma, NOS | stage ib |
| TCGA-22-5473-01A | 78 | Lung Squamous Cell Carcinoma | M0 | N0 | T3 | male | Dead | Squamous cell carcinoma, NOS | not reported |
| TCGA-22-5474-01A | 74 | Lung Squamous Cell Carcinoma | M0 | N0 | T2a | male | Dead | Squamous cell carcinoma, NOS | stage ib |
| TCGA-22-5477-01A | 65 | Lung Squamous Cell Carcinoma | M0 | N0 | T1 | male | Dead | Squamous cell carcinoma, NOS | stage ia |
| TCGA-22-5478-01A | 79 | Lung Squamous Cell Carcinoma | M0 | N0 | T2a | male | Dead | Squamous cell carcinoma, NOS | stage ib |
| TCGA-22-5479-01A | 64 | Lung Squamous Cell Carcinoma | M0 | N0 | T2 | male | Dead | Squamous cell carcinoma, NOS | stage ib |
| TCGA-22-5480-01A | 66 | Lung Squamous Cell Carcinoma | M0 | N0 | T1b | female | Dead | Squamous cell carcinoma, NOS | stage ia |
| TCGA-22-5481-01A | 72 | Lung Squamous Cell Carcinoma | M0 | N1 | T2 | female | Dead | Squamous cell carcinoma, NOS | stage iib |
| TCGA-22-5482-01A | 81 | Lung Squamous Cell Carcinoma | M0 | N0 | T2a | male | Dead | Squamous cell carcinoma, NOS | stage ib |
| TCGA-22-5483-01A | 74 | Lung Squamous Cell Carcinoma | M0 | N1 | T1a | male | Dead | Squamous cell carcinoma, NOS | stage iia |
| TCGA-22-5485-01A | 58 | Lung Squamous Cell Carcinoma | M0 | N0 | T1a | female | Dead | Squamous cell carcinoma, NOS | stage ia |
| TCGA-22-5489-01A | 64 | Lung Squamous Cell Carcinoma | M0 | N0 | T1b | male | Dead | Squamous cell carcinoma, NOS | stage ia |

| | | | | | | | | | |
|---|---|---|---|---|---|---|---|---|---|
| TCGA-22-5491-01A | 74 | Lung Squamous Cell Carcinoma | M0 | N0 | T1a | male | Dead | Squamous cell carcinoma, NOS | stage ia |
| TCGA-22-5492-01A | 73 | Lung Squamous Cell Carcinoma | M0 | N2 | T2a | female | Dead | Squamous cell carcinoma, NOS | stage iiia |
| TCGA-22-A5C4-01A | 70 | Lung Squamous Cell Carcinoma | M0 | N0 | T2b | male | Alive | Squamous cell carcinoma, NOS | stage iia |
| TCGA-33-4532-01A | 68 | Lung Squamous Cell Carcinoma | M0 | N0 | T2 | male | Dead | Squamous cell carcinoma, NOS | stage ib |
| TCGA-33-4533-01A | 76 | Lung Squamous Cell Carcinoma | M0 | N0 | T2 | female | Alive | Squamous cell carcinoma, NOS | stage ib |
| TCGA-33-4538-01A | 66 | Lung Squamous Cell Carcinoma | M0 | N2 | T2 | male | Dead | Squamous cell carcinoma, NOS | stage iiia |
| TCGA-33-4547-01A | 68 | Lung Squamous Cell Carcinoma | M0 | N0 | T2 | male | Alive | Squamous cell carcinoma, NOS | stage ib |
| TCGA-33-4566-01A | 40 | Lung Squamous Cell Carcinoma | M0 | N0 | T2 | male | Dead | Squamous cell carcinoma, NOS | stage ib |
| TCGA-33-4582-01A | 55 | Lung Squamous Cell Carcinoma | M0 | N0 | T1 | male | Dead | Squamous cell carcinoma, NOS | stage ia |
| TCGA-33-4583-01A | 73 | Lung Squamous Cell Carcinoma | M0 | N0 | T1 | male | Dead | Squamous cell carcinoma, NOS | stage ia |
| TCGA-33-4586-01A | 57 | Lung Squamous Cell Carcinoma | M0 | N2 | T2 | male | Dead | Squamous cell carcinoma, NOS | stage iiia |
| TCGA-33-4587-01A | 63 | Lung Squamous Cell Carcinoma | MX | N0 | T2 | female | Dead | Squamous cell carcinoma, small cell, nonkeratinizing | stage ib |
| TCGA-33-4589-01A | 62 | Lung Squamous Cell Carcinoma | M0 | N1 | T2 | female | Dead | Squamous cell carcinoma, NOS | stage iib |
| TCGA-33-6737-01A | 71 | Lung Squamous Cell Carcinoma | M0 | N2 | T2 | male | Dead | Squamous cell carcinoma, NOS | stage iiia |
| TCGA-33-6738-01A | 80 | Lung Squamous Cell Carcinoma | MX | N2 | T1 | male | Alive | Squamous cell carcinoma, NOS | stage iiia |
| TCGA-33-A4WN-01A | 60 | Lung Squamous Cell Carcinoma | MX | N0 | T2a | male | Dead | Squamous cell carcinoma, NOS | stage ib |
| TCGA-33-A5GW-01A | 67 | Lung Squamous Cell Carcinoma | MX | N1 | T1a | male | Dead | Squamous cell carcinoma, NOS | stage iia |
| TCGA-33-AAS8-01A | 59 | Lung Squamous Cell Carcinoma | MX | N0 | T1 | female | Dead | Squamous cell carcinoma, NOS | stage ia |
| TCGA-33-AASB-01A | 66 | Lung Squamous Cell Carcinoma | MX | N0 | T2 | male | Dead | Squamous cell carcinoma, NOS | stage ib |
| TCGA-33-AASD-01A | 83 | Lung Squamous Cell Carcinoma | MX | N0 | T1 | male | Dead | Squamous cell carcinoma, NOS | stage ia |
| TCGA-33-AASI-01A | 65 | Lung Squamous Cell Carcinoma | MX | N1 | T2 | female | Dead | Squamous cell carcinoma, NOS | stage iib |
| TCGA-33-AASJ-01A | 60 | Lung Squamous Cell Carcinoma | MX | N0 | T2 | male | Dead | Squamous cell carcinoma, NOS | stage ib |
| TCGA-33-AASL-01A | 57 | Lung Squamous Cell Carcinoma | MX | N0 | T1 | female | Dead | Squamous cell carcinoma, NOS | stage ia |
| TCGA-34-2596-01A | 70 | Lung Squamous Cell Carcinoma | M0 | N1 | T2 | male | Dead | Squamous cell carcinoma, NOS | stage iib |

| | | | | | | | | | |
|---|---|---|---|---|---|---|---|---|---|
| TCGA-34-2600-01A | 76 | Lung Squamous Cell Carcinoma | M0 | N0 | T1 | female | Dead | Squamous cell carcinoma, NOS | stage ia |
| TCGA-34-2608-01A | 84 | Lung Squamous Cell Carcinoma | M0 | N0 | T2 | male | Dead | Squamous cell carcinoma, NOS | stage ib |
| TCGA-34-5231-01A | 72 | Lung Squamous Cell Carcinoma | M0 | N0 | T1 | male | Dead | Squamous cell carcinoma, NOS | stage ia |
| TCGA-34-5232-01A | 75 | Lung Squamous Cell Carcinoma | M0 | N1 | T1 | female | Alive | Squamous cell carcinoma, NOS | stage iia |
| TCGA-34-5234-01A | 71 | Lung Squamous Cell Carcinoma | M0 | N0 | T1 | female | Alive | Squamous cell carcinoma, NOS | stage ia |
| TCGA-34-5236-01A | 60 | Lung Squamous Cell Carcinoma | M0 | N0 | T3 | male | Dead | Squamous cell carcinoma, NOS | stage iib |
| TCGA-34-5239-01A | 75 | Lung Squamous Cell Carcinoma | M0 | N0 | T4 | male | Alive | Squamous cell carcinoma, NOS | stage iiia |
| TCGA-34-5240-01A | 73 | Lung Squamous Cell Carcinoma | M0 | N1 | T2 | female | Alive | Basaloid squamous cell carcinoma | stage iib |
| TCGA-34-5241-01A | 79 | Lung Squamous Cell Carcinoma | M0 | N0 | T2 | male | Dead | Squamous cell carcinoma, NOS | stage ib |
| TCGA-34-5927-01A | 70 | Lung Squamous Cell Carcinoma | M0 | N0 | T1 | female | Alive | Squamous cell carcinoma, NOS | stage ia |
| TCGA-34-5928-01A | 83 | Lung Squamous Cell Carcinoma | M0 | N1 | T2 | female | Alive | Squamous cell carcinoma, NOS | stage iib |
| TCGA-34-5929-01A | 78 | Lung Squamous Cell Carcinoma | M0 | N0 | T2 | female | Dead | Squamous cell carcinoma, NOS | stage ib |
| TCGA-34-7107-01A | 70 | Lung Squamous Cell Carcinoma | M0 | N0 | T2a | male | Dead | Squamous cell carcinoma, NOS | stage ii |
| TCGA-34-8454-01A | 62 | Lung Squamous Cell Carcinoma | M0 | N1 | T3 | female | Alive | Squamous cell carcinoma, NOS | stage iiia |
| TCGA-34-8455-01A | 67 | Lung Squamous Cell Carcinoma | M1a | N0 | T4 | male | Dead | Squamous cell carcinoma, NOS | stage iv |
| TCGA-34-8456-01A | 71 | Lung Squamous Cell Carcinoma | M0 | N1 | T2a | female | Alive | Squamous cell carcinoma, NOS | stage iia |
| TCGA-34-A5IX-01A | 80 | Lung Squamous Cell Carcinoma | M0 | N0 | T3 | male | Alive | Squamous cell carcinoma, NOS | stage iib |
| TCGA-37-3783-01A | 51 | Lung Squamous Cell Carcinoma | M0 | N2 | T3 | male | Alive | Squamous cell carcinoma, NOS | stage iiia |
| TCGA-37-3789-01A | 65 | Lung Squamous Cell Carcinoma | | N0 | T2 | male | Alive | Squamous cell carcinoma, NOS | stage ib |
| TCGA-37-3792-01A | 69 | Lung Squamous Cell Carcinoma | M0 | N0 | T2 | male | Alive | Squamous cell carcinoma, NOS | stage ib |
| TCGA-37-4129-01A | 52 | Lung Squamous Cell Carcinoma | M0 | N0 | T1b | female | Alive | Squamous cell carcinoma, NOS | stage ia |
| TCGA-37-4130-01A | 56 | Lung Squamous Cell Carcinoma | M0 | N0 | T1b | male | Alive | Squamous cell carcinoma, NOS | stage ia |
| TCGA-37-4132-01A | 61 | Lung Squamous Cell Carcinoma | M1 | N0 | T2 | female | Alive | Squamous cell carcinoma, NOS | stage iv |
| TCGA-37-4133-01A | 63 | Lung Squamous Cell Carcinoma | M0 | N0 | T4 | male | Alive | Squamous cell carcinoma, NOS | stage iiia |

| | | | | | | | | | |
|---|---|---|---|---|---|---|---|---|---|
| TCGA-37-4135-01A | 68 | Lung Squamous Cell Carcinoma | M0 | N0 | T2a | male | Alive | Squamous cell carcinoma, NOS | stage ib |
| TCGA-37-4141-01A | 65 | Lung Squamous Cell Carcinoma | M0 | N0 | T1b | female | Alive | Squamous cell carcinoma, NOS | stage ia |
| TCGA-37-5819-01A | 64 | Lung Squamous Cell Carcinoma | M0 | N2 | T2 | male | Alive | Squamous cell carcinoma, NOS | stage iiia |
| TCGA-37-A5EL-01A | 53 | Lung Squamous Cell Carcinoma | M0 | N0 | T3 | male | Dead | Squamous cell carcinoma, NOS | stage iib |
| TCGA-37-A5EM-01A | 49 | Lung Squamous Cell Carcinoma | M0 | N0 | T2 | male | Alive | Squamous cell carcinoma, NOS | stage ii |
| TCGA-37-A5EN-01A | 59 | Lung Squamous Cell Carcinoma | M0 | N2 | T4 | male | Alive | Squamous cell carcinoma, NOS | stage iiib |
| TCGA-39-5011-01A | 70 | Lung Squamous Cell Carcinoma | M0 | N0 | T1b | female | Alive | Squamous cell carcinoma, NOS | stage ia |
| TCGA-39-5016-01A | 44 | Lung Squamous Cell Carcinoma | M0 | N1 | T2a | male | Alive | Basaloid squamous cell carcinoma | stage iia |
| TCGA-39-5019-01A | 70 | Lung Squamous Cell Carcinoma | M0 | N0 | T2a | male | Alive | Squamous cell carcinoma, NOS | stage ib |
| TCGA-39-5021-01A | 70 | Lung Squamous Cell Carcinoma | M0 | N0 | T2a | male | Dead | Squamous cell carcinoma, NOS | stage ib |
| TCGA-39-5022-01A | 76 | Lung Squamous Cell Carcinoma | M0 | N0 | T2a | male | Dead | Squamous cell carcinoma, NOS | stage ib |
| TCGA-39-5024-01A | 65 | Lung Squamous Cell Carcinoma | M0 | N2 | T2a | female | Alive | Squamous cell carcinoma, NOS | stage iiia |
| TCGA-39-5027-01A | 73 | Lung Squamous Cell Carcinoma | M0 | N0 | T2a | male | Alive | Squamous cell carcinoma, NOS | stage ib |
| TCGA-39-5028-01A | 75 | Lung Squamous Cell Carcinoma | M0 | N1 | T4 | male | Dead | Squamous cell carcinoma, NOS | stage iiia |
| TCGA-39-5029-01A | 67 | Lung Squamous Cell Carcinoma | M0 | N2 | T1b | male | Dead | Squamous cell carcinoma, NOS | stage iiia |
| TCGA-39-5030-01A | 81 | Lung Squamous Cell Carcinoma | M0 | N2 | T2a | female | Dead | Squamous cell carcinoma, NOS | stage iiia |
| TCGA-39-5031-01A | 76 | Lung Squamous Cell Carcinoma | M0 | N0 | T1a | female | Dead | Squamous cell carcinoma, NOS | stage ia |
| TCGA-39-5034-01A | 73 | Lung Squamous Cell Carcinoma | M0 | N0 | T3 | female | Dead | Squamous cell carcinoma, NOS | stage iib |
| TCGA-39-5035-01A | 72 | Lung Squamous Cell Carcinoma | M0 | N0 | T1b | female | Alive | Squamous cell carcinoma, NOS | stage ia |
| TCGA-39-5036-01A | 73 | Lung Squamous Cell Carcinoma | M0 | N0 | T2 | male | Alive | Squamous cell carcinoma, NOS | stage ib |
| TCGA-39-5037-01A | 65 | Lung Squamous Cell Carcinoma | M0 | N1 | T1b | male | Alive | Squamous cell carcinoma, NOS | stage iia |
| TCGA-39-5039-01A | 76 | Lung Squamous Cell Carcinoma | M0 | N0 | T2b | male | Dead | Squamous cell carcinoma, NOS | stage iia |
| TCGA-39-5040-01A | 59 | Lung Squamous Cell Carcinoma | M0 | N2 | T2a | male | Dead | Squamous cell carcinoma, NOS | stage iiia |
| TCGA-43-2576-01A | 62 | Lung Squamous Cell Carcinoma | M0 | N2 | T2 | female | Alive | Squamous cell carcinoma, NOS | stage iiia |

| | | | | | | | | | |
|---|---|---|---|---|---|---|---|---|---|
| TCGA-43-2578-01A | 59 | Lung Squamous Cell Carcinoma | M0 | N0 | T1 | female | Dead | Squamous cell carcinoma, NOS | stage ia |
| TCGA-43-2581-01A | 47 | Lung Squamous Cell Carcinoma | M0 | N1 | T3 | female | Alive | Squamous cell carcinoma, NOS | stage iiia |
| TCGA-43-3394-01A | 52 | Lung Squamous Cell Carcinoma | M0 | N0 | T2a | male | Dead | Squamous cell carcinoma, NOS | stage ib |
| TCGA-43-3920-01A | 71 | Lung Squamous Cell Carcinoma | M0 | N0 | T2 | male | Alive | Squamous cell carcinoma, NOS | stage ib |
| TCGA-43-5668-01A | 78 | Lung Squamous Cell Carcinoma | M0 | N1 | T1b | male | Dead | Squamous cell carcinoma, NOS | stage iia |
| TCGA-43-5670-01A | 70 | Lung Squamous Cell Carcinoma | M0 | N0 | T2b | male | Alive | Squamous cell carcinoma, NOS | stage iia |
| TCGA-43-6143-01A | 70 | Lung Squamous Cell Carcinoma | M0 | N0 | T2 | male | Alive | Basaloid squamous cell carcinoma | stage ib |
| TCGA-43-6647-01A | 69 | Lung Squamous Cell Carcinoma | MX | N1 | T2b | female | Alive | Squamous cell carcinoma, NOS | stage iib |
| TCGA-43-6770-01A | 59 | Lung Squamous Cell Carcinoma | MX | N0 | T2a | female | Alive | Squamous cell carcinoma, NOS | stage ib |
| TCGA-43-6771-01A | 85 | Lung Squamous Cell Carcinoma | MX | N0 | T2 | male | Dead | Squamous cell carcinoma, NOS | stage ib |
| TCGA-43-6773-01A | 76 | Lung Squamous Cell Carcinoma | MX | N1 | T2 | male | Dead | Squamous cell carcinoma, NOS | stage iib |
| TCGA-43-7656-01A | 71 | Lung Squamous Cell Carcinoma | MX | N0 | T1b | male | Alive | Squamous cell carcinoma, NOS | stage ia |
| TCGA-43-7657-01A | 68 | Lung Squamous Cell Carcinoma | MX | N0 | T1 | female | Alive | Squamous cell carcinoma, NOS | stage ia |
| TCGA-43-7658-01A | 75 | Lung Squamous Cell Carcinoma | M0 | N0 | T1 | female | Dead | Squamous cell carcinoma, NOS | stage ia |
| TCGA-43-8115-01A | 72 | Lung Squamous Cell Carcinoma | MX | N1 | T2a | female | Alive | Squamous cell carcinoma, NOS | stage iia |
| TCGA-43-8116-01A | 73 | Lung Squamous Cell Carcinoma | M0 | N0 | T1b | male | Alive | Squamous cell carcinoma, NOS | stage ia |
| TCGA-43-8118-01A | 55 | Lung Squamous Cell Carcinoma | M0 | N0 | T1b | female | Dead | Squamous cell carcinoma, NOS | stage ia |
| TCGA-43-A474-01A | 66 | Lung Squamous Cell Carcinoma | M0 | N0 | T2b | male | Alive | Squamous cell carcinoma, NOS | stage iia |
| TCGA-43-A475-01A | 67 | Lung Squamous Cell Carcinoma | M0 | N0 | T3 | female | Alive | Squamous cell carcinoma, NOS | stage iib |
| TCGA-43-A56U-01A | 76 | Lung Squamous Cell Carcinoma | MX | N0 | T1b | female | Alive | Squamous cell carcinoma, NOS | stage ia |
| TCGA-43-A56V-01A | 61 | Lung Squamous Cell Carcinoma | M0 | N2 | T2a | male | Alive | Squamous cell carcinoma, NOS | stage iiia |
| TCGA-46-3765-01A | 59 | Lung Squamous Cell Carcinoma | M0 | N0 | T1 | female | Alive | Squamous cell carcinoma, NOS | stage ia |
| TCGA-46-3766-01A | 62 | Lung Squamous Cell Carcinoma | M0 | N0 | T1 | female | Alive | Squamous cell carcinoma, NOS | stage ia |
| TCGA-46-3767-01A | 76 | Lung Squamous Cell Carcinoma | M0 | N0 | T1a | male | Alive | Squamous cell carcinoma, NOS | stage ia |

| | | | | | | | | | |
|---|---|---|---|---|---|---|---|---|---|
| TCGA-46-3768-01A | 58 | Lung Squamous Cell Carcinoma | M0 | N1 | T3 | male | Dead | Squamous cell carcinoma, NOS | stage iiia |
| TCGA-46-3769-01A | 57 | Lung Squamous Cell Carcinoma | M0 | N0 | T4 | male | Alive | Squamous cell carcinoma, NOS | not reported |
| TCGA-46-6025-01A | 71 | Lung Squamous Cell Carcinoma | M0 | N1 | T2b | male | Alive | Squamous cell carcinoma, NOS | stage iib |
| TCGA-46-6026-01A | 81 | Lung Squamous Cell Carcinoma | M0 | N1 | T2a | male | Alive | Squamous cell carcinoma, NOS | stage iib |
| TCGA-51-4079-01A | 73 | Lung Squamous Cell Carcinoma | | N0 | T2 | female | Dead | Squamous cell carcinoma, NOS | stage ib |
| TCGA-51-4080-01A | 65 | Lung Squamous Cell Carcinoma | | N1 | T4 | male | Dead | Basaloid squamous cell carcinoma | stage iiib |
| TCGA-51-4081-01A | 55 | Lung Squamous Cell Carcinoma | M0 | N1 | T2a | male | Alive | Squamous cell carcinoma, NOS | stage iib |
| TCGA-51-6867-01A | 72 | Lung Squamous Cell Carcinoma | M0 | N0 | T1 | female | Dead | Squamous cell carcinoma, NOS | stage i |
| TCGA-52-7622-01A | 62 | Lung Squamous Cell Carcinoma | M0 | N0 | T1a | female | Alive | Squamous cell carcinoma, NOS | stage ia |
| TCGA-52-7809-01A | 74 | Lung Squamous Cell Carcinoma | M0 | N0 | T2 | male | Dead | Basaloid squamous cell carcinoma | stage ib |
| TCGA-52-7810-01A | 60 | Lung Squamous Cell Carcinoma | M0 | N0 | T3 | female | Alive | Squamous cell carcinoma, NOS | stage iib |
| TCGA-52-7811-01A | 67 | Lung Squamous Cell Carcinoma | M0 | N0 | T2 | male | Dead | Squamous cell carcinoma, NOS | stage ib |
| TCGA-52-7812-01A | 68 | Lung Squamous Cell Carcinoma | M0 | N2 | T2 | male | Dead | Squamous cell carcinoma, NOS | not reported |
| TCGA-56-1622-01A | 58 | Lung Squamous Cell Carcinoma | M0 | N0 | T2 | male | Dead | Squamous cell carcinoma, NOS | stage ib |
| TCGA-56-5897-01A | 74 | Lung Squamous Cell Carcinoma | MX | N0 | T1b | male | Alive | Squamous cell carcinoma, NOS | stage ia |
| TCGA-56-5898-01A | 69 | Lung Squamous Cell Carcinoma | M0 | N0 | T1b | male | Alive | Squamous cell carcinoma, NOS | stage ia |
| TCGA-56-6545-01A | 77 | Lung Squamous Cell Carcinoma | M0 | N0 | T2a | female | Alive | Squamous cell carcinoma, NOS | stage ib |
| TCGA-56-7221-01A | 79 | Lung Squamous Cell Carcinoma | M0 | N0 | T2 | male | Alive | Squamous cell carcinoma, NOS | stage ib |
| TCGA-56-7222-01A | 60 | Lung Squamous Cell Carcinoma | M0 | N0 | T2a | male | Dead | Squamous cell carcinoma, NOS | stage ib |
| TCGA-56-7223-01A | 66 | Lung Squamous Cell Carcinoma | MX | N1 | T3 | male | Dead | Squamous cell carcinoma, NOS | stage iiia |
| TCGA-56-7579-01A | 61 | Lung Squamous Cell Carcinoma | M0 | N1 | T3 | male | Dead | Squamous cell carcinoma, NOS | stage iiia |
| TCGA-56-7580-01A | 84 | Lung Squamous Cell Carcinoma | M0 | N0 | T2a | male | Alive | Squamous cell carcinoma, NOS | stage ib |
| TCGA-56-7582-01A | 83 | Lung Squamous Cell Carcinoma | M0 | N0 | T2a | male | Alive | Squamous cell carcinoma, NOS | stage ib |

| | | | | | | | | | |
|---|---|---|---|---|---|---|---|---|---|
| TCGA-56-7730-01A | 73 | Lung Squamous Cell Carcinoma | MO | NO | T2b | male | Dead | Squamous cell carcinoma, NOS | stage iia |
| TCGA-56-7731-01A | 66 | Lung Squamous Cell Carcinoma | MX | NO | T2a | female | Dead | Squamous cell carcinoma, NOS | stage ib |
| TCGA-56-7822-01A | 75 | Lung Squamous Cell Carcinoma | MO | N1 | T2b | male | Dead | Squamous cell carcinoma, NOS | stage iib |
| TCGA-56-7823-01B | 58 | Lung Squamous Cell Carcinoma | MO | N1 | T1b | female | Alive | Squamous cell carcinoma, NOS | stage iia |
| TCGA-56-8082-01A | 80 | Lung Squamous Cell Carcinoma | MX | NO | T2b | female | Alive | Squamous cell carcinoma, NOS | stage iia |
| TCGA-56-8083-01A | 56 | Lung Squamous Cell Carcinoma | MX | NO | T2a | male | Alive | Squamous cell carcinoma, NOS | stage ib |
| TCGA-56-8201-01A | 74 | Lung Squamous Cell Carcinoma | MX | NO | T3 | male | Dead | Squamous cell carcinoma, NOS | stage iib |
| TCGA-56-8304-01A | 73 | Lung Squamous Cell Carcinoma | MX | NO | T1b | female | Alive | Squamous cell carcinoma, NOS | stage ia |
| TCGA-56-8305-01A | 72 | Lung Squamous Cell Carcinoma | MO | NO | T2a | male | Alive | Squamous cell carcinoma, NOS | stage ib |
| TCGA-56-8307-01A | 55 | Lung Squamous Cell Carcinoma | MO | NO | T3 | female | Alive | Squamous cell carcinoma, NOS | stage iib |
| TCGA-56-8308-01A | 79 | Lung Squamous Cell Carcinoma | MX | NO | T3 | male | Alive | Squamous cell carcinoma, NOS | stage iib |
| TCGA-56-8309-01A | 66 | Lung Squamous Cell Carcinoma | MX | NO | T1b | male | Alive | Squamous cell carcinoma, NOS | stage ia |
| TCGA-56-8503-01A | 76 | Lung Squamous Cell Carcinoma | MO | NO | T3 | female | Alive | Squamous cell carcinoma, NOS | stage iib |
| TCGA-56-8504-01A | 74 | Lung Squamous Cell Carcinoma | MX | NO | T2a | male | Alive | Squamous cell carcinoma, NOS | stage ib |
| TCGA-56-8622-01A | 68 | Lung Squamous Cell Carcinoma | MO | NO | T2a | male | Alive | Squamous cell carcinoma, NOS | stage ib |
| TCGA-56-8623-01A | 71 | Lung Squamous Cell Carcinoma | MX | NO | T2a | male | Dead | Squamous cell carcinoma, NOS | stage ib |
| TCGA-56-8624-01A | 84 | Lung Squamous Cell Carcinoma | MX | NO | T3 | male | Alive | Squamous cell carcinoma, NOS | stage iib |
| TCGA-56-8625-01A | 66 | Lung Squamous Cell Carcinoma | MX | N1 | T3 | female | Dead | Squamous cell carcinoma, NOS | stage iiia |
| TCGA-56-8626-01A | 59 | Lung Squamous Cell Carcinoma | MX | NO | T1a | male | Dead | Squamous cell carcinoma, NOS | stage ia |
| TCGA-56-8628-01A | 78 | Lung Squamous Cell Carcinoma | MX | NO | T1b | male | Alive | Squamous cell carcinoma, NOS | stage ia |
| TCGA-56-8629-01A | 63 | Lung Squamous Cell Carcinoma | MX | NO | T2b | male | Alive | Squamous cell carcinoma, NOS | stage iia |
| TCGA-56-A49D-01A | 67 | Lung Squamous Cell Carcinoma | MX | N2 | T2a | male | Alive | Squamous cell carcinoma, NOS | stage iiia |
| TCGA-56-A4BW-01A | 55 | Lung Squamous Cell Carcinoma | MO | N1 | T2a | male | Alive | Squamous cell carcinoma, NOS | stage iia |
| TCGA-56-A4BX-01A | 70 | Lung Squamous Cell Carcinoma | MX | NO | T2b | male | Alive | Squamous cell carcinoma, NOS | stage iia |

| | | | | | | | | | |
|---|---|---|---|---|---|---|---|---|---|
| TCGA-56-A4BY-01A | 66 | Lung Squamous Cell Carcinoma | MX | N0 | T2a | male | Dead | Squamous cell carcinoma, keratinizing, NOS | stage ib |
| TCGA-56-A4ZJ-01A | 75 | Lung Squamous Cell Carcinoma | M0 | N0 | T1a | female | Alive | Squamous cell carcinoma, large cell, nonkeratinizing, NOS | stage ia |
| TCGA-56-A4ZK-01A | 76 | Lung Squamous Cell Carcinoma | M0 | N0 | T2 | female | Alive | Squamous cell carcinoma, NOS | stage ib |
| TCGA-56-A5DR-01A | 81 | Lung Squamous Cell Carcinoma | MX | N0 | T1a | male | Alive | Squamous cell carcinoma, NOS | stage ia |
| TCGA-56-A5DS-01A | 72 | Lung Squamous Cell Carcinoma | MX | N0 | T2a | female | Alive | Squamous cell carcinoma, NOS | stage ib |
| TCGA-56-A62T-01A | 78 | Lung Squamous Cell Carcinoma | MX | N0 | T2b | male | Alive | Squamous cell carcinoma, NOS | stage iia |
| TCGA-58-8386-01A | 75 | Lung Squamous Cell Carcinoma | M1 | NX | T3 | male | Dead | Squamous cell carcinoma, NOS | stage iv |
| TCGA-58-8387-01A | 60 | Lung Squamous Cell Carcinoma | M0 | N0 | T2b | male | Dead | Squamous cell carcinoma, NOS | stage iia |
| TCGA-58-8388-01A | 60 | Lung Squamous Cell Carcinoma | M0 | N0 | T2a | male | Dead | Basaloid squamous cell carcinoma | stage ib |
| TCGA-58-8390-01A | 70 | Lung Squamous Cell Carcinoma | M0 | N0 | T2b | male | Alive | Squamous cell carcinoma, NOS | stage iia |
| TCGA-58-8391-01A | 57 | Lung Squamous Cell Carcinoma | M0 | N2 | T2 | female | Alive | Squamous cell carcinoma, NOS | stage iiia |
| TCGA-58-8392-01A | 70 | Lung Squamous Cell Carcinoma | M0 | N0 | T2a | male | Dead | Squamous cell carcinoma, NOS | stage ib |
| TCGA-58-8393-01A | 68 | Lung Squamous Cell Carcinoma | M0 | N0 | T2a | female | Alive | Squamous cell carcinoma, NOS | stage ib |
| TCGA-58-A46J-01A | 64 | Lung Squamous Cell Carcinoma | M0 | N1 | T2 | male | Alive | Squamous cell carcinoma, NOS | stage iib |
| TCGA-58-A46K-01A | 59 | Lung Squamous Cell Carcinoma | M0 | N2 | T2 | male | Dead | Basaloid squamous cell carcinoma | stage iiia |
| TCGA-58-A46L-01A | 73 | Lung Squamous Cell Carcinoma | M0 | N2 | T2 | male | Alive | Basaloid squamous cell carcinoma | stage iiia |
| TCGA-58-A46M-01A | 61 | Lung Squamous Cell Carcinoma | M0 | N1 | T2b | male | Alive | Squamous cell carcinoma, NOS | stage iib |
| TCGA-58-A46N-01A | 52 | Lung Squamous Cell Carcinoma | M0 | N0 | T2a | male | Alive | Basaloid squamous cell carcinoma | stage ib |

| | | | | | | | | | |
|---|---|---|---|---|---|---|---|---|---|
| TCGA-60-2695-01A | 74 | Lung Squamous Cell Carcinoma | M0 | N0 | T2 | female | Alive | Basaloid squamous cell carcinoma | stage ib |
| TCGA-60-2696-01A | 76 | Lung Squamous Cell Carcinoma | M0 | N0 | T2 | female | Dead | Squamous cell carcinoma, NOS | stage iia |
| TCGA-60-2697-01A | 41 | Lung Squamous Cell Carcinoma | M0 | N2 | T2 | male | Dead | Squamous cell carcinoma, NOS | stage iia |
| TCGA-60-2698-01A | 62 | Lung Squamous Cell Carcinoma | M0 | N1 | T2 | male | Dead | Squamous cell carcinoma, NOS | stage iib |
| TCGA-60-2703-01A | 73 | Lung Squamous Cell Carcinoma | M0 | N1 | T2 | male | Dead | Squamous cell carcinoma, NOS | stage iib |
| TCGA-60-2704-01A | 73 | Lung Squamous Cell Carcinoma | M0 | N1 | T2 | male | Dead | Squamous cell carcinoma, NOS | stage iib |
| TCGA-60-2706-01A | 58 | Lung Squamous Cell Carcinoma | M0 | N0 | T1 | male | Alive | Squamous cell carcinoma, NOS | stage ia |
| TCGA-60-2707-01A | 70 | Lung Squamous Cell Carcinoma | M0 | N0 | T2 | male | Dead | Squamous cell carcinoma, NOS | stage ib |
| TCGA-60-2708-01A | 64 | Lung Squamous Cell Carcinoma | M0 | N1 | T2 | female | Alive | Squamous cell carcinoma, NOS | stage iib |
| TCGA-60-2709-01A | 69 | Lung Squamous Cell Carcinoma | MX | N0 | T2 | male | Alive | Squamous cell carcinoma, NOS | stage ib |
| TCGA-60-2710-01A | 67 | Lung Squamous Cell Carcinoma | M0 | N1 | T1 | female | Alive | Squamous cell carcinoma, NOS | stage iia |
| TCGA-60-2711-01A | 64 | Lung Squamous Cell Carcinoma | M0 | N0 | T2 | female | Alive | Squamous cell carcinoma, NOS | stage ib |
| TCGA-60-2712-01A | 79 | Lung Squamous Cell Carcinoma | M0 | N1 | T2 | female | Dead | Squamous cell carcinoma, NOS | stage iib |
| TCGA-60-2713-01A | 64 | Lung Squamous Cell Carcinoma | M0 | N0 | T2 | male | Alive | Squamous cell carcinoma, NOS | stage ib |
| TCGA-60-2714-01A | 66 | Lung Squamous Cell Carcinoma | M0 | N1 | T2 | female | Alive | Squamous cell carcinoma, NOS | stage iib |
| TCGA-60-2715-01A | 51 | Lung Squamous Cell Carcinoma | M0 | N0 | T1 | male | Dead | Squamous cell carcinoma, NOS | stage ia |
| TCGA-60-2716-01A | 39 | Lung Squamous Cell Carcinoma | M0 | N1 | T2 | male | Alive | Squamous cell carcinoma, NOS | stage iib |
| TCGA-60-2719-01A | 83 | Lung Squamous Cell Carcinoma | M0 | N0 | T1 | female | Alive | Squamous cell carcinoma, NOS | stage ib |
| TCGA-60-2720-01A | 60 | Lung Squamous Cell Carcinoma | M0 | N0 | T2 | female | Alive | Squamous cell carcinoma, NOS | stage ib |
| TCGA-60-2721-01A | 73 | Lung Squamous Cell Carcinoma | M0 | N0 | T2 | male | Alive | Squamous cell carcinoma, NOS | stage ib |
| TCGA-60-2722-01A | 66 | Lung Squamous Cell Carcinoma | M0 | N1 | T2 | male | Alive | Squamous cell carcinoma, NOS | stage iib |
| TCGA-60-2723-01A | 74 | Lung Squamous Cell Carcinoma | M0 | N0 | T2 | female | Alive | Squamous cell carcinoma, NOS | stage ib |
| TCGA-60-2724-01A | 47 | Lung Squamous Cell Carcinoma | M0 | N1 | T3 | male | Alive | Squamous cell carcinoma, NOS | stage iiia |
| TCGA-60-2725-01A | 74 | Lung Squamous Cell Carcinoma | M0 | N0 | T2 | male | Alive | Squamous cell carcinoma, NOS | stage ib |

| | | | | | | | | | |
|---|---|---|---|---|---|---|---|---|---|
| TCGA-60-2726-01A | 56 | Lung Squamous Cell Carcinoma | M0 | N1 | T2 | male | Dead | Squamous cell carcinoma, NOS | stage iia |
| TCGA-63-6202-01A | | Lung Squamous Cell Carcinoma | M0 | N0 | T2 | male | Alive | Squamous cell carcinoma, NOS | stage iia |
| TCGA-63-7020-01A | | Lung Squamous Cell Carcinoma | M0 | N0 | T1 | male | Alive | Squamous cell carcinoma, NOS | stage ia |
| TCGA-63-7021-01A | | Lung Squamous Cell Carcinoma | M0 | N0 | T1 | male | Alive | Squamous cell carcinoma, NOS | stage ia |
| TCGA-63-7022-01A | | Lung Squamous Cell Carcinoma | M0 | N0 | T1 | female | Alive | Squamous cell carcinoma, NOS | stage ia |
| TCGA-63-7023-01A | | Lung Squamous Cell Carcinoma | M0 | N1 | T1 | male | Alive | Squamous cell carcinoma, NOS | stage iia |
| TCGA-63-A5MB-01A | 62 | Lung Squamous Cell Carcinoma | M0 | N0 | T2 | male | Alive | Squamous cell carcinoma, NOS | stage ib |
| TCGA-63-A5MG-01A | 68 | Lung Squamous Cell Carcinoma | M0 | N0 | T2 | male | Alive | Squamous cell carcinoma, NOS | stage ib |
| TCGA-63-A5MH-01A | 68 | Lung Squamous Cell Carcinoma | M0 | N0 | T1 | male | Alive | Squamous cell carcinoma, NOS | stage ia |
| TCGA-63-A5MI-01A | 65 | Lung Squamous Cell Carcinoma | M0 | N2 | T2 | male | Alive | Squamous cell carcinoma, NOS | stage iiia |
| TCGA-63-A5MJ-01A | 54 | Lung Squamous Cell Carcinoma | M0 | N1 | T2 | male | Alive | Squamous cell carcinoma, NOS | stage iib |
| TCGA-63-A5ML-01A | 68 | Lung Squamous Cell Carcinoma | M0 | N0 | T2 | male | Alive | Squamous cell carcinoma, NOS | stage ib |
| TCGA-63-A5MM-01A | 69 | Lung Squamous Cell Carcinoma | M0 | N1 | T2 | female | Dead | Squamous cell carcinoma, NOS | stage iib |
| TCGA-63-A5MN-01A | 78 | Lung Squamous Cell Carcinoma | M0 | N0 | T3 | female | Dead | Squamous cell carcinoma, NOS | stage iib |
| TCGA-63-A5MP-01A | 56 | Lung Squamous Cell Carcinoma | M0 | N1 | T2 | male | Alive | Squamous cell carcinoma, NOS | stage iib |
| TCGA-63-A5MR-01A | 70 | Lung Squamous Cell Carcinoma | M0 | N0 | T2 | female | Alive | Squamous cell carcinoma, NOS | stage ib |
| TCGA-63-A5MS-01A | 78 | Lung Squamous Cell Carcinoma | M0 | N0 | T2 | male | Alive | Squamous cell carcinoma, NOS | stage ib |
| TCGA-63-A5MT-01A | 74 | Lung Squamous Cell Carcinoma | M0 | N0 | T3 | male | Alive | Squamous cell carcinoma, NOS | stage iib |
| TCGA-63-A5MV-01A | 69 | Lung Squamous Cell Carcinoma | M0 | N0 | T2b | male | Alive | Squamous cell carcinoma, NOS | stage iia |
| TCGA-63-A5MW-01A | 76 | Lung Squamous Cell Carcinoma | M0 | N0 | T2 | male | Alive | Squamous cell carcinoma, NOS | stage ib |
| TCGA-63-A5MY-01A | 63 | Lung Squamous Cell Carcinoma | M0 | N0 | T1b | male | Alive | Squamous cell carcinoma, NOS | stage ia |
| TCGA-66-2727-01A | 55 | Lung Squamous Cell Carcinoma | M0 | N0 | T2 | female | Dead | Squamous cell carcinoma, NOS | stage ib |
| TCGA-66-2734-01A | 62 | Lung Squamous Cell Carcinoma | M0 | N0 | T2 | female | Alive | Squamous cell carcinoma, NOS | stage ib |
| TCGA-66-2737-01A | 72 | Lung Squamous Cell Carcinoma | M0 | N1 | T2 | male | Alive | Squamous cell carcinoma, NOS | stage iib |
| TCGA-66-2742-01A | 70 | Lung Squamous Cell Carcinoma | M1 | N1 | T2 | male | Alive | Squamous cell carcinoma, NOS | stage iv |

| | | | | | | | | | | |
|---|---|---|---|---|---|---|---|---|---|---|
| TCGA-66-2744-01A | 71 | Lung Squamous Cell Carcinoma | M0 | N1 | T2 | male | Alive | Squamous cell carcinoma, NOS | stage iib |
| TCGA-66-2753-01A | 69 | Lung Squamous Cell Carcinoma | M0 | N0 | T2 | male | Alive | Squamous cell carcinoma, NOS | stage ib |
| TCGA-66-2754-01A | 67 | Lung Squamous Cell Carcinoma | M0 | N2 | T2 | male | Alive | Squamous cell carcinoma, NOS | stage iiia |
| TCGA-66-2755-01A | 63 | Lung Squamous Cell Carcinoma | M0 | N0 | T2 | male | Alive | Squamous cell carcinoma, NOS | stage ib |
| TCGA-66-2756-01A | 68 | Lung Squamous Cell Carcinoma | M0 | N0 | T4 | male | Alive | Squamous cell carcinoma, NOS | stage iiib |
| TCGA-66-2757-01A | 65 | Lung Squamous Cell Carcinoma | M0 | N0 | T1 | female | Dead | Squamous cell carcinoma, NOS | stage ia |
| TCGA-66-2758-01A | 71 | Lung Squamous Cell Carcinoma | M0 | N0 | T2 | male | Alive | Squamous cell carcinoma, NOS | stage ib |
| TCGA-66-2759-01A | 66 | Lung Squamous Cell Carcinoma | M0 | N2 | T2 | male | Alive | Squamous cell carcinoma, NOS | stage iiia |
| TCGA-66-2763-01A | 63 | Lung Squamous Cell Carcinoma | M0 | N0 | T2 | female | Alive | Squamous cell carcinoma, NOS | stage ib |
| TCGA-66-2765-01A | 64 | Lung Squamous Cell Carcinoma | M0 | N0 | T2 | male | Alive | Squamous cell carcinoma, NOS | stage ib |
| TCGA-66-2766-01A | 54 | Lung Squamous Cell Carcinoma | M0 | N2 | T2 | male | Alive | Squamous cell carcinoma, NOS | stage iiia |
| TCGA-66-2767-01A | 62 | Lung Squamous Cell Carcinoma | M0 | N3 | T2 | male | Alive | Squamous cell carcinoma, NOS | stage iiib |
| TCGA-66-2768-01A | 57 | Lung Squamous Cell Carcinoma | M0 | N1 | T2 | male | Alive | Squamous cell carcinoma, NOS | stage iib |
| TCGA-66-2769-01A | 75 | Lung Squamous Cell Carcinoma | M0 | N0 | T4 | male | Dead | Squamous cell carcinoma, NOS | stage iiib |
| TCGA-66-2770-01A | 79 | Lung Squamous Cell Carcinoma | M0 | N0 | T2 | male | Alive | Squamous cell carcinoma, NOS | stage ib |
| TCGA-66-2771-01A | 60 | Lung Squamous Cell Carcinoma | M0 | N1 | T2 | male | Alive | Squamous cell carcinoma, NOS | stage iib |
| TCGA-66-2773-01A | 69 | Lung Squamous Cell Carcinoma | M0 | N0 | T2 | male | Dead | Squamous cell carcinoma, NOS | stage ib |
| TCGA-66-2777-01A | 71 | Lung Squamous Cell Carcinoma | M0 | N0 | T2 | male | Alive | Squamous cell carcinoma, NOS | stage ib |
| TCGA-66-2778-01A | 68 | Lung Squamous Cell Carcinoma | M0 | N3 | T2 | female | Alive | Squamous cell carcinoma, NOS | stage iiib |
| TCGA-66-2780-01A | 65 | Lung Squamous Cell Carcinoma | M0 | N0 | T2 | male | Dead | Squamous cell carcinoma, NOS | stage ib |
| TCGA-66-2781-01A | 67 | Lung Squamous Cell Carcinoma | M0 | N0 | T2 | male | Alive | Squamous cell carcinoma, NOS | stage ib |
| TCGA-66-2782-01A | 71 | Lung Squamous Cell Carcinoma | M0 | N0 | T3 | male | Dead | Squamous cell carcinoma, NOS | stage iib |
| TCGA-66-2783-01A | 67 | Lung Squamous Cell Carcinoma | M0 | N3 | T2 | male | Alive | Squamous cell carcinoma, NOS | stage iiib |
| TCGA-66-2785-01A | 65 | Lung Squamous Cell Carcinoma | M0 | N0 | T2 | male | Alive | Squamous cell carcinoma, NOS | stage ib |
| TCGA-66-2786-01A | 68 | Lung Squamous Cell Carcinoma | M0 | N0 | T1 | female | Alive | Squamous cell carcinoma, NOS | stage ia |

| | | | | | | | | | |
|---|---|---|---|---|---|---|---|---|---|
| TCGA-66-2787-01A | 57 | Lung Squamous Cell Carcinoma | M0 | N0 | T1 | male | Alive | Squamous cell carcinoma, NOS | stage ia |
| TCGA-66-2788-01A | 56 | Lung Squamous Cell Carcinoma | M0 | N0 | T2 | male | Alive | Squamous cell carcinoma, NOS | stage ib |
| TCGA-66-2789-01A | 73 | Lung Squamous Cell Carcinoma | M0 | N3 | T1 | male | Dead | Squamous cell carcinoma, NOS | stage iiib |
| TCGA-66-2790-01A | 72 | Lung Squamous Cell Carcinoma | M0 | N1 | T2 | male | Alive | Squamous cell carcinoma, NOS | stage iib |
| TCGA-66-2791-01A | 66 | Lung Squamous Cell Carcinoma | M0 | N3 | T2 | male | Dead | Squamous cell carcinoma, NOS | stage iiib |
| TCGA-66-2792-01A | 58 | Lung Squamous Cell Carcinoma | M0 | N1 | T2 | male | Alive | Squamous cell carcinoma, NOS | stage iib |
| TCGA-66-2793-01A | 68 | Lung Squamous Cell Carcinoma | M0 | N1 | T4 | male | Dead | Squamous cell carcinoma, NOS | stage iiib |
| TCGA-66-2794-01A | 64 | Lung Squamous Cell Carcinoma | M0 | N2 | T4 | male | Alive | Squamous cell carcinoma, NOS | stage iiib |
| TCGA-66-2795-01A | 68 | Lung Squamous Cell Carcinoma | M0 | N1 | T4 | male | Alive | Squamous cell carcinoma, NOS | stage iiib |
| TCGA-66-2800-01A | 70 | Lung Squamous Cell Carcinoma | M0 | N0 | T4 | male | Alive | Squamous cell carcinoma, NOS | stage iiib |
| TCGA-68-7755-01A | 60 | Lung Squamous Cell Carcinoma | M0 | N1 | T1b | female | Alive | Squamous cell carcinoma, NOS | stage iia |
| TCGA-68-7756-01A | 84 | Lung Squamous Cell Carcinoma | MX | N1 | T4 | male | Alive | Squamous cell carcinoma, NOS | stage iiia |
| TCGA-68-7757-01B | 74 | Lung Squamous Cell Carcinoma | MX | N0 | T1b | male | Alive | Squamous cell carcinoma, NOS | stage ia |
| TCGA-68-8250-01A | 66 | Lung Squamous Cell Carcinoma | MX | N0 | T1a | male | Alive | Squamous cell carcinoma, NOS | stage ia |
| TCGA-68-8251-01A | 78 | Lung Squamous Cell Carcinoma | M0 | N0 | T2a | male | Alive | Squamous cell carcinoma, NOS | stage ib |
| TCGA-68-A59I-01A | 73 | Lung Squamous Cell Carcinoma | M0 | N1 | T3 | female | Alive | Squamous cell carcinoma, NOS | stage iiia |
| TCGA-68-A59J-01A | 74 | Lung Squamous Cell Carcinoma | MX | N0 | T2a | female | Alive | Squamous cell carcinoma, NOS | stage ib |
| TCGA-70-6722-01A | 47 | Lung Squamous Cell Carcinoma | M0 | N1 | T3 | male | Alive | Squamous cell carcinoma, NOS | stage iiia |
| TCGA-70-6723-01A | 65 | Lung Squamous Cell Carcinoma | M0 | N0 | T3 | male | Alive | Papillary squamous cell carcinoma | stage iia |
| TCGA-77-6842-01A | 79 | Lung Squamous Cell Carcinoma | M0 | N1 | T2 | male | Dead | Squamous cell carcinoma, NOS | stage iib |
| TCGA-77-6843-01A | 74 | Lung Squamous Cell Carcinoma | M0 | N1 | T1 | male | Dead | Squamous cell carcinoma, NOS | stage iia |
| TCGA-77-6844-01A | 74 | Lung Squamous Cell Carcinoma | M0 | N1 | T3 | male | Dead | Squamous cell carcinoma, NOS | stage iiia |
| TCGA-77-6845-01A | 69 | Lung Squamous Cell Carcinoma | M0 | N0 | T3 | male | Dead | Squamous cell carcinoma, NOS | stage iib |
| TCGA-77-7138-01A | 67 | Lung Squamous Cell Carcinoma | M0 | N0 | T2 | male | Dead | Squamous cell carcinoma, NOS | stage ib |

| | | | | | | | | | |
|---|---|---|---|---|---|---|---|---|---|
| TCGA-77-7139-01A | 56 | Lung Squamous Cell Carcinoma | M0 | N1 | T2 | male | Alive | Squamous cell carcinoma, NOS | stage iib |
| TCGA-77-7140-01A | 69 | Lung Squamous Cell Carcinoma | M0 | N1 | T2 | female | Dead | Squamous cell carcinoma, NOS | stage iib |
| TCGA-77-7141-01A | 64 | Lung Squamous Cell Carcinoma | M0 | N0 | T2 | male | Alive | Squamous cell carcinoma, NOS | stage ib |
| TCGA-77-7142-01A | 59 | Lung Squamous Cell Carcinoma | M0 | N0 | T2 | female | Alive | Squamous cell carcinoma, NOS | stage ib |
| TCGA-77-7335-01A | 62 | Lung Squamous Cell Carcinoma | M0 | N2 | T4 | female | Dead | Squamous cell carcinoma, keratinizing, NOS | stage iiib |
| TCGA-77-7337-01A | 65 | Lung Squamous Cell Carcinoma | M0 | N1 | T2 | male | Dead | Squamous cell carcinoma, NOS | stage iib |
| TCGA-77-7338-01A | 64 | Lung Squamous Cell Carcinoma | M0 | N0 | T2 | male | Dead | Squamous cell carcinoma, NOS | stage ib |
| TCGA-77-7463-01A | 75 | Lung Squamous Cell Carcinoma | M0 | N0 | T2 | male | Dead | Squamous cell carcinoma, NOS | stage ib |
| TCGA-77-7465-01A | 58 | Lung Squamous Cell Carcinoma | M0 | N1 | T2a | male | Alive | Squamous cell carcinoma, NOS | stage iia |
| TCGA-77-8007-01A | 68 | Lung Squamous Cell Carcinoma | M0 | N1 | T2 | male | Dead | Squamous cell carcinoma, NOS | stage iib |
| TCGA-77-8008-01A | 68 | Lung Squamous Cell Carcinoma | M0 | N0 | T2 | male | Dead | Squamous cell carcinoma, keratinizing, NOS | stage ib |
| TCGA-77-8009-01A | 68 | Lung Squamous Cell Carcinoma | M0 | N1 | T2 | male | Dead | Squamous cell carcinoma, NOS | stage iib |
| TCGA-77-8128-01A | 60 | Lung Squamous Cell Carcinoma | M0 | N2 | T2 | male | Dead | Squamous cell carcinoma, NOS | stage iiia |
| TCGA-77-8130-01A | 69 | Lung Squamous Cell Carcinoma | M0 | N1 | T2 | male | Alive | Squamous cell carcinoma, NOS | stage iib |
| TCGA-77-8131-01A | 72 | Lung Squamous Cell Carcinoma | M0 | NX | T2 | male | Dead | Squamous cell carcinoma, NOS | stage ib |
| TCGA-77-8133-01A | 74 | Lung Squamous Cell Carcinoma | M0 | N1 | T1 | male | Dead | Squamous cell carcinoma, NOS | stage iia |
| TCGA-77-8136-01A | 74 | Lung Squamous Cell Carcinoma | M0 | N1 | T2 | female | Dead | Squamous cell carcinoma, NOS | stage iib |
| TCGA-77-8138-01A | 74 | Lung Squamous Cell Carcinoma | M0 | N0 | T2 | male | Dead | Squamous cell carcinoma, NOS | stage ib |
| TCGA-77-8139-01A | 72 | Lung Squamous Cell Carcinoma | M0 | N0 | T3 | male | Alive | Squamous cell carcinoma, NOS | stage iib |
| TCGA-77-8140-01A | 66 | Lung Squamous Cell Carcinoma | M0 | N1 | T2 | female | Dead | Squamous cell carcinoma, NOS | stage iib |
| TCGA-77-8143-01A | 76 | Lung Squamous Cell Carcinoma | M0 | N2 | T2 | male | Dead | Squamous cell carcinoma, NOS | stage iiia |
| TCGA-77-8144-01A | 70 | Lung Squamous Cell Carcinoma | M0 | N0 | T2 | male | Alive | Squamous cell carcinoma, NOS | stage ib |
| TCGA-77-8145-01A | 73 | Lung Squamous Cell Carcinoma | M0 | N1 | T4 | male | Dead | Squamous cell carcinoma, NOS | stage iiib |

| | | | | | | | | | |
|---|---|---|---|---|---|---|---|---|---|
| TCGA-77-8146-01A | 72 | Lung Squamous Cell Carcinoma | M0 | N0 | T1 | male | Alive | Squamous cell carcinoma, NOS | stage ia |
| TCGA-77-8148-01A | 68 | Lung Squamous Cell Carcinoma | M0 | N1 | T3 | male | Alive | Squamous cell carcinoma, NOS | stage iiia |
| TCGA-77-8150-01A | 64 | Lung Squamous Cell Carcinoma | M0 | N1 | T3 | male | Dead | Squamous cell carcinoma, NOS | stage iiia |
| TCGA-77-8153-01A | 77 | Lung Squamous Cell Carcinoma | M0 | N0 | T2 | female | Alive | Squamous cell carcinoma, NOS | stage ib |
| TCGA-77-8154-01A | 67 | Lung Squamous Cell Carcinoma | M0 | N0 | T1 | male | Alive | Squamous cell carcinoma, NOS | stage ia |
| TCGA-77-8156-01A | 60 | Lung Squamous Cell Carcinoma | M0 | N0 | T2a | male | Alive | Squamous cell carcinoma, NOS | stage ib |
| TCGA-77-A5FZ-01A | 64 | Lung Squamous Cell Carcinoma | M0 | N0 | T4 | male | Dead | Papillary squamous cell carcinoma | stage iiib |
| TCGA-77-A5G1-01A | 75 | Lung Squamous Cell Carcinoma | M0 | N1 | T3 | male | Alive | Squamous cell carcinoma, NOS | stage iiia |
| TCGA-77-A5G3-01A | 63 | Lung Squamous Cell Carcinoma | M0 | N1 | T2 | male | Alive | Squamous cell carcinoma, NOS | stage iib |
| TCGA-77-A5G6-01A | 66 | Lung Squamous Cell Carcinoma | M0 | N2 | T2 | male | Dead | Squamous cell carcinoma, NOS | stage iiia |
| TCGA-77-A5G7-01B | 63 | Lung Squamous Cell Carcinoma | M0 | N0 | T1 | male | Dead | Squamous cell carcinoma, NOS | stage ia |
| TCGA-77-A5G8-01B | 70 | Lung Squamous Cell Carcinoma | M0 | N0 | T3 | male | Alive | Squamous cell carcinoma, NOS | stage iib |
| TCGA-77-A5GA-01A | 76 | Lung Squamous Cell Carcinoma | M0 | N0 | T2a | male | Alive | Squamous cell carcinoma, NOS | stage ib |
| TCGA-77-A5GF-01A | 70 | Lung Squamous Cell Carcinoma | M0 | N1 | T2a | male | Dead | Squamous cell carcinoma, NOS | stage iia |
| TCGA-77-A5GH-01A | 81 | Lung Squamous Cell Carcinoma | M0 | N0 | T2a | male | Alive | Squamous cell carcinoma, NOS | stage ib |
| TCGA-85-6175-01A | 63 | Lung Squamous Cell Carcinoma | M0 | N0 | T3 | female | Dead | Squamous cell carcinoma, NOS | stage iib |
| TCGA-85-6560-01A | 59 | Lung Squamous Cell Carcinoma | M0 | N1 | T1b | male | Alive | Squamous cell carcinoma, NOS | stage iia |
| TCGA-85-6561-01A | 66 | Lung Squamous Cell Carcinoma | M0 | NX | T2a | male | Alive | Squamous cell carcinoma, NOS | stage ib |
| TCGA-85-6798-01A | 57 | Lung Squamous Cell Carcinoma | M0 | N1 | T3 | male | Dead | Squamous cell carcinoma, NOS | stage iiia |
| TCGA-85-7696-01A | 64 | Lung Squamous Cell Carcinoma | M0 | N0 | T1 | male | Alive | Squamous cell carcinoma, NOS | stage ia |
| TCGA-85-7697-01A | 49 | Lung Squamous Cell Carcinoma | M0 | N0 | T3 | male | Alive | Squamous cell carcinoma, NOS | stage iib |
| TCGA-85-7698-01A | 48 | Lung Squamous Cell Carcinoma | M0 | N0 | T1b | male | Alive | Squamous cell carcinoma, NOS | stage ia |
| TCGA-85-7699-01A | 73 | Lung Squamous Cell Carcinoma | M0 | N0 | T4 | male | Dead | Squamous cell carcinoma, NOS | stage iiia |
| TCGA-85-7710-01A | 59 | Lung Squamous Cell Carcinoma | M0 | N0 | T1b | female | Alive | Squamous cell carcinoma, NOS | stage ia |

| | | | | | | | | | |
|---|---|---|---|---|---|---|---|---|---|
| TCGA-85-7843-01A | 50 | Lung Squamous Cell Carcinoma | M0 | N1 | T2a | male | Alive | Squamous cell carcinoma, NOS | stage iia |
| TCGA-85-7844-01A | 71 | Lung Squamous Cell Carcinoma | M0 | N0 | T2a | male | Alive | Squamous cell carcinoma, NOS | stage ib |
| TCGA-85-7950-01A | 46 | Lung Squamous Cell Carcinoma | M0 | N0 | T2a | male | Alive | Squamous cell carcinoma, NOS | stage ib |
| TCGA-85-8048-01A | 62 | Lung Squamous Cell Carcinoma | M0 | N0 | T1 | male | Alive | Squamous cell carcinoma, NOS | stage ia |
| TCGA-85-8049-01A | 57 | Lung Squamous Cell Carcinoma | M0 | N0 | T2a | male | Alive | Squamous cell carcinoma, NOS | stage ib |
| TCGA-85-8052-01A | 53 | Lung Squamous Cell Carcinoma | M0 | N0 | T3 | male | Alive | Squamous cell carcinoma, NOS | stage iib |
| TCGA-85-8070-01A | 71 | Lung Squamous Cell Carcinoma | M0 | N0 | T2 | male | Alive | Squamous cell carcinoma, NOS | stage ib |
| TCGA-85-8071-01A | 52 | Lung Squamous Cell Carcinoma | M0 | N1 | T1a | male | Alive | Squamous cell carcinoma, NOS | stage iia |
| TCGA-85-8072-01A | 60 | Lung Squamous Cell Carcinoma | M0 | N0 | T1a | male | Alive | Squamous cell carcinoma, NOS | stage ia |
| TCGA-85-8276-01A | 62 | Lung Squamous Cell Carcinoma | M0 | N1 | T1b | male | Alive | Squamous cell carcinoma, NOS | stage iia |
| TCGA-85-8277-01A | 70 | Lung Squamous Cell Carcinoma | M0 | N1 | T3 | male | Dead | Squamous cell carcinoma, NOS | stage iiia |
| TCGA-85-8287-01A | 72 | Lung Squamous Cell Carcinoma | M0 | N0 | T1b | male | Dead | Squamous cell carcinoma, NOS | stage ia |
| TCGA-85-8288-01A | 70 | Lung Squamous Cell Carcinoma | M0 | N1 | T1b | male | Dead | Squamous cell carcinoma, NOS | stage iia |
| TCGA-85-8350-01A | 61 | Lung Squamous Cell Carcinoma | M0 | N0 | T1b | male | Alive | Squamous cell carcinoma, NOS | stage ia |
| TCGA-85-8351-01A | 72 | Lung Squamous Cell Carcinoma | M0 | N1 | T2a | male | Alive | Squamous cell carcinoma, NOS | stage iia |
| TCGA-85-8352-01A | 67 | Lung Squamous Cell Carcinoma | M0 | N1 | T3 | male | Dead | Squamous cell carcinoma, NOS | stage iiia |
| TCGA-85-8353-01A | 72 | Lung Squamous Cell Carcinoma | M0 | N1 | T3 | male | Dead | Squamous cell carcinoma, NOS | stage iiia |
| TCGA-85-8354-01A | 53 | Lung Squamous Cell Carcinoma | M0 | N0 | T2a | male | Alive | Squamous cell carcinoma, NOS | stage ib |
| TCGA-85-8355-01A | 63 | Lung Squamous Cell Carcinoma | M0 | N0 | T1a | male | Alive | Squamous cell carcinoma, NOS | stage ia |
| TCGA-85-8479-01A | 66 | Lung Squamous Cell Carcinoma | M0 | N0 | T1a | male | Alive | Papillary squamous cell carcinoma | stage ia |
| TCGA-85-8481-01A | 70 | Lung Squamous Cell Carcinoma | M0 | N0 | T3 | male | Dead | Squamous cell carcinoma, NOS | stage iib |
| TCGA-85-8580-01A | 52 | Lung Squamous Cell Carcinoma | M0 | N0 | T2a | female | Alive | Squamous cell carcinoma, NOS | stage ib |
| TCGA-85-8582-01A | 49 | Lung Squamous Cell Carcinoma | M0 | N0 | T1a | male | Alive | Squamous cell carcinoma, keratinizing, NOS | stage ia |

| | | | | | | | | | |
|---|---|---|---|---|---|---|---|---|---|
| TCGA-85-8584-01A | 71 | Lung Squamous Cell Carcinoma | M0 | N1 | T2a | male | Dead | Papillary squamous cell carcinoma | stage iia |
| TCGA-85-8664-01A | 73 | Lung Squamous Cell Carcinoma | M0 | N1 | T2b | male | Dead | Squamous cell carcinoma, NOS | stage iib |
| TCGA-85-8666-01A | 65 | Lung Squamous Cell Carcinoma | M0 | N0 | T2a | male | Dead | Squamous cell carcinoma, NOS | stage ib |
| TCGA-85-A4CL-01A | 65 | Lung Squamous Cell Carcinoma | M0 | N0 | T1b | male | Dead | Squamous cell carcinoma, NOS | stage ia |
| TCGA-85-A4CN-01A | 56 | Lung Squamous Cell Carcinoma | M0 | N1 | T2b | female | Alive | Squamous cell carcinoma, NOS | stage iib |
| TCGA-85-A4JB-01A | 74 | Lung Squamous Cell Carcinoma | M0 | N0 | T3 | male | Alive | Squamous cell carcinoma, NOS | stage iib |
| TCGA-85-A4JC-01A | 84 | Lung Squamous Cell Carcinoma | M0 | N0 | T2b | male | Dead | Squamous cell carcinoma, NOS | stage iia |
| TCGA-85-A4PA-01A | 61 | Lung Squamous Cell Carcinoma | M0 | N0 | T2a | male | Alive | Squamous cell carcinoma, large cell, nonkeratinizing, NOS | stage ib |
| TCGA-85-A4QQ-01A | 68 | Lung Squamous Cell Carcinoma | M0 | N0 | T2a | male | Dead | Squamous cell carcinoma, keratinizing, NOS | stage ib |
| TCGA-85-A4QR-01A | 67 | Lung Squamous Cell Carcinoma | M0 | N0 | T2a | male | Alive | Squamous cell carcinoma, NOS | stage ib |
| TCGA-85-A50M-01A | 47 | Lung Squamous Cell Carcinoma | M0 | N0 | T2b | male | Dead | Squamous cell carcinoma, NOS | stage iia |
| TCGA-85-A50Z-01A | 57 | Lung Squamous Cell Carcinoma | M0 | N0 | T2b | male | Alive | Squamous cell carcinoma, NOS | stage iia |
| TCGA-85-A510-01A | 74 | Lung Squamous Cell Carcinoma | M0 | N1 | T2b | female | Alive | Squamous cell carcinoma, NOS | stage iib |
| TCGA-85-A511-01A | 62 | Lung Squamous Cell Carcinoma | M0 | N1 | T2b | male | Dead | Squamous cell carcinoma, NOS | stage iib |
| TCGA-85-A512-01A | 46 | Lung Squamous Cell Carcinoma | M0 | N1 | T1b | male | Alive | Squamous cell carcinoma, NOS | stage iia |
| TCGA-85-A513-01A | 60 | Lung Squamous Cell Carcinoma | M0 | NX | T1a | female | Alive | Squamous cell carcinoma, NOS | stage ia |
| TCGA-85-A53L-01A | 63 | Lung Squamous Cell Carcinoma | M0 | N0 | T2b | male | Alive | Squamous cell carcinoma, NOS | stage iia |
| TCGA-85-A5B5-01A | 58 | Lung Squamous Cell Carcinoma | M0 | N0 | T1b | male | Alive | Squamous cell carcinoma, NOS | stage ia |
| TCGA-90-6837-01A | 64 | Lung Squamous Cell Carcinoma | MX | N0 | T3 | male | Alive | Squamous cell carcinoma, NOS | stage iib |
| TCGA-90-7766-01A | 66 | Lung Squamous Cell Carcinoma | MX | N0 | T1b | female | Alive | Squamous cell carcinoma, NOS | stage ia |
| TCGA-90-7767-01A | 56 | Lung Squamous Cell Carcinoma | MX | N1 | T2b | male | Alive | Squamous cell carcinoma, NOS | stage iib |

| | | | | | | | | | |
|---|---|---|---|---|---|---|---|---|---|
| TCGA-90-7769-01A | 55 | Lung Squamous Cell Carcinoma | MX | N1 | T2b | male | Alive | Squamous cell carcinoma, NOS | stage iib |
| TCGA-90-7964-01A | 70 | Lung Squamous Cell Carcinoma | MX | N0 | T2a | male | Alive | Squamous cell carcinoma, NOS | stage ib |
| TCGA-90-A4ED-01A | 69 | Lung Squamous Cell Carcinoma | MX | N0 | T2a | male | Alive | Squamous cell carcinoma, NOS | stage ib |
| TCGA-90-A4EE-01A | 53 | Lung Squamous Cell Carcinoma | MX | N1 | T2a | male | Alive | Squamous cell carcinoma, NOS | stage iia |
| TCGA-90-A59Q-01A | 61 | Lung Squamous Cell Carcinoma | MX | N1 | T2a | female | Dead | Squamous cell carcinoma, NOS | stage iia |
| TCGA-92-7340-01A | 45 | Lung Squamous Cell Carcinoma | MX | N1 | T2a | female | Alive | Squamous cell carcinoma, NOS | stage iia |
| TCGA-92-7341-01A | 71 | Lung Squamous Cell Carcinoma | MX | N0 | T2a | male | Alive | Squamous cell carcinoma, NOS | stage ib |
| TCGA-92-8063-01A | 52 | Lung Squamous Cell Carcinoma | MX | N2 | T2b | male | Alive | Squamous cell carcinoma, NOS | stage iiia |
| TCGA-92-8064-01A | 58 | Lung Squamous Cell Carcinoma | MX | N0 | T2b | male | Alive | Squamous cell carcinoma, NOS | not reported |
| TCGA-92-8065-01A | 74 | Lung Squamous Cell Carcinoma | MX | N0 | T3 | female | Alive | Squamous cell carcinoma, keratinizing, NOS | stage iib |
| TCGA-94-7033-01A | 73 | Lung Squamous Cell Carcinoma | MX | N0 | T2 | male | Alive | Squamous cell carcinoma, NOS | stage ib |
| TCGA-94-7557-01A | 73 | Lung Squamous Cell Carcinoma | M0 | N0 | T2 | male | Dead | Squamous cell carcinoma, NOS | stage ib |
| TCGA-94-7943-01A | 80 | Lung Squamous Cell Carcinoma | MX | NX | T1b | male | Alive | Squamous cell carcinoma, NOS | stage ia |
| TCGA-94-8035-01A | 64 | Lung Squamous Cell Carcinoma | MX | N0 | T3 | male | Alive | Squamous cell carcinoma, NOS | stage iib |
| TCGA-94-8490-01A | 70 | Lung Squamous Cell Carcinoma | M0 | N0 | T3 | male | Alive | Squamous cell carcinoma, NOS | stage iib |
| TCGA-94-8491-01A | 73 | Lung Squamous Cell Carcinoma | M0 | N1 | T2a | male | Alive | Squamous cell carcinoma, NOS | stage iia |
| TCGA-94-A4VJ-01A | 71 | Lung Squamous Cell Carcinoma | M0 | N0 | T1b | female | Alive | Squamous cell carcinoma, NOS | stage ia |
| TCGA-94-A5I4-01A | 61 | Lung Squamous Cell Carcinoma | MX | N1 | T2a | male | Alive | Squamous cell carcinoma, NOS | stage iia |
| TCGA-94-A5I6-01A | 62 | Lung Squamous Cell Carcinoma | M0 | N0 | T3 | male | Alive | Squamous cell carcinoma, NOS | stage iib |
| TCGA-96-7544-01A | 83 | Lung Squamous Cell Carcinoma | MX | N1 | T2 | male | Dead | Squamous cell carcinoma, NOS | stage iib |
| TCGA-96-7545-01A | 73 | Lung Squamous Cell Carcinoma | MX | N0 | T1 | male | Dead | Squamous cell carcinoma, NOS | stage ia |
| TCGA-96-8169-01A | 67 | Lung Squamous Cell Carcinoma | M0 | N0 | T1a | female | Alive | Squamous cell carcinoma, NOS | stage ia |
| TCGA-96-8170-01A | 75 | Lung Squamous Cell Carcinoma | M0 | N1 | T1a | female | Alive | Squamous cell carcinoma, NOS | stage iia |
| TCGA-96-A4JK-01A | 65 | Lung Squamous Cell Carcinoma | M0 | N1 | T2a | male | Alive | Squamous cell carcinoma, NOS | stage iia |

| | | | | | | | | | |
|---|---|---|---|---|---|---|---|---|---|
| TCGA-96-A4JL-01A | 78 | Lung Squamous Cell Carcinoma | M0 | N1 | T2a | female | Alive | Squamous cell carcinoma, NOS | stage iia |
| TCGA-98-7454-01A | 73 | Lung Squamous Cell Carcinoma | M0 | N0 | T2a | male | Alive | Squamous cell carcinoma, NOS | stage ib |
| TCGA-98-8020-01A | 56 | Lung Squamous Cell Carcinoma | M0 | N2 | T2 | female | Dead | Squamous cell carcinoma, NOS | stage iiia |
| TCGA-98-8021-01A | 75 | Lung Squamous Cell Carcinoma | M0 | N0 | T1a | female | Alive | Squamous cell carcinoma, NOS | stage ia |
| TCGA-98-8022-01A | 61 | Lung Squamous Cell Carcinoma | M0 | N0 | T1a | male | Dead | Squamous cell carcinoma, NOS | stage ia |
| TCGA-98-8023-01A | 70 | Lung Squamous Cell Carcinoma | M0 | N1 | T3 | male | Alive | Squamous cell carcinoma, NOS | stage iiia |
| TCGA-98-A538-01A | 67 | Lung Squamous Cell Carcinoma | M0 | N0 | T3 | male | Alive | Squamous cell carcinoma, NOS | stage iib |
| TCGA-98-A539-01A | 63 | Lung Squamous Cell Carcinoma | M0 | N0 | T3 | male | Alive | Squamous cell carcinoma, NOS | stage iib |
| TCGA-98-A53A-01A | 70 | Lung Squamous Cell Carcinoma | M0 | N0 | T2a | male | Dead | Squamous cell carcinoma, NOS | stage ib |
| TCGA-98-A53B-01A | 69 | Lung Squamous Cell Carcinoma | M0 | N0 | T2a | male | Dead | Squamous cell carcinoma, NOS | stage ib |
| TCGA-98-A53C-01A | 77 | Lung Squamous Cell Carcinoma | M0 | N0 | T1a | female | Alive | Squamous cell carcinoma, NOS | stage ia |
| TCGA-98-A53D-01A | 68 | Lung Squamous Cell Carcinoma | M0 | N0 | T3 | male | Dead | Squamous cell carcinoma, keratinizing, NOS | stage iib |
| TCGA-98-A53H-01A | 76 | Lung Squamous Cell Carcinoma | M0 | N0 | T1a | female | Alive | Squamous cell carcinoma, NOS | stage ia |
| TCGA-98-A53I-01A | 64 | Lung Squamous Cell Carcinoma | M0 | N1 | T2a | male | Alive | Squamous cell carcinoma, NOS | stage iia |
| TCGA-98-A53J-01A | 77 | Lung Squamous Cell Carcinoma | M0 | N0 | T2a | male | Alive | Squamous cell carcinoma, NOS | stage ib |
| TCGA-J1-A4AH-01A | 70 | Lung Squamous Cell Carcinoma | MX | N0 | T2b | male | Alive | Squamous cell carcinoma, NOS | stage iia |
| TCGA-L3-A4E7-01A | 71 | Lung Squamous Cell Carcinoma | M0 | N0 | T2a | male | Alive | Squamous cell carcinoma, NOS | stage ib |
| TCGA-L3-A524-01A | 45 | Lung Squamous Cell Carcinoma | M0 | N0 | T3 | female | Dead | Squamous cell carcinoma, NOS | stage iib |
| TCGA-LA-A446-01A | 68 | Lung Squamous Cell Carcinoma | MX | N0 | T1b | male | Alive | Squamous cell carcinoma, NOS | stage ia |
| TCGA-LA-A7SW-01A | 71 | Lung Squamous Cell Carcinoma | MX | N1 | T3 | male | Dead | Squamous cell carcinoma, NOS | stage iiia |
| TCGA-MF-A522-01A | 54 | Lung Squamous Cell Carcinoma | MX | N0 | T2a | male | Dead | Squamous cell carcinoma, keratinizing, NOS | stage ib |
| TCGA-NC-A5HD-01A | 79 | Lung Squamous Cell Carcinoma | M0 | N0 | T3 | male | Dead | Squamous cell carcinoma, NOS | stage iib |
| TCGA-NC-A5HE-01A | 60 | Lung Squamous Cell Carcinoma | M0 | N1 | T2 | male | Alive | Squamous cell carcinoma, NOS | stage iib |

| | | | | | | | | | |
|---|---|---|---|---|---|---|---|---|---|
| TCGA-NC-A5HF-01A | 74 | Lung Squamous Cell Carcinoma | MX | N0 | T4 | male | Dead | Squamous cell carcinoma, keratinizing, NOS | stage iiib |
| TCGA-NC-A5HG-01A | 59 | Lung Squamous Cell Carcinoma | M0 | N2 | T2 | male | Alive | Basaloid squamous cell carcinoma | stage iiia |
| TCGA-NC-A5HH-01A | 53 | Lung Squamous Cell Carcinoma | M0 | N0 | T1 | male | Alive | Basaloid squamous cell carcinoma | stage ia |
| TCGA-NC-A5HI-01A | 68 | Lung Squamous Cell Carcinoma | M0 | N0 | T2 | female | Alive | Squamous cell carcinoma, NOS | stage ib |
| TCGA-NC-A5HJ-01A | 59 | Lung Squamous Cell Carcinoma | M0 | N0 | T3 | male | Dead | Squamous cell carcinoma, NOS | stage iib |
| TCGA-NC-A5HK-01A | 58 | Lung Squamous Cell Carcinoma | M0 | N0 | T3 | female | Alive | Squamous cell carcinoma, NOS | stage iib |
| TCGA-NC-A5HL-01A | 73 | Lung Squamous Cell Carcinoma | M0 | N0 | T2b | male | Dead | Squamous cell carcinoma, NOS | stage iia |
| TCGA-NC-A5HM-01A | 76 | Lung Squamous Cell Carcinoma | M0 | N0 | T2a | male | Alive | Squamous cell carcinoma, NOS | stage ib |
| TCGA-NC-A5HN-01A | 77 | Lung Squamous Cell Carcinoma | M0 | N1 | T2a | male | Alive | Squamous cell carcinoma, NOS | stage iia |
| TCGA-NC-A5HO-01A | 70 | Lung Squamous Cell Carcinoma | M0 | N1 | T3 | female | Alive | Squamous cell carcinoma, keratinizing, NOS | stage iiia |
| TCGA-NC-A5HP-01A | 69 | Lung Squamous Cell Carcinoma | M1b | N0 | T2a | male | Dead | Squamous cell carcinoma, keratinizing, NOS | stage iv |
| TCGA-NC-A5HQ-01A | 70 | Lung Squamous Cell Carcinoma | M0 | N2 | T3 | male | Dead | Squamous cell carcinoma, keratinizing, NOS | stage iiia |
| TCGA-NC-A5HR-01A | 75 | Lung Squamous Cell Carcinoma | M0 | N1 | T2a | female | Alive | Basaloid squamous cell carcinoma | stage iia |
| TCGA-NC-A5HT-01A | 69 | Lung Squamous Cell Carcinoma | M0 | N1 | T3 | male | Alive | Squamous cell carcinoma, NOS | stage iiia |
| TCGA-NK-A5CR-01A | 77 | Lung Squamous Cell Carcinoma | MX | N0 | T2 | male | Alive | Squamous cell carcinoma, NOS | stage ib |
| TCGA-NK-A5CT-01A | 70 | Lung Squamous Cell Carcinoma | M0 | N0 | T1 | male | Alive | Squamous cell carcinoma, NOS | stage ia |
| TCGA-NK-A5CX-01A | 73 | Lung Squamous Cell Carcinoma | MX | N0 | T2b | male | Alive | Squamous cell carcinoma, NOS | stage iia |
| TCGA-NK-A5D1-01A | 57 | Lung Squamous Cell Carcinoma | M0 | N1 | T2a | male | Alive | Squamous cell carcinoma, NOS | stage iia |

| | | | | | | | | | |
|---|---|---|---|---|---|---|---|---|---|
| TCGA-NK-A7XE-01A | 66 | Lung Squamous Cell Carcinoma | M0 | N2 | T4 | male | Alive | Squamous cell carcinoma, NOS | stage iiib |
| TCGA-02-A52N-01A | 78 | Lung Squamous Cell Carcinoma | MX | N0 | T2 | male | Dead | Squamous cell carcinoma, NOS | stage i |
| TCGA-02-A52Q-01A | 44 | Lung Squamous Cell Carcinoma | MX | N1 | T3 | female | Dead | Squamous cell carcinoma, NOS | stage iii |
| TCGA-02-A52S-01A | 57 | Lung Squamous Cell Carcinoma | MX | N2 | T4 | female | Dead | Squamous cell carcinoma, NOS | stage iii |
| TCGA-02-A52V-01A | 75 | Lung Squamous Cell Carcinoma | MX | N0 | T3 | female | Dead | Squamous cell carcinoma, NOS | stage ii |
| TCGA-02-A52W-01A | 63 | Lung Squamous Cell Carcinoma | MX | N0 | T2 | male | Dead | Squamous cell carcinoma, NOS | stage i |
| TCGA-02-A5IB-01A | 71 | Lung Squamous Cell Carcinoma | MX | N1 | T3 | female | Dead | Squamous cell carcinoma, NOS | stage iii |
| TCGA-XC-AA0X-01A | 77 | Lung Squamous Cell Carcinoma | M0 | N0 | T1a | female | Dead | Squamous cell carcinoma, NOS | stage ia |

**supplementary table 3**

| ID | futime | fustat | Age | Gender | Stage | riskScore | nomoRisk |
|---|---|---|---|---|---|---|---|
| TCGA-21-1075 | 5.846575342 | 0 | 57 | MALE | Stage II | -0.929354805 | 0.759059304 |
| TCGA-63-A5MW | 4.490410959 | 0 | 76 | MALE | Stage I | -0.883818223 | 0.877473618 |
| TCGA-85-8070 | 2.630136986 | 0 | 71 | MALE | Stage I | -0.88093456 | 0.810041878 |
| TCGA-NK-A5CR | 6.964383562 | 0 | 77 | MALE | Stage I | -0.849031227 | 0.891619149 |
| TCGA-63-A5MJ | 4.997260274 | 0 | 54 | MALE | Stage II | -0.800301477 | 0.723502024 |
| TCGA-85-A512 | 1.273972603 | 0 | 46 | MALE | Stage II | -0.787026807 | 0.636615483 |
| TCGA-22-5478 | 0.065753425 | 1 | 79 | MALE | Stage I | -0.72791317 | 0.920597996 |
| TCGA-85-A5B5 | 0.304109589 | 0 | 58 | MALE | Stage I | -0.711694619 | 0.657988529 |
| TCGA-85-8277 | 0.84109589 | 1 | 70 | MALE | Stage III | -0.702272648 | 1.155512361 |
| TCGA-56-7223 | 1.210958904 | 1 | 66 | MALE | Stage III | -0.689740777 | 1.08391024 |
| TCGA-58-A46N | 2.493150685 | 0 | 52 | MALE | Stage I | -0.680567597 | 0.597786919 |
| TCGA-NC-A5HF | 0.378082192 | 1 | 74 | MALE | Stage III | -0.68056516 | 1.231844454 |
| TCGA-56-8629 | 1.317808219 | 0 | 63 | MALE | Stage II | -0.676131614 | 0.835502249 |
| TCGA-J1-A4AH | 1.591780822 | 0 | 70 | MALE | Stage II | -0.661451698 | 0.934468911 |
| TCGA-63-A5MB | 8.556164384 | 0 | 62 | MALE | Stage I | -0.656501499 | 0.701454651 |
| TCGA-85-A4CL | 2.523287671 | 1 | 65 | MALE | Stage I | -0.639447325 | 0.735928389 |
| TCGA-58-8391 | 5.936986301 | 0 | 57 | FEMALE | Stage III | -0.639133208 | 0.724618147 |
| TCGA-77-6843 | 6.093150685 | 1 | 74 | MALE | Stage II | -0.631332049 | 0.996199075 |
| TCGA-85-8354 | 2.726027397 | 0 | 53 | MALE | Stage I | -0.622799027 | 0.607423691 |
| TCGA-33-AAS8 | 3.052054795 | 1 | 59 | FEMALE | Stage I | -0.619269487 | 0.516163632 |
| TCGA-77-A5G8 | 5.161643836 | 0 | 70 | MALE | Stage II | -0.617521198 | 0.934468911 |
| TCGA-NC-A5HK | 0.350684932 | 0 | 58 | FEMALE | Stage II | -0.616670407 | 0.595449297 |
| TCGA-46-6026 | 1.15890411 | 0 | 81 | MALE | Stage II | -0.614620023 | 1.11420055 |
| TCGA-60-2703 | 8.068493151 | 1 | 73 | MALE | Stage II | -0.613345051 | 0.980394384 |
| TCGA-37-4135 | 0.567123288 | 0 | 68 | MALE | Stage I | -0.606810166 | 0.772096376 |
| TCGA-22-4601 | 2.895890411 | 1 | 73 | FEMALE | Stage III | -0.598187642 | 0.935910486 |
| TCGA-90-7769 | 0.980821918 | 0 | 55 | MALE | Stage II | -0.597697179 | 0.735165418 |
| TCGA-66-2766 | 0.084931507 | 0 | 54 | MALE | Stage III | -0.589590785 | 0.894642425 |
| TCGA-58-A46K | 2.863013699 | 1 | 59 | MALE | Stage III | -0.577547734 | 0.969116716 |
| TCGA-33-A5GW | 0.024657534 | 0 | 67 | MALE | Stage II | -0.575078198 | 0.890694765 |
| TCGA-77-6845 | 1.939726027 | 1 | 69 | MALE | Stage II | -0.571729463 | 0.919643569 |
| TCGA-37-4129 | 0.663013699 | 0 | 52 | FEMALE | Stage I | -0.565023704 | 0.461498365 |
| TCGA-LA-A446 | 1.098630137 | 0 | 68 | MALE | Stage I | -0.561136229 | 0.772096376 |
| TCGA-60-2711 | 3.452054795 | 0 | 64 | FEMALE | Stage I | -0.558971198 | 0.559131548 |
| TCGA-43-A474 | 0.967123288 | 0 | 66 | MALE | Stage II | -0.557127333 | 0.8765639 |
| TCGA-56-A5DR | 0.010958904 | 0 | 81 | MALE | Stage I | -0.555908071 | 0.950518696 |
| TCGA-77-8140 | 0.961643836 | 1 | 66 | FEMALE | Stage II | -0.55418607 | 0.676717396 |
| TCGA-L3-A524 | 1.342465753 | 1 | 45 | FEMALE | Stage II | -0.553610972 | 0.483677224 |
| TCGA-NC-A5HR | 3.408219178 | 0 | 75 | FEMALE | Stage II | -0.553451467 | 0.781475223 |
| TCGA-NC-A5HL | 0.24109589 | 1 | 73 | MALE | Stage II | -0.55055307 | 0.980394384 |
| TCGA-77-A5GA | 3.506849315 | 0 | 76 | MALE | Stage I | -0.549781885 | 0.877473618 |

| | | | | | | | |
|---|---|---|---|---|---|---|---|
| TCGA-39-5036 | 5.931506849 | 0 | 73 | MALE | Stage I | -0.548108361 | 0.836369352 |
| TCGA-21-1082 | 9.983561644 | 0 | 61 | MALE | Stage I | -0.541209079 | 0.690326078 |
| TCGA-NC-A5HD | 0.005479452 | 1 | 79 | MALE | Stage II | -0.535128811 | 1.079127426 |
| TCGA-94-A5I4 | 1.345205479 | 0 | 61 | MALE | Stage II | -0.530762462 | 0.80920207 |
| TCGA-58-8387 | 1.104109589 | 1 | 60 | MALE | Stage II | -0.527647544 | 0.796364085 |
| TCGA-21-1083 | 3.602739726 | 1 | 75 | MALE | Stage I | -0.525509493 | 0.863552505 |
| TCGA-37-A5EL | 3.131506849 | 1 | 53 | MALE | Stage II | -0.523121087 | 0.71202367 |
| TCGA-51-4081 | 2.495890411 | 0 | 55 | MALE | Stage II | -0.523053385 | 0.735165418 |
| TCGA-77-8150 | 4.534246575 | 1 | 64 | MALE | Stage III | -0.517072032 | 1.049790602 |
| TCGA-NC-A5HG | 5.378082192 | 0 | 59 | MALE | Stage III | -0.506726902 | 0.969116716 |
| TCGA-85-A4QR | 1.643835616 | 0 | 67 | MALE | Stage I | -0.498182965 | 0.759847073 |
| TCGA-22-5477 | 3.687671233 | 1 | 65 | MALE | Stage I | -0.49764437 | 0.735928389 |
| TCGA-NC-A5HH | 0.101369863 | 0 | 53 | MALE | Stage I | -0.496897362 | 0.607423691 |
| TCGA-85-8479 | 1.282191781 | 0 | 66 | MALE | Stage I | -0.491232327 | 0.747792105 |
| TCGA-60-2726 | 0.980821918 | 1 | 56 | MALE | Stage II | -0.48769314 | 0.747016834 |
| TCGA-21-1080 | 10.20273973 | 0 | 66 | MALE | Stage I | -0.485526493 | 0.747792105 |
| TCGA-56-7582 | 1.646575342 | 0 | 83 | MALE | Stage I | -0.484697543 | 0.98141186 |
| TCGA-66-2781 | 0.331506849 | 0 | 67 | MALE | Stage I | -0.482296038 | 0.759847073 |
| TCGA-66-2792 | 2.501369863 | 0 | 58 | MALE | Stage II | -0.480120707 | 0.771295908 |
| TCGA-94-8035 | 0.334246575 | 0 | 64 | MALE | Stage II | -0.474950227 | 0.84897117 |
| TCGA-18-3408 | 6.312328767 | 1 | 77 | FEMALE | Stage I | -0.465444516 | 0.688340221 |
| TCGA-39-5027 | 8.515068493 | 0 | 73 | MALE | Stage I | -0.464438581 | 0.836369352 |
| TCGA-43-3394 | 3.260273973 | 1 | 52 | MALE | Stage I | -0.460620357 | 0.597786919 |
| TCGA-56-7221 | 1.665753425 | 0 | 79 | MALE | Stage I | -0.454926634 | 0.920597996 |
| TCGA-60-2722 | 2.487671233 | 0 | 66 | MALE | Stage II | -0.452676659 | 0.8765639 |
| TCGA-18-5592 | 4.161643836 | 0 | 57 | MALE | Stage II | -0.450910569 | 0.759059304 |
| TCGA-33-AASD | 6.161643836 | 1 | 83 | MALE | Stage I | -0.449578596 | 0.98141186 |
| TCGA-37-4133 | 0.652054795 | 0 | 63 | MALE | Stage III | -0.445865778 | 1.033135683 |
| TCGA-33-4586 | 1.17260274 | 1 | 57 | MALE | Stage III | -0.442568134 | 0.938610583 |
| TCGA-58-8390 | 2.495890411 | 0 | 70 | MALE | Stage II | -0.440289743 | 0.934468911 |
| TCGA-96-A4JL | 2.306849315 | 0 | 78 | FEMALE | Stage II | -0.43806306 | 0.819881658 |
| TCGA-43-7657 | 0.646575342 | 0 | 68 | FEMALE | Stage I | -0.436848621 | 0.596067268 |
| TCGA-56-A4BX | 1.109589041 | 0 | 70 | MALE | Stage II | -0.435660128 | 0.934468911 |
| TCGA-85-6798 | 0.534246575 | 1 | 57 | MALE | Stage III | -0.43495543 | 0.938610583 |
| TCGA-33-AASJ | 9.863013699 | 1 | 60 | MALE | Stage I | -0.428070908 | 0.67937406 |
| TCGA-98-A53J | 1.726027397 | 0 | 77 | MALE | Stage I | -0.4280166 | 0.891619149 |
| TCGA-98-A53A | 1.512328767 | 1 | 70 | MALE | Stage I | -0.418752492 | 0.79719057 |
| TCGA-56-A4BY | 1.487671233 | 1 | 66 | MALE | Stage I | -0.416359569 | 0.747792105 |
| TCGA-43-8116 | 0.980821918 | 0 | 73 | MALE | Stage I | -0.411421261 | 0.836369352 |
| TCGA-94-8490 | 0.419178082 | 0 | 70 | MALE | Stage II | -0.411133093 | 0.934468911 |
| TCGA-39-5019 | 9.279452055 | 0 | 70 | MALE | Stage I | -0.410710438 | 0.79719057 |
| TCGA-21-A5DI | 2.682191781 | 0 | 77 | MALE | Stage I | -0.408959638 | 0.891619149 |
| TCGA-63-A5MY | 2.882191781 | 0 | 63 | MALE | Stage I | -0.406825066 | 0.712762625 |

| | | | | | | | |
|---|---|---|---|---|---|---|---|
| TCGA-77-7142 | 6.101369863 | 0 | 59 | FEMALE | Stage I | -0.398599181 | 0.516163632 |
| TCGA-85-8664 | 1.506849315 | 1 | 73 | MALE | Stage II | -0.397485727 | 0.980394384 |
| TCGA-85-7710 | 0.115068493 | 0 | 59 | FEMALE | Stage I | -0.392335983 | 0.516163632 |
| TCGA-77-8133 | 4.493150685 | 1 | 74 | MALE | Stage II | -0.390340257 | 0.996199075 |
| TCGA-77-A5G3 | 12.52054795 | 0 | 63 | MALE | Stage II | -0.382643985 | 0.835502249 |
| TCGA-43-3920 | 2.75890411 | 0 | 71 | MALE | Stage I | -0.379970151 | 0.810041878 |
| TCGA-77-8153 | 5.457534247 | 0 | 77 | FEMALE | Stage I | -0.37972006 | 0.688340221 |
| TCGA-NK-A5CT | 5.471232877 | 0 | 70 | MALE | Stage I | -0.379678385 | 0.79719057 |
| TCGA-77-7140 | 1.731506849 | 1 | 69 | FEMALE | Stage II | -0.378842898 | 0.709975395 |
| TCGA-18-4083 | 0.515068493 | 1 | 63 | MALE | Stage II | -0.377259148 | 0.835502249 |
| TCGA-33-A4WN | 0.391780822 | 1 | 60 | MALE | Stage I | -0.376051026 | 0.67937406 |
| TCGA-18-4721 | 12.86027397 | 0 | 74 | MALE | Stage I | -0.375438495 | 0.849852252 |
| TCGA-85-A4CN | 2.843835616 | 0 | 56 | FEMALE | Stage II | -0.375373119 | 0.576705574 |
| TCGA-98-A538 | 2.263013699 | 0 | 67 | MALE | Stage II | -0.375297908 | 0.890694765 |
| TCGA-63-A5ML | 3.797260274 | 0 | 68 | MALE | Stage I | -0.374714163 | 0.772096376 |
| TCGA-77-A5GF | 2.301369863 | 1 | 70 | MALE | Stage II | -0.374559206 | 0.934468911 |
| TCGA-22-5483 | 1.569863014 | 1 | 74 | MALE | Stage II | -0.373157419 | 0.996199075 |
| TCGA-85-7697 | 2.912328767 | 0 | 49 | MALE | Stage II | -0.373139716 | 0.667902632 |
| TCGA-22-4613 | 0.980821918 | 1 | 73 | FEMALE | Stage I | -0.371153083 | 0.645686743 |
| TCGA-21-1081 | 0.778082192 | 1 | 69 | MALE | Stage II | -0.369513623 | 0.919643569 |
| TCGA-77-8128 | 3.150684932 | 1 | 60 | MALE | Stage III | -0.367976647 | 0.984739603 |
| TCGA-34-A5IX | 2.824657534 | 0 | 80 | MALE | Stage II | -0.367520513 | 1.096523767 |
| TCGA-66-2737 | 0.167123288 | 0 | 72 | MALE | Stage II | -0.365875081 | 0.964840435 |
| TCGA-85-A510 | 1.320547945 | 0 | 74 | FEMALE | Stage II | -0.365089646 | 0.769077125 |
| TCGA-18-5595 | 2.265753425 | 1 | 50 | MALE | Stage I | -0.364359344 | 0.578969611 |
| TCGA-90-A4ED | 1.684931507 | 0 | 69 | MALE | Stage I | -0.363041073 | 0.784543148 |
| TCGA-77-7139 | 11.6739726 | 0 | 56 | MALE | Stage II | -0.362659433 | 0.747016834 |
| TCGA-NC-A5HP | 2.109589041 | 1 | 69 | MALE | Stage IV | -0.362633453 | 2.529888364 |
| TCGA-85-A4QQ | 2.539726027 | 1 | 68 | MALE | Stage I | -0.362508541 | 0.772096376 |
| TCGA-63-A5MH | 5.550684932 | 0 | 68 | MALE | Stage I | -0.360987135 | 0.772096376 |
| TCGA-94-A5I6 | 1.473972603 | 0 | 62 | MALE | Stage II | -0.359104059 | 0.822247012 |
| TCGA-66-2757 | 3.665753425 | 1 | 65 | FEMALE | Stage I | -0.356551975 | 0.568145167 |
| TCGA-22-A5C4 | 1.838356164 | 0 | 70 | MALE | Stage II | -0.355584939 | 0.934468911 |
| TCGA-21-1078 | 1.298630137 | 1 | 77 | MALE | Stage I | -0.352574932 | 0.891619149 |
| TCGA-66-2787 | 3.334246575 | 0 | 57 | MALE | Stage I | -0.351699069 | 0.647549546 |
| TCGA-22-4591 | 1.706849315 | 1 | 80 | MALE | Stage III | -0.348639669 | 1.355900396 |
| TCGA-58-A46M | 2.936986301 | 0 | 61 | MALE | Stage II | -0.34732124 | 0.80920207 |
| TCGA-21-1071 | 3.906849315 | 1 | 67 | MALE | Stage I | -0.346989047 | 0.759847073 |
| TCGA-34-5231 | 5.435616438 | 1 | 72 | MALE | Stage I | -0.3442636 | 0.823100359 |
| TCGA-77-8154 | 5.043835616 | 0 | 67 | MALE | Stage I | -0.343733352 | 0.759847073 |
| TCGA-77-A5G6 | 1.857534247 | 1 | 66 | MALE | Stage III | -0.343577834 | 1.08391024 |
| TCGA-33-AASL | 2.263013699 | 1 | 57 | FEMALE | Stage I | -0.34235856 | 0.499915685 |
| TCGA-33-4582 | 8.62739726 | 1 | 55 | MALE | Stage I | -0.340608767 | 0.627165796 |

| | | | | | | | |
|---|---|---|---|---|---|---|---|
| TCGA-43-5670 | 2.326027397 | 0 | 70 | MALE | Stage II | -0.340422439 | 0.934468911 |
| TCGA-22-4593 | 2.923287671 | 1 | 77 | MALE | Stage II | -0.335510996 | 1.045158343 |
| TCGA-68-7755 | 0.22739726 | 0 | 60 | FEMALE | Stage II | -0.335061228 | 0.614802218 |
| TCGA-18-3414 | 1.961643836 | 1 | 73 | MALE | Stage IV | -0.333647008 | 2.697010482 |
| TCGA-56-8305 | 0.287671233 | 0 | 72 | MALE | Stage I | -0.333459395 | 0.823100359 |
| TCGA-77-7141 | 0.04109589 | 0 | 64 | MALE | Stage I | -0.332171457 | 0.724252891 |
| TCGA-77-6842 | 2.463013699 | 1 | 79 | MALE | Stage II | -0.330294342 | 1.079127426 |
| TCGA-90-7766 | 0.791780822 | 0 | 66 | FEMALE | Stage I | -0.329752175 | 0.577304092 |
| TCGA-02-A5IB | 0.931506849 | 1 | 71 | FEMALE | Stage III | -0.328194415 | 0.906449627 |
| TCGA-85-A53L | 1.032876712 | 0 | 63 | MALE | Stage II | -0.328024402 | 0.835502249 |
| TCGA-85-7696 | 3.043835616 | 0 | 64 | MALE | Stage I | -0.326251113 | 0.724252891 |
| TCGA-85-8052 | 2.010958904 | 0 | 53 | MALE | Stage II | -0.326188321 | 0.71202367 |
| TCGA-85-8072 | 2.553424658 | 0 | 60 | MALE | Stage I | -0.325662233 | 0.67937406 |
| TCGA-33-4566 | 14.48493151 | 1 | 40 | MALE | Stage I | -0.325564691 | 0.493403899 |
| TCGA-18-3415 | 7.679452055 | 1 | 77 | MALE | Stage I | -0.324015569 | 0.891619149 |
| TCGA-18-4086 | 0.232876712 | 1 | 64 | MALE | Stage I | -0.320940367 | 0.724252891 |
| TCGA-21-1072 | 8.263013699 | 0 | 75 | MALE | Stage I | -0.318015669 | 0.863552505 |
| TCGA-56-7730 | 0.542465753 | 1 | 73 | MALE | Stage II | -0.31789564 | 0.980394384 |
| TCGA-85-8350 | 1.871232877 | 0 | 61 | MALE | Stage I | -0.317122804 | 0.690326078 |
| TCGA-NC-A5HN | 4.106849315 | 0 | 77 | MALE | Stage II | -0.316975707 | 1.045158343 |
| TCGA-37-4141 | 0.032876712 | 0 | 65 | FEMALE | Stage I | -0.316175717 | 0.568145167 |
| TCGA-56-7580 | 2.534246575 | 0 | 84 | MALE | Stage I | -0.314730411 | 0.997232953 |
| TCGA-68-A59J | 1.22739726 | 0 | 74 | FEMALE | Stage I | -0.311293638 | 0.656095697 |
| TCGA-85-8288 | 1.101369863 | 1 | 70 | MALE | Stage II | -0.304171571 | 0.934468911 |
| TCGA-43-7656 | 1.632876712 | 0 | 71 | MALE | Stage I | -0.30300498 | 0.810041878 |
| TCGA-96-A4JK | 1.61369863 | 0 | 65 | MALE | Stage II | -0.302010637 | 0.86265722 |
| TCGA-34-5927 | 3.728767123 | 0 | 70 | FEMALE | Stage I | -0.299382509 | 0.615440273 |
| TCGA-56-8624 | 1.150684932 | 0 | 84 | MALE | Stage II | -0.296809838 | 1.168959127 |
| TCGA-77-7463 | 3.898630137 | 1 | 75 | MALE | Stage I | -0.294994199 | 0.863552505 |
| TCGA-22-5491 | 4.693150685 | 1 | 74 | MALE | Stage I | -0.292105756 | 0.849852252 |
| TCGA-85-A4JB | 2.580821918 | 0 | 74 | MALE | Stage II | -0.292009879 | 0.996199075 |
| TCGA-85-7698 | 2.608219178 | 0 | 48 | MALE | Stage I | -0.28964323 | 0.56074464 |
| TCGA-77-A5G7 | 0.493150685 | 1 | 63 | MALE | Stage I | -0.289325126 | 0.712762625 |
| TCGA-34-5929 | 0.41369863 | 1 | 78 | FEMALE | Stage I | -0.288345073 | 0.69943678 |
| TCGA-NK-A7XE | 0.035616438 | 0 | 66 | MALE | Stage III | -0.288259972 | 1.08391024 |
| TCGA-77-6844 | 6.257534247 | 1 | 74 | MALE | Stage III | -0.287670713 | 1.231844454 |
| TCGA-33-4532 | 10.75068493 | 1 | 68 | MALE | Stage I | -0.286468032 | 0.772096376 |
| TCGA-63-A5MV | 3.01369863 | 0 | 69 | MALE | Stage II | -0.284827326 | 0.919643569 |
| TCGA-98-A53I | 1.547945205 | 0 | 64 | MALE | Stage II | -0.28267441 | 0.84897117 |
| TCGA-02-A52S | 1.060273973 | 1 | 57 | FEMALE | Stage III | -0.281813446 | 0.724618147 |
| TCGA-52-7809 | 0.454794521 | 1 | 74 | MALE | Stage I | -0.278213853 | 0.849852252 |
| TCGA-34-8456 | 2.202739726 | 0 | 71 | FEMALE | Stage II | -0.277261819 | 0.733050571 |
| TCGA-66-2758 | 1.750684932 | 0 | 71 | MALE | Stage I | -0.277193301 | 0.810041878 |

| | | | | | | | |
|---|---|---|---|---|---|---|---|
| TCGA-22-5479 | 7.191780822 | 1 | 64 | MALE | Stage I | -0.27300082 | 0.724252891 |
| TCGA-77-7465 | 2.712328767 | 0 | 58 | MALE | Stage II | -0.271047718 | 0.771295908 |
| TCGA-39-5016 | 10.54794521 | 0 | 44 | MALE | Stage II | -0.270502228 | 0.616575919 |
| TCGA-77-8009 | 4.643835616 | 1 | 68 | MALE | Stage II | -0.270087428 | 0.905053431 |
| TCGA-56-A62T | 1.205479452 | 0 | 78 | MALE | Stage II | -0.26913284 | 1.062007077 |
| TCGA-02-A52V | 3.657534247 | 1 | 75 | FEMALE | Stage II | -0.268148851 | 0.781475223 |
| TCGA-34-7107 | 0.093150685 | 1 | 70 | MALE | Stage II | -0.261395621 | 0.934468911 |
| TCGA-85-7844 | 2.495890411 | 0 | 71 | MALE | Stage I | -0.261225668 | 0.810041878 |
| TCGA-85-7699 | 2.742465753 | 1 | 73 | MALE | Stage III | -0.260032172 | 1.212301251 |
| TCGA-94-8491 | 2.219178082 | 0 | 73 | MALE | Stage II | -0.259207206 | 0.980394384 |
| TCGA-77-8146 | 8.736986301 | 0 | 72 | MALE | Stage I | -0.258657931 | 0.823100359 |
| TCGA-37-4130 | 0.676712329 | 0 | 56 | MALE | Stage I | -0.258034249 | 0.637276177 |
| TCGA-43-6770 | 1.789041096 | 0 | 59 | FEMALE | Stage I | -0.256663329 | 0.516163632 |
| TCGA-22-5471 | 5.054794521 | 0 | 75 | MALE | Stage I | -0.253147596 | 0.863552505 |
| TCGA-63-A5MM | 1.249315068 | 1 | 69 | FEMALE | Stage II | -0.250608301 | 0.709975395 |
| TCGA-NC-A5H0 | 3.660273973 | 0 | 70 | FEMALE | Stage III | -0.249622746 | 0.892068811 |
| TCGA-60-2716 | 4.04109589 | 0 | 39 | MALE | Stage II | -0.247566797 | 0.56919354 |
| TCGA-94-7943 | 1.531506849 | 0 | 80 | MALE | Stage I | -0.24289098 | 0.935438724 |
| TCGA-02-A52W | 0.715068493 | 1 | 63 | MALE | Stage I | -0.23978394 | 0.712762625 |
| TCGA-98-A539 | 0.473972603 | 0 | 63 | MALE | Stage II | -0.239071883 | 0.835502249 |
| TCGA-51-4080 | 0.032876712 | 1 | 65 | MALE | Stage III | -0.238754324 | 1.066714012 |
| TCGA-33-4538 | 8.161643836 | 1 | 66 | MALE | Stage III | -0.234069548 | 1.08391024 |
| TCGA-66-2727 | 1.41369863 | 1 | 55 | FEMALE | Stage I | -0.23310853 | 0.484179196 |
| TCGA-22-1012 | 1.175342466 | 1 | 80 | FEMALE | Stage I | -0.232974282 | 0.722169436 |
| TCGA-63-A5MP | 2.106849315 | 0 | 56 | MALE | Stage II | -0.229440968 | 0.747016834 |
| TCGA-94-7033 | 1.753424658 | 0 | 73 | MALE | Stage I | -0.228510374 | 0.836369352 |
| TCGA-98-8021 | 2.567123288 | 0 | 75 | FEMALE | Stage I | -0.227851193 | 0.66667245 |
| TCGA-18-3412 | 0.945205479 | 1 | 52 | MALE | Stage I | -0.226400559 | 0.597786919 |
| TCGA-56-7823 | 2.769863014 | 0 | 58 | FEMALE | Stage II | -0.2250941 | 0.595449297 |
| TCGA-52-7810 | 2.528767123 | 0 | 60 | FEMALE | Stage II | -0.22491535 | 0.614802218 |
| TCGA-18-3421 | 7.246575342 | 0 | 65 | MALE | Stage I | -0.224886816 | 0.735928389 |
| TCGA-56-8307 | 2.24109589 | 0 | 55 | FEMALE | Stage II | -0.223540474 | 0.567556144 |
| TCGA-63-A5MG | 5.884931507 | 0 | 68 | MALE | Stage I | -0.221817109 | 0.772096376 |
| TCGA-NC-A5HM | 3.320547945 | 0 | 76 | MALE | Stage I | -0.221724315 | 0.877473618 |
| TCGA-66-2756 | 0.082191781 | 0 | 68 | MALE | Stage III | -0.216474836 | 1.119138812 |
| TCGA-21-5786 | 2.82739726 | 0 | 64 | MALE | Stage I | -0.215499247 | 0.724252891 |
| TCGA-60-2708 | 6.704109589 | 0 | 64 | FEMALE | Stage II | -0.214781288 | 0.655415491 |
| TCGA-60-2721 | 2.693150685 | 0 | 73 | MALE | Stage I | -0.211739326 | 0.836369352 |
| TCGA-MF-A522 | 0.98630137 | 1 | 54 | MALE | Stage I | -0.211701773 | 0.617215815 |
| TCGA-56-8308 | 1.416438356 | 0 | 79 | MALE | Stage II | -0.21133409 | 1.079127426 |
| TCGA-21-5783 | 7.342465753 | 1 | 76 | MALE | Stage I | -0.21096335 | 0.877473618 |
| TCGA-70-6723 | 1.02739726 | 0 | 65 | MALE | Stage II | -0.208478069 | 0.86265722 |
| TCGA-90-7767 | 0.243835616 | 0 | 56 | MALE | Stage II | -0.206871728 | 0.747016834 |

| | | | | | | | |
|---|---|---|---|---|---|---|---|
| TCGA-77-8139 | 8.673972603 | 0 | 72 | MALE | Stage II | -0.206790089 | 0.964840435 |
| TCGA-56-8504 | 1.397260274 | 0 | 74 | MALE | Stage I | -0.204335194 | 0.849852252 |
| TCGA-33-AASB | 0.578082192 | 1 | 66 | MALE | Stage I | -0.203758625 | 0.747792105 |
| TCGA-77-8148 | 5.542465753 | 0 | 68 | MALE | Stage III | -0.201461938 | 1.119138812 |
| TCGA-56-8622 | 0.150684932 | 0 | 68 | MALE | Stage I | -0.201453067 | 0.772096376 |
| TCGA-34-2600 | 5.134246575 | 1 | 76 | FEMALE | Stage I | -0.200984571 | 0.677419708 |
| TCGA-66-2742 | 1.756164384 | 0 | 70 | MALE | Stage IV | -0.197755762 | 2.570672057 |
| TCGA-46-3765 | 1.109589041 | 0 | 59 | FEMALE | Stage I | -0.197394984 | 0.516163632 |
| TCGA-34-5240 | 4.221917808 | 0 | 73 | FEMALE | Stage II | -0.194364577 | 0.756875722 |
| TCGA-66-2754 | 0.167123288 | 0 | 67 | MALE | Stage III | -0.192472551 | 1.101383683 |
| TCGA-56-A5DS | 0.021917808 | 0 | 72 | FEMALE | Stage I | -0.192385804 | 0.635442928 |
| TCGA-21-1070 | 9.961643836 | 0 | 60 | FEMALE | Stage III | -0.190055745 | 0.76023028 |
| TCGA-33-AASI | 3.682191781 | 1 | 65 | FEMALE | Stage II | -0.189579152 | 0.665981279 |
| TCGA-37-A5EN | 1.808219178 | 0 | 59 | MALE | Stage III | -0.189542085 | 0.969116716 |
| TCGA-22-4599 | 3.180821918 | 1 | 73 | FEMALE | Stage I | -0.188394549 | 0.645686743 |
| TCGA-68-A59I | 1.347945205 | 0 | 73 | FEMALE | Stage III | -0.188198553 | 0.935910486 |
| TCGA-85-8049 | 1.58630137 | 0 | 57 | MALE | Stage I | -0.187701071 | 0.647549546 |
| TCGA-43-A56V | 1.002739726 | 0 | 61 | MALE | Stage III | -0.187059802 | 1.000614341 |
| TCGA-60-2714 | 4.194520548 | 0 | 66 | FEMALE | Stage II | -0.187018154 | 0.676717396 |
| TCGA-33-4547 | 6.62739726 | 0 | 68 | MALE | Stage I | -0.1838483 | 0.772096376 |
| TCGA-77-8144 | 2.282191781 | 0 | 70 | MALE | Stage I | -0.181506339 | 0.79719057 |
| TCGA-39-5037 | 4.630136986 | 0 | 65 | MALE | Stage II | -0.180212661 | 0.86265722 |
| TCGA-58-8388 | 1.128767123 | 1 | 60 | MALE | Stage I | -0.179534886 | 0.67937406 |
| TCGA-NC-A5HQ | 1.22739726 | 1 | 70 | MALE | Stage III | -0.176332147 | 1.155512361 |
| TCGA-46-6025 | 0.887671233 | 0 | 71 | MALE | Stage II | -0.175071325 | 0.949533249 |
| TCGA-39-5011 | 11.10410959 | 0 | 70 | FEMALE | Stage I | -0.167712178 | 0.615440273 |
| TCGA-66-2770 | 1.917808219 | 0 | 79 | MALE | Stage I | -0.163673456 | 0.920597996 |
| TCGA-77-8143 | 2.2 | 1 | 76 | MALE | Stage III | -0.163054711 | 1.271881092 |
| TCGA-NK-A5CX | 0.304109589 | 0 | 73 | MALE | Stage II | -0.160702054 | 0.980394384 |
| TCGA-34-5232 | 6.769863014 | 0 | 75 | FEMALE | Stage II | -0.159043118 | 0.781475223 |
| TCGA-66-2782 | 1 | 1 | 71 | MALE | Stage II | -0.15513834 | 0.949533249 |
| TCGA-37-5819 | 0.282191781 | 0 | 64 | MALE | Stage III | -0.154559877 | 1.049790602 |
| TCGA-85-7950 | 1.578082192 | 0 | 46 | MALE | Stage I | -0.153352094 | 0.92285316 |
| TCGA-60-2724 | 1.964383562 | 0 | 47 | MALE | Stage III | -0.152193237 | 1.359221908 |
| TCGA-66-2793 | 0.838356164 | 1 | 68 | MALE | Stage III | -0.148959196 | 1.901700273 |
| TCGA-33-4533 | 11.14520548 | 0 | 76 | FEMALE | Stage I | -0.148354377 | 1.151107647 |
| TCGA-39-5035 | 5.698630137 | 0 | 72 | FEMALE | Stage I | -0.144696164 | 1.079778466 |
| TCGA-85-A511 | 1.246575342 | 1 | 62 | MALE | Stage II | -0.140544487 | 1.397205915 |
| TCGA-66-2800 | 4.087671233 | 0 | 70 | MALE | Stage III | -0.136659733 | 1.963508145 |
| TCGA-70-6722 | 1.005479452 | 0 | 47 | MALE | Stage III | -0.133643446 | 1.359221908 |
| TCGA-22-5482 | 0.978082192 | 1 | 81 | MALE | Stage I | -0.13351089 | 1.615171992 |
| TCGA-46-3768 | 0.819178082 | 1 | 58 | MALE | Stage III | -0.13063247 | 1.620648669 |
| TCGA-56-8082 | 1.246575342 | 0 | 80 | FEMALE | Stage II | -0.129564925 | 1.438467057 |

| | | | | | | | |
|---|---|---|---|---|---|---|---|
| TCGA-63-A5MI | 4.887671233 | 0 | 65 | MALE | Stage III | -0.129127352 | 1.812617261 |
| TCGA-85-8287 | 0.063013699 | 1 | 72 | MALE | Stage I | -0.128782528 | 1.398655967 |
| TCGA-96-8170 | 1.454794521 | 0 | 75 | FEMALE | Stage II | -0.127441227 | 1.327924319 |
| TCGA-66-2785 | 0.164383562 | 0 | 65 | MALE | Stage I | -0.125874554 | 1.250528714 |
| TCGA-18-3419 | 7.701369863 | 0 | 73 | MALE | Stage II | -0.123939023 | 1.665938352 |
| TCGA-66-2790 | 1.915068493 | 0 | 72 | MALE | Stage II | -0.123374568 | 1.639508253 |
| TCGA-58-A46L | 4.720547945 | 0 | 73 | MALE | Stage III | -0.123053306 | 2.060006851 |
| TCGA-56-1622 | 2.41369863 | 1 | 58 | MALE | Stage I | -0.122814387 | 1.118089152 |
| TCGA-34-5239 | 5.024657534 | 0 | 75 | MALE | Stage III | -0.120176676 | 2.126959905 |
| TCGA-60-2695 | 1.75890411 | 0 | 74 | FEMALE | Stage I | -0.117514844 | 1.11487275 |
| TCGA-22-0944 | 0.610958904 | 1 | 61 | MALE | Stage I | -0.116347632 | 1.173038838 |
| TCGA-60-2723 | 2.991780822 | 0 | 74 | FEMALE | Stage I | -0.11607106 | 1.11487275 |
| TCGA-56-A4BW | 1.602739726 | 0 | 55 | MALE | Stage II | -0.115447316 | 1.249232232 |
| TCGA-18-3416 | 2.665753425 | 1 | 83 | MALE | Stage II | -0.115424712 | 1.954844057 |
| TCGA-21-1079 | 2.643835616 | 1 | 71 | MALE | Stage III | -0.111969222 | 1.995161365 |
| TCGA-66-2753 | 0.084931507 | 0 | 69 | MALE | Stage I | -0.110264892 | 1.3331375 |
| TCGA-66-2773 | 0.252054795 | 1 | 69 | MALE | Stage I | -0.107898758 | 1.3331375 |
| TCGA-56-8304 | 0.290410959 | 0 | 73 | FEMALE | Stage I | -0.106298114 | 1.097185302 |
| TCGA-18-3417 | 3.005479452 | 1 | 65 | MALE | Stage IV | -0.102767009 | 4.032535434 |
| TCGA-85-8580 | 3.049315068 | 0 | 52 | FEMALE | Stage I | -0.102520184 | 0.784202601 |
| TCGA-85-7843 | 0.095890411 | 0 | 50 | MALE | Stage II | -0.101455078 | 1.153231736 |
| TCGA-22-4595 | 2.010958904 | 1 | 57 | MALE | Stage III | -0.101426005 | 1.594937091 |
| TCGA-NK-A5D1 | 1.4 | 0 | 57 | MALE | Stage II | -0.101005199 | 1.289833996 |
| TCGA-85-8351 | 1.397260274 | 0 | 72 | MALE | Stage II | -0.100325194 | 1.639508253 |
| TCGA-94-A4VJ | 1.178082192 | 0 | 71 | FEMALE | Stage I | -0.099819545 | 1.062647789 |
| TCGA-34-5241 | 1.410958904 | 1 | 79 | MALE | Stage I | -0.099251728 | 1.564329145 |
| TCGA-33-4583 | 12.60547945 | 1 | 73 | MALE | Stage I | -0.098424118 | 1.421203347 |
| TCGA-92-7341 | 0.290410959 | 0 | 71 | MALE | Stage I | -0.097538694 | 1.376466301 |
| TCGA-39-5024 | 6.876712329 | 0 | 65 | FEMALE | Stage III | -0.096843265 | 1.399361338 |
| TCGA-66-2783 | 2.079452055 | 0 | 67 | MALE | Stage III | -0.096350822 | 1.871529813 |
| TCGA-90-A59Q | 0.882191781 | 1 | 61 | FEMALE | Stage II | -0.095732493 | 1.061546092 |
| TCGA-22-5474 | 1.219178082 | 1 | 74 | MALE | Stage I | -0.094169147 | 1.444114208 |
| TCGA-56-7579 | 2.605479452 | 1 | 61 | MALE | Stage III | -0.092138768 | 1.70029718 |
| TCGA-58-A46J | 7.093150685 | 0 | 64 | MALE | Stage II | -0.086797774 | 1.442617027 |
| TCGA-66-2778 | 1.583561644 | 0 | 68 | FEMALE | Stage III | -0.086381438 | 1.468134447 |
| TCGA-43-A475 | 0.810958904 | 0 | 67 | FEMALE | Stage II | -0.084887431 | 1.168451717 |
| TCGA-NC-A5HI | 4.775342466 | 0 | 68 | FEMALE | Stage I | -0.078431061 | 1.012869247 |
| TCGA-34-2596 | 0.219178082 | 1 | 70 | MALE | Stage II | -0.076578288 | 1.587899342 |
| TCGA-85-8071 | 2.232876712 | 0 | 52 | MALE | Stage II | -0.074514067 | 1.190713352 |
| TCGA-85-8276 | 2.876712329 | 0 | 62 | MALE | Stage II | -0.071456351 | 1.397205915 |
| TCGA-66-2763 | 0.082191781 | 0 | 63 | FEMALE | Stage I | -0.06968737 | 0.935032679 |
| TCGA-33-6738 | 5.279452055 | 0 | 80 | MALE | Stage III | -0.067369958 | 2.304018166 |
| TCGA-22-4594 | 4.02739726 | 1 | 60 | FEMALE | Stage III | -0.062574812 | 1.291823781 |

| | | | | | | | |
|---|---|---|---|---|---|---|---|
| TCGA-66-2780 | 1.002739726 | 1 | 65 | MALE | Stage I | -0.060864938 | 1.250528714 |
| TCGA-51-6867 | 5.084931507 | 1 | 72 | FEMALE | Stage I | -0.058941392 | 1.079778466 |
| TCGA-39-5031 | 5.043835616 | 1 | 76 | FEMALE | Stage I | -0.058902226 | 1.151107647 |
| TCGA-85-6175 | 0.805479452 | 1 | 63 | FEMALE | Stage II | -0.058875226 | 1.096047799 |
| TCGA-18-3407 | 0.37260274 | 1 | 72 | MALE | Stage I | -0.0566735 | 1.398655967 |
| TCGA-18-3411 | 9.797260274 | 0 | 63 | FEMALE | Stage III | -0.05168652 | 1.355311841 |
| TCGA-60-2706 | 7.726027397 | 0 | 58 | MALE | Stage I | -0.050481358 | 1.118089152 |
| TCGA-51-4079 | 0.032876712 | 1 | 73 | FEMALE | Stage I | -0.043011016 | 1.097185302 |
| TCGA-33-4587 | 4.536986301 | 1 | 63 | FEMALE | Stage I | -0.041824285 | 0.935032679 |
| TCGA-22-1011 | 0.145205479 | 1 | 73 | MALE | Stage I | -0.040959044 | 1.421203347 |
| TCGA-92-7340 | 0.224657534 | 0 | 45 | FEMALE | Stage II | -0.039425656 | 0.8218901 |
| TCGA-96-8169 | 1.526027397 | 0 | 67 | FEMALE | Stage I | -0.038132098 | 0.996800085 |
| TCGA-02-A52N | 2.756164384 | 1 | 78 | MALE | Stage I | -0.037248036 | 1.539511075 |
| TCGA-18-3410 | 0.4 | 1 | 81 | MALE | Stage II | -0.036855266 | 1.893308915 |
| TCGA-68-7757 | 0.578082192 | 0 | 74 | MALE | Stage I | -0.035355533 | 1.444114208 |
| TCGA-22-4607 | 1.608219178 | 1 | 75 | MALE | Stage I | -0.034721732 | 1.467394409 |
| TCGA-39-5021 | 5.715068493 | 1 | 70 | MALE | Stage I | -0.032749411 | 1.354628673 |
| TCGA-96-7544 | 5.917808219 | 1 | 83 | MALE | Stage II | -0.030914029 | 1.954844057 |
| TCGA-85-A50Z | 1.350684932 | 0 | 57 | MALE | Stage II | -0.028961255 | 1.289833996 |
| TCGA-34-5928 | 3.276712329 | 0 | 83 | FEMALE | Stage II | -0.028623839 | 1.509162058 |
| TCGA-60-2707 | 1.82739726 | 1 | 70 | MALE | Stage I | -0.026374199 | 1.354628673 |
| TCGA-66-2794 | 4.506849315 | 0 | 64 | MALE | Stage III | -0.023451127 | 1.783860102 |
| TCGA-L3-A4E7 | 1.073972603 | 0 | 71 | MALE | Stage I | -0.022260407 | 1.376466301 |
| TCGA-66-2795 | 0.334246575 | 0 | 68 | MALE | Stage III | -0.021947591 | 1.901700273 |
| TCGA-85-A4JC | 1.654794521 | 1 | 84 | MALE | Stage II | -0.021562181 | 1.986357605 |
| TCGA-77-8145 | 0.580821918 | 1 | 73 | MALE | Stage III | -0.02086661 | 2.060006851 |
| TCGA-98-8022 | 2.556164384 | 1 | 61 | MALE | Stage I | -0.012455873 | 1.173038838 |
| TCGA-22-5485 | 2.509589041 | 1 | 58 | FEMALE | Stage I | -0.011660336 | 0.863177663 |
| TCGA-68-7756 | 0.553424658 | 0 | 84 | MALE | Stage III | -0.011487905 | 2.456219505 |
| TCGA-43-6647 | 2.073972603 | 0 | 69 | FEMALE | Stage II | -0.009281093 | 1.206428003 |
| TCGA-77-8138 | 1.476712329 | 1 | 74 | MALE | Stage I | -0.004832876 | 1.444114208 |
| TCGA-22-5480 | 5.945205479 | 1 | 66 | FEMALE | Stage I | -0.002023118 | 0.980985859 |
| TCGA-77-8130 | 13.05479452 | 0 | 69 | MALE | Stage II | -0.000195227 | 1.562707331 |
| TCGA-63-A5MR | 7.44109589 | 0 | 70 | FEMALE | Stage I | 0.003249061 | 1.04578889 |
| TCGA-66-2759 | 2.087671233 | 0 | 66 | MALE | Stage III | 0.004764708 | 1.841838007 |
| TCGA-56-5898 | 1.520547945 | 0 | 69 | MALE | Stage I | 0.005684477 | 1.3331375 |
| TCGA-90-7964 | 1.17260274 | 0 | 70 | MALE | Stage I | 0.007652144 | 1.354628673 |
| TCGA-37-A5EM | 2.375342466 | 0 | 49 | MALE | Stage II | 0.019199514 | 1.134935723 |
| TCGA-52-7622 | 2.361643836 | 0 | 62 | FEMALE | Stage I | 0.019912508 | 0.920198393 |
| TCGA-56-A4ZK | 1.561643836 | 0 | 76 | FEMALE | Stage I | 0.020490441 | 1.151107647 |
| TCGA-LA-A7SW | 1.117808219 | 1 | 71 | MALE | Stage III | 0.02273559 | 1.995161365 |
| TCGA-39-5022 | 4.6 | 1 | 76 | MALE | Stage I | 0.024953507 | 1.491049904 |
| TCGA-68-8251 | 1.112328767 | 0 | 78 | MALE | Stage I | 0.035829647 | 1.539511075 |

| | | | | | | | |
|---|---|---|---|---|---|---|---|
| TCGA-66-2765 | 0.167123288 | 0 | 64 | MALE | Stage I | 0.040448885 | 1.230689085 |
| TCGA-77-7338 | 0.01369863 | 1 | 64 | MALE | Stage I | 0.040829215 | 1.230689085 |
| TCGA-63-A5MS | 6.523287671 | 0 | 78 | MALE | Stage I | 0.042153671 | 1.539511075 |
| TCGA-22-4604 | 1.093150685 | 1 | 73 | MALE | Stage II | 0.045666472 | 1.665938352 |
| TCGA-43-A56U | 1.183561644 | 0 | 76 | FEMALE | Stage I | 0.053347502 | 1.151107647 |
| TCGA-22-0940 | 1.832876712 | 1 | 71 | MALE | Stage II | 0.053581557 | 1.613497467 |
| TCGA-60-2712 | 0.750684932 | 1 | 79 | FEMALE | Stage II | 0.05565273 | 1.415645788 |
| TCGA-85-A50M | 2.263013699 | 1 | 47 | MALE | Stage II | 0.056006883 | 1.099209891 |
| TCGA-60-2719 | 3.553424658 | 0 | 83 | FEMALE | Stage I | 0.056231824 | 1.287458305 |
| TCGA-85-8048 | 2.095890411 | 0 | 62 | MALE | Stage I | 0.05719325 | 1.191949101 |
| TCGA-85-8584 | 1.049315068 | 1 | 71 | MALE | Stage II | 0.058368419 | 1.613497467 |
| TCGA-60-2697 | 1.019178082 | 1 | 41 | MALE | Stage II | 0.058766283 | 0.998639437 |
| TCGA-85-8353 | 0.257534247 | 1 | 72 | MALE | Stage III | 0.059081372 | 2.027324858 |
| TCGA-58-8392 | 1.37260274 | 1 | 70 | MALE | Stage I | 0.064122456 | 1.354628673 |
| TCGA-34-2608 | 2.739726027 | 1 | 84 | MALE | Stage I | 0.068915744 | 1.694551345 |
| TCGA-21-1077 | 2.898630137 | 1 | 64 | MALE | Stage II | 0.069464118 | 1.442617027 |
| TCGA-66-2791 | 0.419178082 | 1 | 66 | MALE | Stage III | 0.070509238 | 1.841838007 |
| TCGA-58-8393 | 2.898630137 | 0 | 68 | FEMALE | Stage I | 0.073239443 | 1.012869247 |
| TCGA-77-8156 | 3.030136986 | 0 | 60 | MALE | Stage I | 0.073403755 | 1.154428586 |
| TCGA-98-7454 | 1.78630137 | 0 | 73 | MALE | Stage I | 0.074648482 | 1.421203347 |
| TCGA-37-4132 | 0.621917808 | 0 | 61 | FEMALE | Stage IV | 0.075920807 | 2.920254321 |
| TCGA-18-3406 | 1.016438356 | 1 | 67 | MALE | Stage I | 0.078593009 | 1.291172615 |
| TCGA-56-7222 | 1.539726027 | 1 | 60 | MALE | Stage I | 0.079240111 | 1.154428586 |
| TCGA-98-A53H | 1.693150685 | 0 | 76 | FEMALE | Stage I | 0.081535292 | 1.151107647 |
| TCGA-43-8118 | 0.243835616 | 1 | 55 | FEMALE | Stage I | 0.082401507 | 0.822743076 |
| TCGA-34-5234 | 6.221917808 | 0 | 71 | FEMALE | Stage I | 0.086363941 | 1.062647789 |
| TCGA-85-6561 | 3.353424658 | 0 | 66 | MALE | Stage I | 0.087786924 | 1.270688172 |
| TCGA-56-8083 | 0.410958904 | 0 | 56 | MALE | Stage I | 0.09612653 | 1.082893621 |
| TCGA-56-8626 | 0.82739726 | 1 | 59 | MALE | Stage I | 0.096643767 | 1.136113585 |
| TCGA-85-6560 | 3.449315068 | 0 | 59 | MALE | Stage II | 0.101105929 | 1.331755374 |
| TCGA-39-5034 | 3.032876712 | 1 | 73 | FEMALE | Stage II | 0.103204833 | 1.286123537 |
| TCGA-85-A4PA | 2.030136986 | 0 | 61 | MALE | Stage I | 0.118718811 | 1.173038838 |
| TCGA-43-2578 | 1.873972603 | 1 | 59 | FEMALE | Stage I | 0.121486504 | 0.877092733 |
| TCGA-77-A5GH | 3.238356164 | 0 | 81 | MALE | Stage I | 0.127083763 | 1.615171992 |
| TCGA-22-5481 | 6.6 | 1 | 72 | FEMALE | Stage II | 0.131961121 | 1.265719197 |
| TCGA-92-8063 | 0.334246575 | 0 | 52 | MALE | Stage III | 0.133653309 | 1.472370006 |
| TCGA-21-5787 | 0.901369863 | 1 | 65 | MALE | Stage III | 0.138190833 | 1.812617261 |
| TCGA-39-5039 | 1.490410959 | 1 | 76 | MALE | Stage II | 0.139015406 | 1.747812673 |
| TCGA-22-5489 | 5.238356164 | 1 | 64 | MALE | Stage I | 0.141003698 | 1.230689085 |
| TCGA-77-7138 | 0.931506849 | 1 | 67 | MALE | Stage I | 0.144482171 | 1.291172615 |
| TCGA-33-6737 | 1.646575342 | 1 | 71 | MALE | Stage III | 0.147427875 | 1.995161365 |
| TCGA-22-1002 | 0.35890411 | 1 | 69 | MALE | Stage I | 0.151090794 | 1.3331375 |
| TCGA-37-3783 | 0.334246575 | 0 | 51 | MALE | Stage III | 0.151797596 | 1.449010868 |

| | | | | | | | |
|---|---|---|---|---|---|---|---|
| TCGA-66-2767 | 0.167123288 | 0 | 62 | MALE | Stage III | 0.153244937 | 1.727707242 |
| TCGA-56-8628 | 1.687671233 | 0 | 78 | MALE | Stage I | 0.163299248 | 1.539511075 |
| TCGA-66-2744 | 0.082191781 | 0 | 71 | MALE | Stage II | 0.164483092 | 1.613497467 |
| TCGA-21-5784 | 3.473972603 | 0 | 80 | FEMALE | Stage I | 0.169094919 | 1.227148768 |
| TCGA-43-6773 | 0.317808219 | 1 | 76 | MALE | Stage II | 0.172656305 | 1.747812673 |
| TCGA-22-1005 | 5.350684932 | 1 | 63 | MALE | Stage I | 0.174966928 | 1.211164212 |
| TCGA-43-6143 | 1.915068493 | 0 | 70 | MALE | Stage I | 0.180832826 | 1.354628673 |
| TCGA-92-8065 | 0.191780822 | 0 | 74 | FEMALE | Stage II | 0.181410043 | 1.30685681 |
| TCGA-34-8455 | 0.336986301 | 1 | 67 | MALE | Stage IV | 0.183787384 | 4.163598377 |
| TCGA-60-2696 | 0.298630137 | 1 | 76 | FEMALE | Stage II | 0.185364113 | 1.349331452 |
| TCGA-77-8008 | 7.230136986 | 1 | 68 | MALE | Stage I | 0.187251384 | 1.311987284 |
| TCGA-77-8136 | 3.257534247 | 1 | 74 | FEMALE | Stage II | 0.18864651 | 1.30685681 |
| TCGA-60-2713 | 4.742465753 | 0 | 64 | MALE | Stage I | 0.190223704 | 1.230689085 |
| TCGA-46-3767 | 1.084931507 | 0 | 76 | MALE | Stage I | 0.191412017 | 1.491049904 |
| TCGA-94-7557 | 0.01369863 | 1 | 73 | MALE | Stage I | 0.191895096 | 1.421203347 |
| TCGA-60-2720 | 0.265753425 | 0 | 60 | FEMALE | Stage I | 0.192665401 | 0.891232124 |
| TCGA-NC-A5HE | 6.4 | 0 | 60 | MALE | Stage II | 0.193701628 | 1.353224266 |
| TCGA-77-7337 | 8.912328767 | 1 | 65 | MALE | Stage II | 0.201489202 | 1.465873092 |
| TCGA-58-8386 | 0.002739726 | 1 | 75 | MALE | Stage IV | 0.205253701 | 4.731854522 |
| TCGA-56-5897 | 1.035616438 | 0 | 74 | MALE | Stage I | 0.208308113 | 1.444114208 |
| TCGA-56-A49D | 1.745205479 | 0 | 67 | MALE | Stage III | 0.213649379 | 1.871529813 |
| TCGA-52-7811 | 0.728767123 | 1 | 67 | MALE | Stage I | 0.216028572 | 1.291172615 |
| TCGA-63-A5MT | 1.364383562 | 0 | 74 | MALE | Stage II | 0.218054189 | 1.692794524 |
| TCGA-22-4605 | 2.668493151 | 1 | 78 | FEMALE | Stage I | 0.221327331 | 1.188520227 |
| TCGA-66-2771 | 1.583561644 | 0 | 60 | MALE | Stage II | 0.222159811 | 1.353224266 |
| TCGA-NC-A5HT | 2.202739726 | 0 | 69 | MALE | Stage III | 0.225600479 | 1.932357104 |
| TCGA-66-2786 | 2.164383562 | 0 | 68 | FEMALE | Stage I | 0.22971514 | 1.012869247 |
| TCGA-39-5028 | 0.142465753 | 1 | 75 | MALE | Stage III | 0.230359837 | 2.126959905 |
| TCGA-77-A5G1 | 11.03013699 | 0 | 75 | MALE | Stage III | 0.231687492 | 2.126959905 |
| TCGA-98-A53B | 0.167123288 | 1 | 69 | MALE | Stage I | 0.233342821 | 1.3331375 |
| TCGA-85-8355 | 0.167123288 | 0 | 63 | MALE | Stage I | 0.233381996 | 1.211164212 |
| TCGA-85-A513 | 2.493150685 | 0 | 60 | FEMALE | Stage I | 0.247436377 | 0.891232124 |
| TCGA-22-1000 | 1.243835616 | 1 | 76 | MALE | Stage I | 0.248496188 | 1.491049904 |
| TCGA-22-5472 | 5.410958904 | 1 | 67 | MALE | Stage I | 0.251730723 | 1.291172615 |
| TCGA-85-8582 | 3.178082192 | 0 | 49 | MALE | Stage I | 0.253793639 | 0.968207765 |
| TCGA-34-5236 | 0.756164384 | 1 | 60 | MALE | Stage II | 0.255574282 | 1.353224266 |
| TCGA-98-8023 | 1.778082192 | 0 | 70 | MALE | Stage III | 0.258509365 | 1.963508145 |
| TCGA-96-7545 | 4.756164384 | 1 | 73 | MALE | Stage I | 0.267865708 | 1.421203347 |
| TCGA-18-3409 | 10.26575342 | 0 | 74 | MALE | Stage I | 0.274904146 | 1.444114208 |
| TCGA-34-8454 | 3.232876712 | 0 | 62 | FEMALE | Stage III | 0.275744756 | 1.333809829 |
| TCGA-43-8115 | 1.115068493 | 0 | 72 | FEMALE | Stage II | 0.277851985 | 1.265719197 |
| TCGA-22-5492 | 1.38630137 | 1 | 73 | FEMALE | Stage III | 0.279896428 | 1.590348943 |
| TCGA-60-2709 | 4.123287671 | 0 | 69 | MALE | Stage I | 0.280510803 | 1.3331375 |

| | | | | | | | |
|---|---|---|---|---|---|---|---|
| TCGA-56-8503 | 0.112328767 | 0 | 76 | FEMALE | Stage II | 0.283355848 | 1.349331452 |
| TCGA-60-2710 | 5.545205479 | 0 | 67 | FEMALE | Stage II | 0.284461445 | 1.168451717 |
| TCGA-66-2769 | 0.589041096 | 1 | 75 | MALE | Stage III | 0.290715273 | 2.126959905 |
| TCGA-43-2576 | 3.350684932 | 0 | 62 | FEMALE | Stage III | 0.290985154 | 1.333809829 |
| TCGA-56-A4ZJ | 1.753424658 | 0 | 75 | FEMALE | Stage I | 0.291058861 | 1.132845333 |
| TCGA-66-2755 | 0.076712329 | 0 | 63 | MALE | Stage I | 0.295274568 | 1.211164212 |
| TCGA-56-6545 | 1.824657534 | 0 | 77 | FEMALE | Stage I | 0.295664277 | 1.169664363 |
| TCGA-66-2777 | 0.167123288 | 0 | 71 | MALE | Stage I | 0.297570801 | 1.376466301 |
| TCGA-22-4609 | 0.797260274 | 1 | 81 | MALE | Stage I | 0.298807893 | 1.615171992 |
| TCGA-46-3766 | 1.01369863 | 0 | 62 | FEMALE | Stage I | 0.311162188 | 0.920198393 |
| TCGA-90-A4EE | 1.884931507 | 0 | 53 | MALE | Stage II | 0.311439095 | 1.209908542 |
| TCGA-39-5029 | 2.02739726 | 1 | 67 | MALE | Stage III | 0.342016828 | 1.871529813 |
| TCGA-43-7658 | 6.515068493 | 1 | 75 | FEMALE | Stage I | 0.349297164 | 1.132845333 |
| TCGA-XC-AA0X | 0.016438356 | 1 | 77 | FEMALE | Stage I | 0.349754163 | 1.169664363 |
| TCGA-60-2725 | 2.235616438 | 0 | 74 | MALE | Stage I | 0.359197408 | 1.444114208 |
| TCGA-63-A5MN | 0.945205479 | 1 | 78 | FEMALE | Stage II | 0.364833385 | 1.393186579 |
| TCGA-77-7335 | 5.843835616 | 1 | 62 | FEMALE | Stage III | 0.370238368 | 1.333809829 |
| TCGA-85-8481 | 0.646575342 | 1 | 70 | MALE | Stage II | 0.375985756 | 1.587899342 |
| TCGA-66-2788 | 1.915068493 | 0 | 56 | MALE | Stage I | 0.382016129 | 1.082893621 |
| TCGA-66-2734 | 3.591780822 | 0 | 62 | FEMALE | Stage I | 0.388712087 | 0.920198393 |
| TCGA-60-2698 | 0.852054795 | 1 | 62 | MALE | Stage II | 0.389502037 | 1.397205915 |
| TCGA-37-3789 | 0.035616438 | 0 | 65 | MALE | Stage I | 0.390126559 | 1.250528714 |
| TCGA-60-2715 | 2.945205479 | 1 | 51 | MALE | Stage I | 0.394650336 | 0.999675847 |
| TCGA-56-8309 | 1.17260274 | 0 | 66 | MALE | Stage I | 0.395780577 | 1.270688172 |
| TCGA-85-8666 | 1.882191781 | 1 | 65 | MALE | Stage I | 0.399235587 | 1.250528714 |
| TCGA-68-8250 | 0.668493151 | 0 | 66 | MALE | Stage I | 0.399692785 | 1.270688172 |
| TCGA-37-3792 | 0.032876712 | 0 | 69 | MALE | Stage I | 0.4000839 | 1.3331375 |
| TCGA-98-A53D | 1.767123288 | 1 | 68 | MALE | Stage II | 0.401914131 | 1.537914992 |
| TCGA-77-A5FZ | 10.51506849 | 1 | 64 | MALE | Stage III | 0.403614353 | 1.783860102 |
| TCGA-98-8020 | 0.230136986 | 1 | 56 | FEMALE | Stage III | 0.412982175 | 1.211775028 |
| TCGA-33-4589 | 0.128767123 | 1 | 62 | FEMALE | Stage II | 0.433062413 | 1.078659009 |
| TCGA-NC-A5HJ | 1.145205479 | 1 | 59 | MALE | Stage II | 0.439105537 | 1.331755374 |
| TCGA-22-1017 | 4.068493151 | 1 | 62 | MALE | Stage I | 0.447182328 | 1.191949101 |
| TCGA-56-7822 | 1.457534247 | 1 | 75 | MALE | Stage II | 0.459522405 | 1.720083638 |
| TCGA-39-5030 | 0.161643836 | 1 | 81 | FEMALE | Stage III | 0.470838637 | 1.807402913 |
| TCGA-21-1076 | 5.073972603 | 0 | 54 | FEMALE | Stage I | 0.490729766 | 0.809690263 |
| TCGA-98-A53C | 2.252054795 | 0 | 77 | FEMALE | Stage I | 0.494790593 | 1.169664363 |
| TCGA-66-2768 | 0.167123288 | 0 | 57 | MALE | Stage II | 0.496106594 | 1.289833996 |
| TCGA-60-2704 | 3.161643836 | 1 | 73 | MALE | Stage II | 0.505872771 | 1.665938352 |
| TCGA-43-2581 | 3.221917808 | 0 | 47 | FEMALE | Stage III | 0.508937803 | 1.04933492 |
| TCGA-66-2789 | 0.336986301 | 1 | 73 | MALE | Stage III | 0.518929416 | 2.060006851 |
| TCGA-56-8625 | 0.863013699 | 1 | 66 | FEMALE | Stage III | 0.527111156 | 1.42192009 |
| TCGA-56-7731 | 0.008219178 | 1 | 66 | FEMALE | Stage I | 0.562016794 | 0.980985859 |

| | | | | | | | |
|---|---|---|---|---|---|---|---|
| TCGA-56-8201 | 1.087671233 | 1 | 74 | MALE | Stage II | 0.562042925 | 1.692794524 |
| TCGA-85-8352 | 0.44109589 | 1 | 67 | MALE | Stage III | 0.566821942 | 1.871529813 |
| TCGA-21-5782 | 2.635616438 | 1 | 68 | FEMALE | Stage I | 0.624288521 | 1.012869247 |
| TCGA-22-4596 | 0.046575342 | 1 | 69 | FEMALE | Stage I | 0.626493749 | 1.029197457 |
| TCGA-43-5668 | 1.531506849 | 1 | 78 | MALE | Stage II | 0.644324259 | 1.804618987 |
| TCGA-77-8007 | 0.542465753 | 1 | 68 | MALE | Stage II | 0.685017421 | 1.537914992 |
| TCGA-90-6837 | 2.076712329 | 0 | 64 | MALE | Stage II | 0.691114657 | 1.442617027 |
| TCGA-02-A52Q | 0.309589041 | 1 | 44 | FEMALE | Stage III | 0.696062357 | 1.000180005 |
| TCGA-22-1016 | 2.252054795 | 1 | 65 | MALE | Stage I | 0.839645562 | 1.250528714 |
| TCGA-56-8623 | 1.895890411 | 1 | 71 | MALE | Stage I | 0.870522664 | 1.376466301 |
| TCGA-77-8131 | 1.049315068 | 1 | 72 | MALE | Stage I | 0.875916039 | 1.398655967 |
| TCGA-43-6771 | 0.454794521 | 1 | 85 | MALE | Stage I | 1.094217548 | 1.72186878 |
| TCGA-39-5040 | 1.421917808 | 1 | 59 | MALE | Stage III | 1.414273682 | 1.646774738 |
